# A systematic review of prospective evidence linking non-alcoholic fermented food consumption with lower mortality risk

**DOI:** 10.3389/fnut.2025.1657100

**Published:** 2025-11-03

**Authors:** Diana Paveljšek, Eugenia Pertziger, Anthony Fardet, Demosthenes Basilis Panagiotakos, Isabelle Savary-Auzeloux, Signe Adamberg, Elena Peñas, Juana Frias, Anastasia Ntantou, Ioannis Diamantoglou, Julieta Domínguez-Soberanes, Sandrine Louis, Christophe Chassard, Smilja Praćer, Guy Vergères, Antonia Matalas

**Affiliations:** ^1^University of Ljubljana, Biotechnical Faculty, Ljubljana, Slovenia; ^2^Research Division Microbial Food Systems, Agroscope, Bern, Switzerland; ^3^Department of Epidemiology and Health Systems, Center for Primary Care and Public Health (Unisanté), University of Lausanne, Lausanne, Switzerland; ^4^Université Clermont Auvergne, INRAE, UNH, Unité de Nutrition Humaine, Clermont-Ferrand, France; ^5^School of Health Sciences and Education, Harokopio University of Athens, Athens, Greece; ^6^Department of Chemistry and Biotechnology, Tallinn University of Technology, Tallinn, Estonia; ^7^Institute of Food Science, Technology and Nutrition (ICTAN-CSIC), Madrid, Spain; ^8^Facultad de Ingeniería, Universidad Panamericana, Aguascalientes, Mexico; ^9^Department of Physiology and Biochemistry of Nutrition, Max Rubner-Institut, Karlsruhe, Germany; ^10^UCA, INRAE, VetAgro Sup, UMRF 0545, Aurillac, France; ^11^Institute for Biological Research Siniša Stanković, National Institute of the Republic of Serbia, University of Belgrade, Belgrade, Serbia

**Keywords:** fermented foods, all-cause mortality, cardiovascular mortality, cancer mortality, fermented dairy products, fermented soy, fermented meat, fermented vegetables

## Abstract

Fermented foods are consumed worldwide and are increasingly being studied for their potential health benefits. Although their consumption is widespread, their association with long-term health outcomes such as mortality risk remains unclear. The aim of this systematic review was to assess the association between the consumption of fermented foods and risk of all-cause, cardiovascular, and cancer-related mortality in generally healthy adult populations in accordance with the European Food Safety Authority (EFSA) framework for the substantiation of health claims. A comprehensive literature search identified prospective cohort studies from 1970 to 2025 that investigated the association between fermented food consumption and mortality outcomes. Risk of bias was assessed using the Newcastle-Ottawa Scale, and strength of evidence was evaluated based on study quality, sample size, and precision within a structured narrative framework that also classified the direction of association across categories. A complementary non-systematic review examined the compositional characteristics, mechanisms of action, and potential health risks associated with fermented foods. Fifty-two cohort studies were included. Fermented milk products (including yogurt), chocolate, and fermented soy products (particularly natto) suggested a modest inverse association with all-cause and cardiovascular mortality. Cheese was associated with reduced all-cause mortality in some studies, but it showed inconsistent effect on cardiovascular mortality. The evidence for cancer-related mortality was weaker, although yogurt and fermented milk displayed some protective trends. Evidence from a single cohort suggested a potential reduction in all-cause mortality with fermented vegetable consumption, whereas fermented meat suggested no clear association with mortality. Biological plausibility was supported by fermentation-derived compounds such as bioactive peptides, polyphenols, isoflavones, natto-kinase, and vitamin K2. Habitual consumption of certain fermented foods may be associated with modest reductions in mortality risk, but the current evidence remains insufficient to support EFSA-approved health claims. Randomized controlled trials are essential to demonstrate causality. While long-term trials with mortality endpoints are not feasible, studies targeting intermediate outcomes linked to mortality offer a practical alternative. These should be complemented by observational studies to capture long-term, real-world associations. Together, such efforts support the objectives of the COST Action PIMENTO (CA20128) in building a more robust evidence base on fermented foods and health.

**Systematic review registration:**

The protocol for this systematic review was registered with the Open Science Framework (OSF; registration ID: vg7f6; https://osf.io/vg7f6).

## 1 Introduction

Non-communicable diseases (NCDs) are the leading cause of death worldwide, accounting for more than 70% of all deaths (1). Diet is one of the most significant modifiable risk factors for NCDs, and certain food groups with potentially protective properties have gained increasing attention. Among these, fermented foods have gained renewed interest due to their unique biochemical composition and potential health benefits (2, 3).

According to the International Scientific Association for Probiotics and Prebiotics (ISAPP), fermented foods are defined as “foods produced by desired microbial growth and enzymatic conversion of food components” (4). Fermentation increases food safety, extends shelf life by inhibiting spoilage and pathogenic microorganisms, and can improve both nutritional value and digestibility (5, 6). During fermentation, microbial activity alters the pH and water activity of food by producing organic acids and other metabolites, while also generating bioactive compounds such as peptides, polyphenols, and vitamins with potential health benefits (7). Beyond their metabolic products, the live microorganisms found in many fermented foods can provide direct health benefits by modulating the gut microbiota and supporting immune function, even if only transiently during their passage through the gastrointestinal (GI) tract (6, 8). Importantly, not all fermented foods contain live microorganisms at the time of consumption, as processing methods such as heat treatment and filtration can eliminate viable microorganisms, but they can still exert health effects through retained microbial metabolites or structural components. Taken together, these characteristics highlight that fermented foods represent a highly diverse category, shaped by the type of substrate, the microbial consortia involved, and the specific fermentation and processing techniques used. They span a wide range of categories, including fermented dairy products, vegetables, grains, legumes, meat, fish, and beverages, and are deeply rooted in traditional diets across cultures. Globally, they are estimated to account for approximately one-third of the human diet (9). Recent data from the Swiss adult population indicate that fermented foods contribute over 20% of daily food intake and substantially enhance the intake of key nutrients, including calcium, vitamins A and B12, zinc, and saturated fat (10). Despite their ubiquity, fermented foods remain poorly characterized in food composition databases, especially with regard to their microbial content, making standardized dietary assessment difficult and limiting research on their health effects.

Existing evidence syntheses remain fragmented across individual fermented foods, limiting cross-categorical interpretation and structured consideration of gaps. To provide a comprehensive mapping, we conducted a systematic review as part of the COST Action CA20128 Promoting Innovation of Fermented Foods (PIMENTO) (11). Our aim was to assess whether the consumption of fermented foods is associated with all-cause mortality and cause-specific mortality in generally healthy adults, while placing this epidemiological evidence within the EFSA’s health-claim logic. Using a structured narrative synthesis, consistent with SWiM (Synthesis Without Meta-analysis) principles and including credibility grading (12, 13), we assessed the direction and strength of associations across major categories of fermented foods, including fermented meat products, fermented vegetable products, fermented dairy products (e.g., yogurt, cheese), soy-based legume products (e.g., natto, miso), fermented grain products (e.g., bread), and cocoa-derived foods (e.g., chocolate). This work advances the field by integrating prospective cohort evidence across multiple categories and applying a structured synthesis aligned with EFSA guidance on coherence and biological plausibility, without restricting the evidence base to only exposures suitable for pooling. Accordingly, the review provides decision-relevant context and contributes to the growing evidence that fermented foods may be associated with lower all-cause and cause-specific mortality.

## 2 Methods

### 2.1 Systematic review of human studies

#### 2.1.1 Study protocol

This systematic review was conducted following the PIMENTO study protocol (PIMENTO-SP-S5), which was developed as part of the COST Action CA20128 PIMENTO. The pre-defined protocol (14), grounded in Cochrane principles (15) and the methodological guidance of Muka et al. (16), was designed in alignment with EFSA guidelines for health claim applications (17, 18). It incorporates essential methodological components, including a clearly formulated research question, prospective registration in the Open Science Framework (OSF), and well-defined inclusion and exclusion criteria to guide study selection.

#### 2.1.2 Literature search

A systematic literature search was performed using the PubMed, Scopus, and Cochrane Library databases to identify observational studies published between 1 January 1970 and 31 August 2023, investigating the association between consumption of fermented foods and mortality risk. The search strategy included terms related to: (i) fermented foods (e.g., dairy products, meat, vegetables, cereals); (ii) mortality outcomes (e.g., death, mortality rate); and (iii) observational studies (e.g., cohort study, prospective study, longitudinal study). The query syntax of searching is shown in the Supplementary Table 1. In addition, the reference lists of relevant systematic reviews and meta-analyses, obtained with this search string, were hand-searched to identify any missing prospective studies. Only studies published in English were considered. A further search was conducted prior to submission of the manuscript, which included studies published since the last search in 2023 and up to 15th of January 2025.

#### 2.1.3 Study selection

The studies selection was conducted through an independent assessment by two reviewers for each publication using the CADIMA tool (19). This process was carried out in two stages: the title/abstract screening, followed by the full-text screening. Both stages were guided by clearly defined inclusion and exclusion criteria based on the PIO framework (population, intervention, outcome) to guarantee a systematic and transparent approach. Reviewers were trained using a consistency test (CT) to ensure uniformity in evaluations and minimize bias. Discrepancies in the evaluations, both in the title/abstract and full-text screening stages, were resolved through discussions, and when disagreements persisted, a third reviewer was consulted.

Eligible populations included apparently healthy adults at time of enrolling, while studies involving individuals with diagnosed pathological conditions (e.g., diabetes, hypertension), food poisoning, pregnant women or infants, were excluded. Interventions had to specifically assess the consumption of one or more fermented foods as part of the daily diet. Outcomes of interest were all-cause mortality and cause-specific mortality, expressed in terms such as “mortality rate,” “mortality risk” or “risk of fatal outcomes.” The outcomes analyzed included all-cause mortality, cardiovascular disease (CVD) mortality, all-cancer mortality, and mortality from GI, lung, and reproductive cancers, representing endpoints that were commonly reported across the majority of studies and for which sufficient data were available in the literature.

**FIGURE 1:**
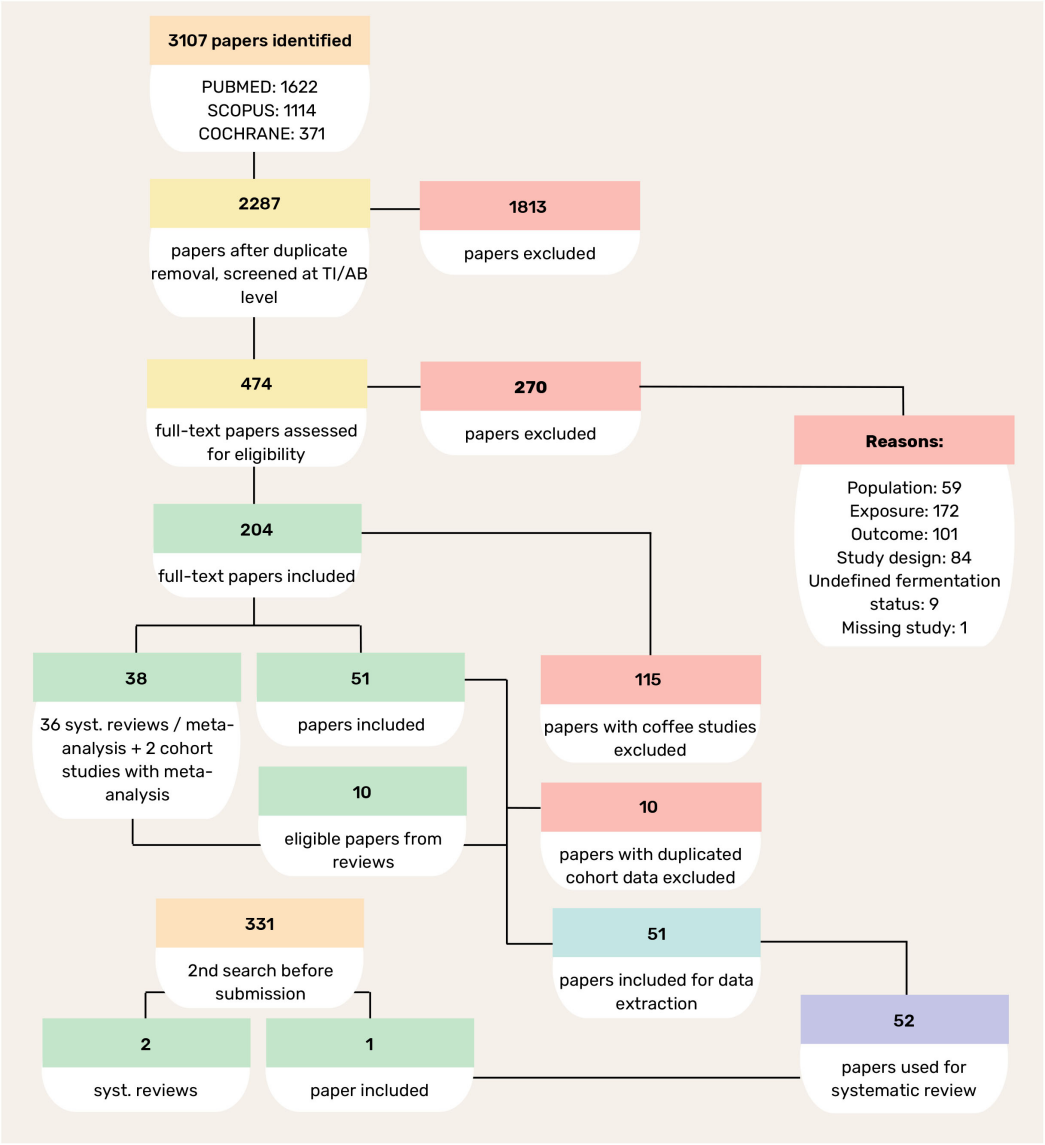
Flow diagram of study selection process.

Studies focusing on fermented alcoholic beverages with an alcohol content greater than 1.25%, as well as studies on certified probiotic or probiotic supplemented products (not derived from fermentation or unrelated to a fermented food matrix), or on isolated bioactive compounds were excluded. Furthermore, studies dealing with coffee consumption were excluded from the next steps as they typically focus on the effects of caffeine, rather than on the health effects of coffee in its entirety as a fermented food, while several recent reviews and meta-analyses have comprehensively addressed the relationship between coffee intake and mortality risk (20–22), rendering further analysis redundant. Articles focusing on foods with undefined or unexplained fermentation status (e.g., butter, tea, pickles) were also excluded. If multiple publications included the same cohort with identical exposures and outcomes, only the most recent publication was retained. These criteria, along with the additional verification of the retained studies, were independently reviewed and confirmed by two additional reviewers. The selection process was thoroughly documented using a PRISMA flowchart detailing the number of studies retrieved, the duplicates removed, the studies screened and the studies included in the final analysis (Figure 1).

#### 2.1.4 Data extraction and risk of bias assessment

The data extraction process was performed independently by two reviewers using standardized forms. Extracted data include key characteristics of each study, including population size, demographics, baseline health status, details of fermented food consumption (e.g., specific foods, quantities and frequency of consumption) as well as mortality outcomes, including hazard ratios (HRs), confidence intervals (CIs) and *p*-values of the model with the highest level of adjustment. Subgroup analyses (e.g., sex-specific findings) were included where available. Potential mechanisms of action of fermented foods as suggested by the study authors, were also recorded for the non-systematic part of this review.

Risk of bias was assessed independently by two reviewers using the Newcastle-Ottawa Scale (NOS) which is suitable for cohort studies (23). This assessment evaluated selection criteria (e.g., representativeness of the cohort, exposure), comparability of the cohort [e.g., adjustment for confounding factors such as age, sex, body mass index (BMI) and lifestyle factors] and outcome-related criteria (e.g., method of assessment, adequacy, and completeness of follow-up). Bias scores were merged, and any discrepancies were resolved through discussion or with the help of a third reviewer who conducted a final review to ensure consistency.

#### 2.1.5 Data analysis and data synthesis

Data synthesis was performed using a structured narrative approach, supported by the extraction of HRs and 95% CIs from prospective cohort studies reporting on the association between fermented food consumption and all-cause, CVD, and cancer-specific mortality. Exposure-outcome pairs were analyzed on the basis of highest versus lowest or no consumption category.

To provide a comprehensive, comparable map of fermented-food exposures, we prespecified a rule-based synthesis consistent with SWiM principles, integrating GRADE-based precision and umbrella-review credibility considerations (12, 13, 24–26). As not all food categories had multiple, comparable cohort studies, a pooled estimate across categories would not reflect the review question. Instead, we applied transparent rules to classify the indicative direction of effect and the strength of association, weighing consistency and study quality alongside effect estimates to support interpretable, cross-category comparisons. Specifically, we applied a rule-based framework to (i) summarize category-level estimates, (ii) examine confidence-interval patterns and cross-study consistency, and (iii) rate certainty using a narrative GRADE approach. Study quality and the overall consistency of the indicative direction of effect were incorporated to ensure transparent and reproducible interpretation. Full decision rules and category definitions are provided in the Supplementary Material.

In parallel with the indicative effect-direction synthesis, we annotated each food category in terms of microbial viability at the point of consumption at three levels: “Yes” (products that are usually consumed unheated and/or labeled with live cultures, such as yogurt/fermented milks, natto, unpasteurized fermented vegetables), “No” (products that are usually baked, roasted or pasteurized, such as sourdough bread, chocolate/cocoa products), and “Variable” (viability depends on processing or preparation, such as fermented vegetables where pasteurization is included; miso when primarily consumed as a hot soup) (6, 7, 10).

### 2.2 Non-systematic overview of supporting evidence

In the second part of this review, a non-systematic exploratory search was conducted to complement the systematic analysis and to contextualize the findings in light of observational literature, with a focus on plausible biological mechanisms and the compositional characteristics of the fermented foods studied. This section of the review explores the characteristics of fermented foods, including the fermentation process, microbial constituents, bioavailability of fermentation-derived compounds, safety considerations, and potential mechanisms of action. Bioavailability refers to the extent to which bioactive compounds produced during fermentation, such as peptides, polyphenols or vitamins, are absorbed and become available at the site of physiological activity, which is essential for exerting potential health effects. In relation to the latter, a number of possible mechanisms have been proposed to interpret the health benefits observed from the consumption of fermented foods that relate to specific physiological pathways.

## 3 Results and discussion

This systematic review is based on observational studies conducted in large cohorts with long follow-up periods. While this type of evidence does not establish causation, it offers valuable insights into potential associations and underlying mechanisms, thereby contributing to a more comprehensive understanding of the observed relationships. Due to limitations in the available data on certain fermented food-mortality associations and proposed mechanisms of action, some topics could only be addressed without in-depth analysis. Nonetheless, the review aims to draw well-reasoned conclusions by considering the totality of evidence, with particular attention to consistency, biological plausibility, and specificity of effects.

### 3.1 Identification of pertinent human efficacy studies

The study selection process is summarized in the flowchart (Figure 1). In total, 3,107 records were retrieved from PubMed, Scopus, and the Cochrane Library. After screening and eligibility assessment, studies were excluded primarily due to irrelevant exposures, outcomes focused on disease incidence rather than mortality, ineligible populations, or unsuitable study designs. Ultimately, 52 prospective cohort studies were considered for data extraction and synthesis, representing diverse geographic and cultural populations. This broad inclusion improves the evidence base, and allows for a comprehensive assessment of the association between fermented food consumption and mortality risk. In addition to the large cohorts that have been analyzed in several publications (EPIC, MONICA, NHS&HPFS, Adventist Health Study, Lutheran Brotherhood Study, Oxford Vegetarian Study, JACC, JPHC, Takayama), other prospective cohorts have contributed relevant data, usually through individual publications. Overall, the cohorts span multiple continents and reflect a wide range of fermented food exposures.

Among the globally representative studies, the PURE study, spanning 21 countries and more than 136,000 participants, investigated the consumption of dairy products, particularly yogurt, in relation to CVD and all-cause mortality (27). The Golestan cohort study from Iran included more than 42,000 participants and focused on the consumption of dairy products (especially yogurt) and its association with cardiovascular and cancer mortality (28).

In Japan, several long-term population-based cohorts provided extensive data. The Japan Public Health Center-based Prospective Study (JPHC) followed over 93,000 adults for almost two decades and investigated the consumption of fermented dairy and fermented soy products in relation to all-cause, CVD, and cancer mortality (29, 30). The Japan Collaborative Cohort (JACC) study focused on diet and cancer risk in middle-aged adults, including gastric cancer and consumption of fermented soy and fermented dairy products (yogurt, cheese) (31–33). The Takayama study (*n* > 29,000) specifically explored the consumption of bread and fermented soy, particularly natto, in relation to mortality from CVD and stroke (34, 35). The Yamagata study, involving 14,264 adults, investigated the impact of yogurt and milk consumption on all-cause and cancer mortality (36). The Miyagi cohort of more than 11,000 participants examined dietary patterns and cancer risk, including consumption of fermented dairy products (37). A Japanese cohort of Hokkaido residents (*n* = 3,158) was followed from 1984 to 2002 to investigate associations between 37 dietary factors, including miso soup and site-specific cancer mortality (38).

In Europe, the EPIC (European Prospective Investigation into Cancer and Nutrition) project provided data from several national cohorts. The EPIC-NL and EPIC-Italy cohorts investigated the consumption of fermented dairy products in relation to all-cause and cancer mortality in >34,000 and >45,000 participants, respectively (39–41). The MONICA cohort, part of the WHO cardiovascular monitoring initiative, contributed data from Denmark and northern Sweden. In Denmark, 1,746 people were followed for 30 years to investigate the relationship between consumption of fermented dairy products and mortality (42, 43), while in northern Sweden, the MONICA cohort was merged with the Västerbotten Intervention Programme to form the NSHDS cohort, which included more than 103,000 participants and investigated the association between fermented milk and cheese consumption and all-cause mortality (44, 45). The Oxford Vegetarian Study (*n* > 10,000) focused on health-conscious British individuals and their risk of ischemic heart disease (IHD) based on dietary patterns, including a low intake of dairy products (46). The Swedish Malmö Diet and Cancer Study (MDCS) followed over 20,000 participants over a 21-years period and analyzed the effects of fermented versus non-fermented milk consumption in relation to CVD, with consideration of genetic lactase persistence (47). Other Dutch cohorts included the Netherlands Cohort Study (NLCS) (*n* > 120,000), which investigated milk fat consumption and cause-specific mortality (48), the Rotterdam Study, which associated high-fat dairy products with a lower risk of fatal stroke (49), the Hoorn study on high-fat dairy products and CVD mortality (50), and the Zutphen Elderly Study, which investigated the relationship between cocoa consumption, blood pressure, and CVD mortality (51). The Whitehall II study investigated the consumption of fermented dairy products in more than 5,000 British civil servants in relation to cardiometabolic outcomes (52).

In the United States, the Nurses’ Health Study (NHS and NHS II) and the Health Professionals Follow-Up Study (HPFS) provided data from more than 217,000 participants with dietary records spanning more than three decades. These cohorts investigated associations between dairy subtypes and all-cause and CVD mortality (53). The Lutheran Brotherhood Study followed 17,633 men in the US Midwest and examined lung cancer risk in relation to dietary and lifestyle factors (54). The National Health and Nutrition Examination Survey (NHANES) included more than 32,000 participants and examined the association between yogurt and probiotic supplement consumption and all-cause mortality (55). The Million Veteran Programme, involving nearly 190,000 US military veterans, investigated the association between chocolate consumption and the risk of coronary heart disease (CHD) (56). A cohort of Californian Seventh-day Adventists (*n* = 27,529) was followed from 1960 to 1980 to investigate the association between the consumption of foods of animal origin, including cheese, and cause-specific mortality, including heart disease, diabetes, and various cancers (57).

### 3.2 Quality and bias of the human studies

To assess bias, we used the NOS scale for observational studies, which assesses the “selection,” “comparability,” and “outcome” of studies. The quality score of our studies generally ranged from 7 to a maximum of 9, indicating a low risk of bias and reflecting strong methodological rigor in terms of participant selection, control of confounding factors, and outcome measurement (Supplementary Tables 2–5). A smaller number of studies were rated 5–6, indicating a moderate risk of bias, often due to limited adjustment for confounders or shorter follow-up periods. The overall quality of these studies is relatively high and provides a reasonable level of confidence in the results of the different categories of fermented foods in relation to mortality. Despite the fact that some evidence is available, the data we have are still not sufficient and additional high-quality, population-based studies with detailed dietary assessment are needed to clarify these associations and elucidate the underlying mechanisms.

To narratively synthesize the available evidence, we systematically evaluated individual studies reporting associations between specific fermented foods and mortality outcomes. Each association was then summarized using standardized indicative directional labels (“reduce,” “slightly reduce,” “neutral”) based on predefined criteria. In addition to the direction of association, Table 1 also provides an overview of the strength of evidence and the average NOS score for each fermented food category. These summary classifications serve as a comparative overview and are referenced throughout the following section.

**TABLE 1:** Summary of the association between consumption of fermented foods and mortality: indicative direction of effect and strength of evidence.

Ferm. foods	Microbial viability at intake	Mortality outcome	Indicative direct. of effect	Strength of evidence	Avg. NOS score	No. of studies	Total sample size	Follow-up (yrs)	Mean HR	Avg. CI width	Imprecision	Region	Ref.
Total ferm. foods	Variable	All-cause	↔ Neutral	Very low	8	1	34409	15	1.00	0.25	Not serious	**Europe:** Netherlands	(40)
		CVD	↔ Neutral	Very low	8	1	34409	15	1.04	0.47	Not serious	**Europe:** Netherlands	(40)
		Total cancer	↔ Neutral	Very low	8	1	34409	15	1.02	0.35	Not serious	**Europe:** Netherlands	(40)
Ferm. dairy	Yes	All-cause	↓ Slightly reduce	Moderate	7.75	4	10869	12–30	0.92	0.37	Not serious	**Europe:** UK, Denmark, Finland, Netherlands	(43, 50, 52, 59)
		CVD	↔ Neutral	Moderate	8	3	12862	8–17	1.03	1.10	Moderate	**Europe:** Netherlands **South America:** Brazil	(40, 49, 50, 58)
Ferm. milks	Yes	All-cause	↓ Slightly reduce	High	8.17	18	1172824	6–32	0.94	0.27	Not serious	**Africa:** South Africa, Tanzania, Zimbabwe **Asia:** Bangladesh, China, India, Iran, Japan, Malaysia, Pakistan, Philippines **Europe:** Denmark, France, Germany, Greece, Italy, Netherlands, Norway, Poland, Spain, Sweden, Turkey, UK **Middle East:** Palestine, Saudi Arabia, United Arab Emirates **North America:** Canada, USA **South America:** Argentina, Brazil, Chile, Colombia **Oceania:** Australia	(27–29, 36, 39–44, 48, 55, 60–65)
		CVD	↓ Slightly reduce	Moderate	8.21	14	674346	6–32	0.90	0.53	Moderate	**Asia:** Iran, Japan **Europe:** Italy, Netherlands, Sweden **North America:** USA **Oceania:** Australia	(28, 29, 36, 40, 41, 47–49, 55, 61–65)
		Total cancer	↓ Slightly reduce	Moderate	7.85	13	539289	6–32	0.95	0.57	Moderate	**Asia:** Iran, Japan **Europe:** Italy, Netherlands **North America:** USA **Oceania:** Australia	(28, 29, 36–38, 40, 41, 55, 61–65)
Yogurt	Yes	All-cause	↓ Slightly reduce	High	8.23	13	828256	6–32	0.94	0.31	Not serious	**Africa:** South Africa, Tanzania, Zimbabwe **Asia:** Bangladesh, China, India, Iran, Japan, Malaysia, Pakistan, Philippines **Europe:** Denmark, France, Germany, Greece, Italy, Netherlands, Norway, Poland, Spain, Sweden, Turkey, UK **Middle East:** Palestine, Saudi Arabia, United Arab Emirates **North America:** Canada, USA **South America:** Argentina, Brazil, Chile, Colombia **Oceania:** Australia	(27, 28, 36, 39–41, 43, 55, 61–65)
		CVD	↓ Slightly reduce	Moderate	8.27	11	439685	6–32	0.90	0.64	Moderate	**Asia:** Iran, Japan **Europe:** Italy, Netherlands **North America:** USA **Oceania:** Australia	(28, 36, 40, 41, 49, 55, 61–65)
		Total cancer	↓ Slightly reduce	Moderate	7.92	12	445979	6–32	0.95	0.62	Moderate	**Asia:** Iran, Japan **Europe:** Italy, Netherlands **North America:** USA **Oceania:** Australia	(28, 36–38, 40, 41, 55, 61–65)
		GI cancer	? Unclear	Low	6.5	4	233380	9–15	0.97	3.48	Very serious	**Asia:** Japan	(32, 33, 37, 38)
		Lung cancer	↓ Slightly reduce	Low	6.67	3	113012	8–15	0.88	2.12	Very serious	**Asia:** Japan	(37, 38, 69)
		Reprod. cancer	? Unclear	Low	7	2	357429	6–13	1.22	2.73	Very serious	**Asia:** Japan **North America:** USA	(66, 67)
Cheese	Yes*	All-cause	↓ Slightly reduce	High	8	20	1158122	5–40	0.98	0.42	Not serious	**Africa:** South Africa, Tanzania, Zimbabwe **Asia:** Bangladesh, China, India, Iran, Japan, Malaysia, Pakistan, Philippines **Europe:** Denmark, Finland, France, Germany, Greece, Italy, Netherlands, Norway, Poland, Spain, Sweden, Turkey, UK **Middle East:** Palestine, Saudi Arabia, United Arab Emirates **North America:** Canada, USA **South America:** Argentina, Brazil, Chile, Colombia **Oceania:** Australia	(27–29, 39–46, 48, 50, 53, 59, 60, 63–65, 68)
		CVD	↔ Neutral	Moderate	7.92	12	635130	6–40	1.04	0.89	Moderate	**Asia:** Iran, Japan **Europe:** Italy, Netherlands, UK **North America:** USA **Oceania:** Australia	(28, 29, 40, 41, 46, 48–50, 53, 63–65)
		Total cancer	↔ Neutral	Moderate	8	9	496208	6–40	1.05	0.53	Moderate	**Asia:** Iran, Japan **Europe:** Italy, Netherlands **North America:** USA **Oceania:** Australia	(28, 29, 38, 40, 41, 53, 63–65)
		GI cancer	? Unclear	Low	7	3	221774	10–15	1.28	3.42	Very serious	**Asia:** Japan	(32, 33, 38)
		Lung cancer	↔ Neutral	Low	7.5	2	101406	8–15	1.03	3.28	Very serious	**Asia:** Japan	(38, 69)
		Reprod. cancer	? Unclear	Low	7.33	3	373619	6–20	1.38	2.60	Very serious	**Asia:** Japan **North America:** USA	(66, 67, 134)
Ferm. soy	Variable	All-cause	↓↓ Reduce	Very low	8	1	92915	15	0.90	0.16	Not serious	**Asia:** Japan	(30)
		CVD	↓ Slightly reduce	Very low	8	1	92915	15	0.86	0.31	Not serious	**Asia:** Japan	(30)
		Total cancer	↓ Slightly reduce	Very low	8	1	92915	15	0.96	0.28	Not serious	**Asia:** Japan	(30)
Miso	Variable	All-cause	↓ Slightly reduce	Very low	8	1	92915	15	0.92	0.16	Not serious	**Asia:** Japan	(30)
		CVD	↓ Slightly reduce	Low	8.5	2	102159	15–24	0.95	0.31	Not serious	**Asia:** Japan	(30, 73)
		Total cancer	↓ Slightly reduce	Low	7.5	2	94439	14–15	0.77	0.51	Moderate	**Asia:** Japan	(30, 38)
		GI cancer	? Unclear	Very low	5.8	5	501476	10–14	1.14	3.17	Very serious	**Asia:** Japan	(31, 33, 38, 71, 72)
Natto	Yes	All-cause	↓ Slightly reduce	Very low	8	1	92915	15	0.89	0.16	Not serious	**Asia:** Japan	(30)
		CVD	↓↓ Reduce	Low	8	2	121994	15–16	0.77	0.26	Not serious	**Asia:** Japan	(30, 34)
		Total cancer	↓ Slightly reduce	Very low	8	1	92915	15	0.93	0.27	Not serious	**Asia:** Japan	(30)
Ferm. vegeta-bles	Variable	All-cause	↓↓ Reduce	Very low	8	1	34409	15	0.88	0.22	Not serious	**Europe:** Netherlands	(40)
		CVD	↔ Neutral	Very low	8	1	34409	15	1.05	0.49	Not serious	**Europe:** Netherlands	(40)
		Total cancer	↓ Slightly reduce	Very low	8	1	34409	15	0.93	0.34	Not serious	**Europe:** Netherlands	(40)
Ferm. meat	Variable	All-cause	↔ Neutral	Very low	8	1	34409	15	1.00	0.23	Not serious	**Europe:** Netherlands	(40)
		CVD	? Unclear	Very low	8	1	34409	15	1.17	0.49	Not serious	**Europe:** Netherlands	(40)
		Total cancer	↔ Neutral	Very low	8	1	34409	15	0.99	0.33	Not serious	**Europe:** Netherlands	(40)
Bread	No	All-cause	↓ Slightly reduce	Low	7.5	2	1121	5–15	0.73	0.96	Moderate	**Europe:** France, Italy	(42, 68)
		CVD	↓ Slightly reduce	Low	8.5	2	82548	14–15	0.88	0.50	Not serious	**Asia:** Singapore, Japan	(35, 74)
		Lung cancer	↓ Slightly reduce	Low	7.5	2	20791	14–20	0.67	1.63	Very serious	**Asia:** Japan **North America:** USA	(38, 54)
Choco& cocoa	No	All-cause	↓ Slightly reduce	Moderate	7.75	4	217335	14–31	0.91	0.11	Not serious	**North America:** USA **Europe:** Finland	(77–80)
		CVD	↓↓ Reduce	Moderate	7.67	6	413579	3–31	0.79	0.24	Not serious	**North America:** USA, **Europe:** UK, Netherlands, Finland	(51, 56, 77, 79–81)

Viability at intake designates expected microbial viability at consumption (Yes/Variable/No; see footnote). Avg. CI width, average confidence interval width; Avg. NOS score, average Newcastle-Ottawa Scale score; direct., direction; Ferm., fermented; Follow-up (yrs), duration of follow-up in years; HR, hazard ratio; Ref., references; Reprod., reproductive. Indicative labels (“reduce,” “slightly reduce,” “neutral”) were defined *a priori* from study-level HR patterns, confidence-interval behavior, and cross-study consistency (see Section “2 Methods” and Supplementary Material). “Slightly reduce” indicates a consistent protective trend despite non-significance in some individual studies. Microbial viability at consumption was assessed based on typical product processing and study-reported preparation: “Yes” – viable cultures expected when consumed; “No” – viability not expected due to baking/roasting/pasteurization; “Variable” – depends on production process and preparation.

*Processed cheeses are typically heat-treated/emulsified and not expected to contain viable microbes.

### 3.3 Methodological rationale for the synthesis

We aimed to integrate all relevant evidence, not only the subset suitable for quantitative pooling, and to provide detailed, category-level findings that are comparable across the fermented foods spectrum. As the density of evidence was uneven across categories, with several represented by only one to two eligible cohort studies (fermented vegetables, fermented meat, fermented soy, and total fermented foods), our narrative SWiM-aligned structured synthesis provided a consistent way to convey the indicative direction and strength of associations while keeping the whole landscape in view (12, 24–26). To contextualize the epidemiological findings, we linked the associations with mechanistic and experimental evidence, including microbial metabolites, food matrix and processing effects in line with EFSA-oriented considerations of biological plausibility and coherence across evidence types. In parallel, we annotated microbial viability at the point of ingestion to reflect prevailing processing and preparation practices, distinguishing categories with expected viable microbial exposure and provide context for interpreting the associations. Taken together, this design provides a transparent, reproducible overview of where the indicative direction of effect is consistent across studies and where uncertainty remains.

### 3.4 Relationship between consumption of the fermented food and mortality risk

#### 3.4.1 Fermented foods

To date, only one cohort study has investigated the association between fermented foods, considered as a unified dietary group, and mortality risk. This study was conducted in the Netherlands as part of the EPIC-NL cohort (40). Findings on total fermented foods consumption and mortality were largely inconclusive (Supplementary Table 2). No significant association was found with all-cause mortality (HR 1.00; 95% CI: 0.88–1.13), CVD mortality (HR 1.04; 95% CI: 0.83–1.30), or cancer mortality (HR 1.02; 95% CI: 0.86–1.21). Therefore, in accordance with our rule-based synthesis, we classified these associations as “neutral” (Table 1). The study controlled for a wide range of dietary and lifestyle confounders, which increases the robustness of the results. However, due to the lack of supporting studies in other populations, potential variations in fermentation processes across cultures, and differences in the level of food processing compared to traditional foods, the findings cannot be generalized beyond this European cohort. In addition, the exposure assessment was based on food frequency questionnaires, which may not have fully captured the diversity of fermented food consumption. Further studies from other geographic regions are needed to draw more definitive conclusions. Given the limitations of examining fermented foods as a broad category, the potential health effects may be more apparent when specific types of fermented foods are considered individually.

#### 3.4.2 Fermented dairy products

The association between the consumption of fermented dairy products and all-cause mortality was evaluated in four European cohort studies involving 10,869 individuals (Supplementary Table 3). Additionally, three studies including 12,862 participants examined the impact of fermented dairy on CVD mortality. Among these, two reported statistically significant reductions in mortality risk: one for all-cause mortality (52) and another for CVD mortality (58). Although the remaining studies suggested a possible reduction in all-cause and CVD mortality, these findings were not statistically significant. Notably, the study reporting a significant effect on all-cause mortality also observed a similar reduction for total dairy consumption (HR 0.72; 95% CI: 0.52–0.99; *p* = 0.04) (52), suggesting that the health benefits may not be solely attributed to fermentation. However, other papers reported neutral associations between total dairy intake and mortality outcomes (43, 49, 50, 59), with one study specifically indicating no effect of non-fermented dairy (59). While statistical significance was only reported in a few studies, the consistent trend toward reduction in all-cause mortality supports the “slightly reduce” label (Table 1 and Supplementary Methods), according to predefined criteria integrating directionality and HR consistency. For CVD mortality, the findings were more heterogeneous and support a “neutral” classification.

Based on the current literature, no definitive conclusions can be drawn regarding the overall effect of fermented dairy products on all-cause or cause-specific mortality. The heterogeneity of these products and the limited number of studies that consider fermented dairy as a broad category constrain the strength of conclusions. Most available studies evaluated individual products such as cheese, fermented milk or yogurt, rather than the group as a whole. Additionally, research addressing the category of fermented dairy across a wider geographical range remains sparse, which limits the generalizability of findings.

#### 3.4.3 Fermented milk products

Among fermented dairy products, fermented milk, including yogurt, sour milk, quark, and probiotic drinks such as Yakult, was more extensively represented in the literature (Supplementary Table 3). Eighteen papers with low risk of bias, involving 1,172,824 participants, were included in the analysis of all-cause mortality. Of these, 10 papers reported a statistically significant reduction in mortality (27–29, 36, 48, 55, 60, 61), including reported estimates such as HR 0.70; 95% CI: 0.49–0.99 (36), HR 0.83; 95% CI: 0.69–0.99 (27), and HR 0.89; 95% CI: 0.89–1.00 (28) with populations across every continent. Non-significant reduction trends (40–42, 44, 49, 62–64) and neutral associations (39, 43, 65) were also observed across several regions, suggesting that the inconsistencies may be largely due to heterogeneity in product formulation and/or degree of processing, particularly the addition of sugar and other sweeteners, and/or degree of processing. Interestingly, Goldbohm et al. reported no difference in all-cause mortality between consumers of high-fat and low-fat fermented milk (48). Given the high quality of the evidence, the large number of studies and the extensive participant pool, there is sufficient evidence that indicate a modest but consistent reduction in all-cause mortality associated with consumption of fermented milk. This is reflected as a “slightly reduce” classification in the summary table (Table 1), according to our rule-based synthesis integrating the direction of HRs across studies.

For CVD mortality, 14 papers with a combined sample of 674,346 participants were considered. In five papers (28, 29, 47, 48, 61), statistically significant reductions were observed, including particularly strong effects such as HR 0.64; 95% CI: 0.46–0.90 for males (61), HR 0.77; 95% CI: 0.64–0.92 for females (48) and HR 0.74; 95% CI: 0.61–0.91 for males and females (28). Other studies reported either non-significant reductions (40, 41, 49, 55, 63–65) or neutral effects. There was no clear regional pattern. Given the overall moderate quality of the evidence (based on our criteria integrating magnitude, consistency, precision, NOS) and the consistent downward trend observed in several studies, there is moderately strong support for an inverse association between fermented milk consumption and CVD mortality. This pattern of results is reflected in the evidence synthesis (Table 1) as a “slightly reduce” classification, indicating a consistent protective direction of effect across cohorts, even in the absence of uniform statistical significance.

For cancer mortality, 13 papers covering 539,289 participants across different continents were included. Statistically significant reductions were found in some studies (29, 36, 62), with estimates such as HR 0.53; 95% CI: 0.27–0.99 (36) and HR 0.87; 95% CI: 0.78–0.98 (62). Others reported slight but non-significant reductions (28, 38, 61) or neutral effects (37, 40, 41, 49, 55, 63, 64). The quality of evidence for all-cancer mortality was suggested as moderate according to our criteria, and although most individual results did not reach statistical significance, the consistent trend toward a slight reduction across studies suggests a beneficial effect that warrants further investigation. Accordingly, the indicative direction of effect is summarized as “slightly reduce” (Table 1), reflecting a modest but consistent protective trend.

#### 3.4.4 Yogurt

As yogurt was the predominant component of fermented milk consumption, it was analyzed separately to assess its specific impact on all-cause, CVD, and cancer mortality. Thirteen studies examined yogurt consumption and all-cause mortality across populations on every continent. Of these, five papers reported a statistically significant reduction in mortality (27, 28, 36, 55, 61), indicating a consistent beneficial association across diverse populations. A further five papers showed a slight but non-significant reduction in all-cause mortality (40, 41, 62–64), while the remaining three papers reported neutral effects (39, 43, 65). Given the number of studies, the large total sample size (828,256), the generally high quality of evidence, and the directional consistency with acceptable CI-based precision, yogurt consumption is associated with a moderate reduction in all-cause mortality. In line with our rule-based synthesis (see Supplementary Methods), this pattern is consistently observed across studies and summarized (Table 1) as a “slightly reduce” classification, based on the overall direction of HRs and their precision.

A total of eleven papers (439,685 participants) evaluated the association between yogurt consumption and CVD mortality. Two papers, one from Iran and another from Japan, reported statistically significant reductions, with the effects being particularly pronounced in female participants (28, 61). A slight, non-significant reduction was observed in several other studies, indicating a consistent trend. These include findings from Europe, the USA and Japan (40, 41, 49, 55, 62–65). Despite generally small and mostly non-significant effect sizes, the moderate quality of evidence according to our criteria suggest the interpretation that yogurt consumption may help reduce CVD mortality. Due to the consistency of this trend across different studies and populations, the association is also classified as “slightly reduce” (Table 1).

Yogurt consumption and its impact on all-cancer mortality have also been evaluated in several studies. Two papers from the USA and Japan (36, 62) showed statistically significant reductions. In addition, three papers reported slight, non-significant reductions (28, 38, 61), while the findings of the remaining papers showed neutral effects (37, 40, 41, 55, 63, 64). Based on the moderate-quality of evidence suggested by our criteria and the consistent indicative direction of effects reported in 12 studies (445,979 participants), there is modest support for a potential inverse association between yogurt consumption and cancer mortality. This consistent trend is reflected with a “slightly reduce” classification (Table 1).

The evidence on yogurt consumption and site-specific cancer outcomes remains limited and inconsistent across cancer types. We specifically examined four papers from Japan that focused on yogurt consumption and GI cancers and included 233,380 participants. These studies (32, 33, 37, 38) varied in their conclusions: while some indicated a slight reduction in the risk of GI cancer, the results were not statistically significant and the quality of the studies was limited due to regional scope and potential bias. Findings related to lung cancer mortality showed a consistent trend toward a slight reduction; however, none of the associations reached statistical significance, and the overall evidence base was relatively weak. As for reproductive cancers, the evidence was also scarce, with only two relevant studies: one on ovarian cancer in Japan (66), and one on prostate cancer in the USA (67). For lung cancer mortality, a “slightly reduce” classification was applied in Table 1, indicating an inverse trend. No directional classification was assigned for other site-specific cancers, given the lack of sufficient or consistent evidence.

#### 3.4.5 Cheese

Of the fermented dairy products, cheese has been studied most extensively in relation to mortality (Supplementary Table 3). A total of 20 papers with 1,158,122 participants across every continent investigated the association between cheese consumption and all-cause mortality. Six papers reported statistically significant reductions (28, 44, 53, 60, 63, 64), with reported HRs such as 0.83; 95% CI: 0.72–0.95 (60), 0.84; 95% CI: 0.73–0.96 (28) and 0.94; 95% CI: 0.91–0.97 (44). The findings of ten other papers showed a slight, non-significant reduction in mortality (27, 29, 39, 41, 45, 48, 50, 59, 65), while four papers reported neutral effects (40, 42, 46, 68). Supported by a high strength of evidence, a consistent indicative direction of effect, acceptable CI-based precision, generally good study quality, and drawing on from over 1.15 million individuals, there is sufficient basis for a modest association between cheese consumption and reduced all-cause mortality. In line with our rule-based synthesis, this relationship is classified as “slightly reduce” (Table 1), reflecting a consistent protective trend observed across large and diverse cohorts, even in cases where statistical significance was not uniformly achieved.

The effects of cheese consumption on CVD mortality were less clear. Of the 12 papers, three (conducted in the USA, Iran, and Japan) showed a statistically significant reduction in CVD mortality, with the effect being more pronounced in males (28, 29, 53). Specifically, studies reported HR 0.74; 95% CI: 0.61–0.91 for males and females (28), HR 0.86; 95% CI: 0.75–0.99 for males (53), and HR 0.87; 95% CI: 0.78–0.97 for males (29). The findings of five papers from Japan, Europe, and Australia showed slight but non-significant reductions (40, 41, 48, 63, 65), while the other studies reported neutral results. A study from the UK with a high risk of bias (46) observed a potential trend toward increased mortality from IHD with higher cheese consumption. The findings across studies are inconsistent, and no clear beneficial or harmful association can be established. Overall, the evidence is of moderate quality and suggests a neutral effect of cheese consumption on CVD mortality. Given the variability and mixed nature of the results, the association is classified as “neutral” (Table 1), indicating no clear overall direction.

In terms of all-cancer mortality, nine papers analyzed the effects of cheese consumption with a combined sample of 496,208 participants. Four of these papers reported slight, non-significant reductions (28, 53, 63, 64), while the remaining papers found neutral associations. Given the moderate strength of evidence and the predominance of weak or non-significant associations, current findings suggest no clear link between cheese consumption and overall cancer mortality. Accordingly, this outcome was classified as “neutral” in the summary table (Table 1).

When considering specific types of cancer, the findings were similarly inconclusive. For GI cancers, three papers from Japan with 221,774 participants reported neutral associations with a wide range of confidence intervals (32, 33, 38). Due to the low quality of the evidence and the very high imprecision, no reliable conclusions can be drawn regarding cheese consumption and GI cancer mortality. Two Japanese papers analyzed lung cancer outcomes in a total of 101,406 participants. One paper reported a neutral effect, while the other indicated a reduction in lung cancer mortality in males (38, 69). Despite of the suggested benefit in males, the evidence is of low quality and imprecise, so it is not sufficient to confirm an association. Evidence was also limited for reproductive cancers. Three papers (373,619 participants) from Japan and the USA were available. Due to the low strength of evidence and serious limitations in study design and regional coverage, no firm conclusions can be drawn regarding the association between cheese consumption and reproductive cancer mortality. Accordingly, site-specific classifications in Table 1 were marked as “unclear” for GI and reproductive cancers due to inconsistent findings and limited evidence, while lung cancer mortality was categorized as “neutral.”

Overall, the quality of evidence for all-cause mortality and CVD mortality was generally higher than for cancer-specific mortality. The limited number of high-quality, geographically diverse studies addressing specific cancers continues to limit interpretability in these subdomains. The evidence for cheese and all-cause mortality is relatively strong, while the evidence for CVD and cancer mortality is weaker and more ambiguous. The discrepancies in the results may be due to regional differences in cheese types, preparation methods, and dietary context. In Europe, cheese is often artisanal, contains less sodium, and is consumed in moderation. In contrast, in the USA, highly processed cheeses with higher fat and sodium content are more prevalent and are often consumed with energy-dense foods (70). On the other hand, in many Asian countries, including Japan, cheese consumption has traditionally been low but is gradually increasing, often involving processed or Western-style cheeses, which may still differ nutritionally and culturally from traditional dairy patterns seen in Western populations.

#### 3.4.6 Fermented soy products

We identified seven studies that addressed the association between consumption of fermented soy products, miso and natto, and mortality, in terms of all-cause, CVD, and cancer mortality (Supplementary Table 4). These studies were predominantly conducted in Japan. Collectively, they included 632,714 participants, with follow-up ranging from 10 to over 20 years. Only one high-quality prospective cohort study explicitly investigated total consumption of fermented soy products and reported a statistically significant inverse association with all-cause mortality (males: HR 0.90, 95% CI 0.83–0.97, *p* = 0.05; females: HR 0.89, 95% CI 0.80–0.98, *p* = 0.01) and CVD mortality in males (HR 0.82, 95% CI 0.7–0.97; *p* = 0.04) (30). Less significant correlations were found for cancer mortality. Remarkably, this study also found no benefit for non-fermented soy products, indicating the potential additional benefit of fermentation. Although several studies have examined specific fermented soy products, such as miso and natto, their limited number and narrow regional focus constrain the generalizability of the findings. Based on consistent inverse trends, associations with all-cause mortality were classified as “reduce,” while those for CVD and total cancer were rated as “slightly reduce” (Table 1).

#### 3.4.7 Miso

Miso was investigated in seven studies with sample sizes ranging from 1,500 to over 90,000 participants (30, 31, 33, 38, 71–73). The health outcomes examined included all-cause, CVD, and all-cancer mortality as well as GI cancer mortality. A significant reduction in all-cause mortality was observed in females consuming miso (HR 0.89, 95% CI 0.81–0.97; *p* = 0.03) (30). Although some other studies indicated a slight protective trend for some GI cancers, most of these were not statistically significant reductions (31, 38, 71). In addition, consumption levels and accompanying dietary patterns varied, making it difficult to isolate the effects of miso itself. Limitations include the lack of geographic diversity and the possible influence of other dietary or lifestyle factors typical of the Japanese population. To summarize, although miso is commonly consumed as a fermented food in Japan, current evidence does not show its clear association with mortality risk. Nonetheless, based on our synthesis rules and modest yet directionally consistent patterns in some subgroups, miso consumption was classified as “slightly reduce” for all-cause, CVD, and overall cancer mortality, while the indicative direction of effect for GI cancer remained “unclear” due to limited evidence (Table 1).

#### 3.4.8 Natto

Only two studies specifically investigated natto consumption and mortality outcomes (30, 34). Both were conducted in Japan, with a combined sample size of more than 120,000 participants. Katagiri et al. found that higher natto consumption was significantly associated with reduced all-cause mortality in females (HR 0.84, 95% CI 0.76–0.93; *p* = 0.001) and lower CVD mortality in males and females (males: HR 0.76, 95% CI 0.65–0.9, *p* = 0.002; females: HR 0.79, 95% CI 0.65–0.95, *p* = 0.01), although there was no association with cancer mortality (30). The consistency of the inverse associations between the studies, despite the small geographical range, suggests a potential beneficial role of natto, particularly for cardiovascular health. However, generalization beyond the Japanese population is limited, and dietary habits and preparation methods could influence the results. Despite limited studies, the strong consistency in reported HRs supports the “reduce” or “slightly reduce” classification (Table 1).

#### 3.4.9 Fermented vegetables

Fermented vegetables have been less extensively studied compared to other fermented food categories (Table 1 and Supplementary Table 2). Only the EPIC-NL study (40) has examined their association with all-cause, CVD, and cancer mortality. In this Dutch cohort, fermented vegetable consumption was associated with reduced all-cause mortality (HR 0.88; 95% CI: 0.78–1.00; *p* = 0.034). While associations with CVD and cancer mortality were not statistically significant, a trend toward reduced cancer mortality was observed. The study was well conducted and adjusted for a wide range of confounders; however, its findings may not be generalizable beyond this study population. The types of fermented vegetables commonly consumed in the Netherlands, such as sauerkraut, may differ from those elsewhere in microbial content, fermentation processes, and salt concentrations, which could influence health outcomes; whereas, many pickles are vinegar-acidified rather than fermented. Additional studies, particularly from non-European populations and with more detailed exposure assessments, are needed to clarify the health effects of fermented vegetables in diverse dietary contexts. Although evidence comes from a single cohort, the observed indicative direction of effect and confidence-interval pattern meet our specified criteria for the classification as “reduce” for all-cause mortality, “slightly reduce” for overall cancer mortality, and “neutral” for CVD mortality (Table 1).

#### 3.4.10 Fermented meat

Fermented meat has likewise been investigated infrequently, with the EPIC-NL study (40) being the only cohort examining its relationship with mortality outcomes. In this analysis, fermented meat consumption showed a neutral association with all-cause mortality (HR 1.00; 95% CI: 0.89–1.12; *p* = 0.9), and similar findings were reported for cancer mortality. In the case of CVD mortality, the association pointed toward an adverse direction. As noted by the authors of the study, the intake of fermented meat in the cohort was relatively low, and potential misclassification due to limitations in the dietary assessment tool may have attenuated the observed associations. Consequently, despite the suggestive trend for CVD, the effect was ultimately deemed unclear due to the lack of statistical significance and absence of supporting evidence from other cohorts. As with fermented vegetables, the specific types of fermented meat consumed in the Netherlands (e.g., salami, cured sausages) may differ considerably from those in other cultures, in terms of raw material composition, microbial profiles, and processing methods. Further research in other populations and dietary contexts is warranted to determine whether these findings are consistent across different fermented meat products and consumption patterns. Overall, these patterns are consistent with the indicative classifications in Table 1: “neutral” for all-cause and total cancer mortality, and “unclear” for CVD mortality.

#### 3.4.11 Bread

Bread consumption and its association with mortality was investigated in six cohort studies involving a total population of approximately 104,460 participants in Europe, Asia, and North America (35, 38, 42, 54, 68, 74). The outcomes measured included all-cause, CVD, and lung cancer mortality (Table 1 and Supplementary Table 5). Although the trend showed a slight, non-significant reduction in mean HRs for mortality risk, the confidence intervals were generally wide, limiting the reliability of the findings. The heterogeneity of the results could be due to differences in the type of bread (e.g., whole grain vs. refined bread, yeast vs. sourdough bread) and other eating habits. Beneficial associations are more likely to be expressed in regions where bread is consumed as part of a traditional, minimally processed diet, while neutral effects are more common in studies where refined or industrial bread products are more commonly consumed (75, 76). Although most studies reported non-significant results, the consistent indicative direction of effect led to a “slightly reduce” classification in Table 1, based on predefined criteria.

#### 3.4.12 Chocolate and cocoa

A total of seven cohort studies investigated the association between the consumption of chocolate or cocoa and mortality outcomes (Table 1 and Supplementary Table 5). These studies, conducted in North America and Europe, included a total of 427,203 participants and featured long-term follow-up periods of up to 31 years. All-cause mortality was examined in four studies (217,335 participants) (77–80), and three of these studies reported a statistically significant inverse association with chocolate consumption (78–80). For instance, a large Finnish cohort showed a 9%–17% reduced risk of all-cause mortality (80), with an HR of 0.88 (95% CI: 0.85–0.92). CVD mortality was investigated in six studies involving 413,579 individuals (51, 56, 77, 79–81). Of these, five studies reported a statistically significant reduction in CVD mortality with higher chocolate or cocoa intake (51, 56, 79–81). It is noteworthy that the HRs in the different cohorts ranged from 0.50 to 0.92, with confidence intervals below 1.0, indicating robust protective effects. Specific cause-related outcomes, such as cancer mortality, were less frequently addressed but also showed a trend toward a slight reduction in mortality risk (77, 79, 80).

While the findings suggest a consistent inverse association between chocolate consumption and CVD mortality and a possible modest reduction in all-cause mortality, several limitations must be noted. The composition of the chocolate products (e.g., dark vs. milk chocolate, added sugar content) was not consistently described in the studies, which could affect comparability. In addition, most of the studies were carried out in high-income, Western populations, which limits the generalizability to other regions. In summary, moderate evidence suggests a slight to moderate inverse association between chocolate or cocoa consumption and both all-cause and CVD mortality. While effect sizes and statistical significance varied, the consistent direction of findings supports a “reduce” classification for CVD mortality and a “slightly reduce” classification for all-cause mortality, as shown in Table 1.

### 3.5 Mechanistic insights and biological plausibility behind the observed effects of fermented foods: matrix composition, microbial function, and health risk-benefit evaluation

To align the mechanisms with exposure, we attempted to interpret and link the results according to microbial viability at the point of consumption (Table 1). Foods typically consumed with live microbes, such as yogurt/fermented milks (live starter cultures) and natto (consumed unheated), may plausibly act via direct microbial functions; some traditionally unpasteurized fermented vegetables also contain viable lactic acid bacteria. In cases where viability is absent due to processing, biological effects may still arise from fermentation-derived metabolites and matrix changes, for example, sourdough and other breads (microbes inactivated by baking), chocolate/cocoa (roasting/conching), and miso when consumed primarily as a hot soup. Several categories are context-dependent: many ripened cheeses retain viable microorganisms, whereas processed cheeses generally do not; fermented vegetables and fermented meats may or may not contain viable cells depending on pasteurization, curing/drying, water activity, pH, and storage. Crucially, even without viable microbes, fermentation alters the food matrix and produces organic acids, bioactive peptides, exopolysaccharides, and transformed polyphenols that can affect gut ecology, glycemic responses, mineral bioavailability, inflammation, and cardiometabolic risk – making the observed associations biologically plausible (6, 7, 10, 82).

#### 3.5.1 Fermented foods

The health effects of fermented foods are mediated by several interlinked and biologically plausible mechanisms. Central to this process is the synthesis and enhanced bioavailability of bioactive compounds, including peptides, polyphenols, vitamins, and microbial metabolites such as short-chain fatty acids (SCFAs) (8). Notably, certain fermented foods, like bread, may contain dietary fibers that serve as substrate for microbial fermentation and thus promote the production of SCFAs. The bioactive compounds exert beneficial effects on key physiological processes by modulating systemic inflammation, oxidative stress, blood pressure, lipid profiles, glycemic control, and immune function (6). A distinguishing feature of fermented foods is also their ability to positively influence the gut microbiota (7, 83). Whether through the direct introduction of living microorganisms or the stimulation of endogenous microbial diversity and activity, fermented foods promote gut health and host-microbe interactions (6–8, 83).

From an EFSA standpoint, health claims must be substantiated not only by mechanistic plausibility, but also by clearly defined food characteristics and consistent evidence of a cause-and-effect relationship. Fermented foods are highly heterogeneous in their composition and function, depending on the food matrix, the microbial strains involved, the duration of fermentation, and whether the product is traditionally or industrially processed. For example, artisanal fermentation often leads to greater microbial diversity and a higher content of bioactive compounds than commercially produced equivalents (6). The lack of high-quality randomized controlled trials (RCTs) limits the ability to draw firm conclusions about the effects of fermented foods, particularly regarding dose-response relationships, population-specific outcomes, and contextual risks. Regulatory bodies such as EFSA require such trials to demonstrate efficacy, establish intake thresholds, and account for inter-individual variability, including differences in genetics, microbiome profiles, and dietary habits. While observational studies suggest that consumption of fermented foods is associated with lower all-cause and cause-specific mortality (40), RCTs investigating this relationship remain scarce. Conducting long-term RCTs with mortality as the primary endpoint is generally not feasible due to the extensive duration, large sample sizes, and ethical considerations required. As a more practical alternative, well-designed RCTs can focus on intermediate clinical outcomes that are strongly associated with mortality risk, such as blood pressure, lipid profiles [low-density lipoprotein (LDL) cholesterol and high-density lipoprotein (HDL) cholesterol], systemic inflammation, and muscle mass. These intermediate endpoints provide a feasible means to elucidate causal mechanisms and assess the impact of fermented food consumption on key physiological processes associated with mortality. In parallel, observational studies remain crucial to establish long-term associations between fermented food consumption and mortality risk in real-world settings, thereby complementing the mechanistic insights gained from RCTs. This dual approach is also aligned with the broader objectives of the PIMENTO initiative, where other research groups have been investigating the health effects of fermented foods in detail across a range of clinical and biological endpoints.

It is important to note that not all fermented foods convey equal health benefits. There is relatively strong epidemiological and mechanistic evidence for fermented dairy and fermented soy products, which are sources of viable microbes, peptides, and polyphenols. In contrast, fermented meats, which are often high in sodium and nitrites, might show an adverse association with mortality despite their microbial fermentation (84). In addition, fermentation can in some cases lead to high levels of biogenic amines (e.g., histamine), which can potentially trigger adverse reactions (85). Given this variability, food-specific analyses are essential to accurately assess health effects. The following sections therefore explore different categories of fermented foods in more detail, focusing on their distinct matrix characteristics, microbial ecology, and evidence for health effects.

#### 3.5.2 Fermented dairy

In terms of mortality risk, our findings revealed no substantial differences in HRs between total dairy and fermented dairy products, with both categories showing neutral or beneficial associations. This observation is in line with results from previous meta-analyses. For example, Guo et al. reported that total, full-fat, and fermented dairy products were either neutrally or inversely associated with all-cause and CVD mortality, with no consistent benefit observed for low-fat dairy products over full-fat dairy products (86). These results suggest that the health impact of saturated fat in dairy products is determined more by the structure and composition of the food matrix than by fat content alone. Recent evidence indicates that the food matrix may influence the absorption, metabolism, and physiological effects of saturated fats, particularly in dairy, potentially attenuating their impact on blood lipids and cardiovascular risk (87). Other cause-specific mortality findings add further nuance. Meta-analysis from Jin et al. found a 29% reduction in colorectal cancer mortality associated with higher total dairy consumption, possibly due to the protective effects of calcium and fermentation-derived bioactive compounds (88). Conversely, Zhao et al. observed a modest increase in prostate cancer risk associated with high total dairy consumption, whereas yogurt showed no significant association with prostate cancer mortality (89). When assessed individually, fermented dairy products appear to confer a stronger protective effect. Our findings are consistent with published meta-analyses, which consistently found an association between yogurt consumption and lower mortality from all-cause, CVD, and cancer (86, 88, 90–92). Gao et al. reported that 200 g/day of yogurt was associated with significantly reduced all-cause mortality (HR = 0.88; 95% CI: 0.80–0.96) and CVD mortality (HR = 0.87; 95% CI: 0.77–0.99) (90). Similarly, Hu et al. reported a 20% lower risk of stroke associated with consumption of fermented milk (93).

Fermented dairy products differ significantly from their non-fermented counterparts in terms of nutrient profile, acidity, structure, and microbial composition. Their health impacts are modulated by the characteristics of the dairy matrix, which includes fat-protein interactions, the bioavailability of minerals, and the presence of microbial and enzymatic fermentation products. Common products examined in the cohorts reviewed include yogurt, cheese, sour milk, fermented cream, quark, and probiotic drinks such as Yakult. Fermented milk, in this context, refers to milk that has been fermented by specific microorganisms that can lower the pH and lead to coagulation (94). Yogurt is produced with *Streptococcus thermophilus* and *Lactobacillus delbrueckii* subsp. bulgaricus (95), while mesophilic microorganisms such as *Lactococcus lactis* and *Leuconostoc* spp. are typically used in products like quark and fermented cream (96). Yakult, a commercially available probiotic drink, is fermented with *Lacticaseibacillus paracasei* Shirota (97). Cheese, another important fermented dairy product, is produced by coagulation, typically with rennet, and whey removal, followed by ripening (98). The ripening process drives microbial and enzymatic transformations that improve flavor, texture, peptide concentration, and nutrient bioavailability. Common bacterial cultures used in cheese production include *Lactococcus lactis*, *Streptococcus thermophilus*, *Lactobacillus helveticus*, and *Propionibacterium freudenreichii*, among others, each contributing to specific sensory and nutritional properties depending on the cheese type (8). Despite its generally higher sodium and fat content, cheese contains fermentation-derived compounds, a favorable calcium-to-phosphorus ratio and vitamin K2 factors that may explain its occasionally observed protective properties. Hu et al. conducted a Mendelian randomization analysis linking these mechanisms to cheese consumption and a reduced risk of type 2 diabetes, heart failure, CHD, and ischemic stroke (99). In addition, fermentation also contributes to the formation of beneficial fatty acids such as conjugated linoleic acid (CLA), particularly the cis-9, trans-11 isomer, as well as trans-vaccenic acid and cis-palmitoleic acid. These compounds are most commonly found in fermented full-fat products such as cheese and fermented cream and are associated with improved lipid metabolism, better vascular health, and reduced systemic inflammation (58, 63, 100, 101). In yogurt and cheese, fermentation also promotes the formation of bioactive peptides that inhibit angiotensin-converting enzyme (ACE), leading to improved vasodilation and a reduction in blood pressure, a mechanism that supports an inverse association with stroke (28, 40, 49, 53). In addition, fermented dairy products improve vitamin D-mediated absorption of calcium and phosphorus (102). Another microbial metabolite, hippurate, has been detected in higher concentrations in urine following the consumption of yogurt and fermented milk, which is due in particular to the activity of the yogurt starter culture (103). Higher levels of urinary hippurate have been linked to increased gut microbiota diversity, a feature commonly associated with better metabolic health (104). It is hypothesized that these microbial and biochemical effects explain the observed inverse association between the consumption of fermented dairy products and all-cause as well as cause-specific mortality (27, 36, 62). On the other hand, salt content, especially in cheese, remains a potential concern for salt-sensitive populations (27).

In summary, the health impacts of consuming fermented dairy products depend on numerous interacting factors, including fermentation status, matrix complexity, nutrient composition, and microbial content. Future dietary recommendations should move away from simplistic fat-based categorizations and instead adopt a matrix-based, product-specific framework that considers both the degree of processing and the diverse health effects of different fermented dairy types.

#### 3.5.3 Fermented soy

A growing body of epidemiological and mechanistic research underscores the distinct health benefits of fermented soy foods compared to their non-fermented counterparts. Our analysis confirms that regular consumption of fermented soy products, particularly natto, is consistently associated with reduced all-cause and CVD mortality (30). In contrast, non-fermented soy products generally demonstrate weaker or neutral associations with health outcomes, suggesting that fermentation plays a crucial role in enhancing soy’s functional properties. The health effects also appear to differ between the fermented soy products, with natto showing more robust associations than miso. The findings of other meta-analyses further support this distinction. Namazi et al. reported that higher total soy consumption was significantly associated with lower all-cause mortality (105). In particular, fermented soy products such as miso and natto were inversely associated with CVD mortality, largely due to the fermentation-enhanced bioactive compounds such as isoflavones and natto-kinase. These effects were most pronounced in females and in individuals with a higher intake of soy protein and isoflavones. Other meta-analyses suggest that daily intakes of 25–60 g of soy products are associated with reduced cancer incidence and mortality. For example, each 25 g/day increase in soy intake lowers cancer incidence by 4% (106), 54 g/day reduces cancer risk by 11% (107), and a 10 mg/day increase in soy isoflavones is associated with a 7%–9% reduction in cancer mortality (108).

The health benefits of fermented soy are deeply rooted in its nutritional matrix. Fermented soy foods such as natto, miso, tempeh, and soy sauce are rich in plant protein, dietary fiber, polyunsaturated fatty acids (PUFAs), isoflavones, and microbial exopolysaccharides (109). The fermentation process, which is driven by microbial species such as *Bacillus* (*B.*) *subtilis* (natto) and *Aspergillus oryzae* (miso), converts these nutrients into more bioavailable and bioactive forms. Glycosylated isoflavones are converted into aglycones, which have stronger antioxidant, anti-inflammatory, and estrogenic activity (30). In addition, fermentation produces unique compounds such as natto-kinase and polyamines (e.g., spermidine), which offer additional cardioprotective benefits. Natto-kinase, a serine protease produced by *B. subtilis* during natto fermentation, exhibits strong fibrinolytic activity, reported to be four times stronger than that of plasmin. It promotes clot degradation, improves blood circulation and lowers blood pressure, as shown in both preclinical and human studies (34, 110, 111). In addition, polyamines such as spermidine are associated with reduced oxidative stress and improved cellular resilience, with epidemiological evidence linking higher spermidine intake to lower heart failure mortality rates (30).

Importantly, the microbial diversity promoted during fermentation also contributes to health outcomes. According to the comprehensive review on fermented foods and gut health (6), the variety of microbial species present in fermented soy products can increase gut microbial diversity, which is a key determinant of systemic inflammation, metabolic regulation, and immune function. Fermented soy foods provide both live microorganisms and microbial metabolites that interact with the host system. However, not all fermented soy products offer the same benefits. Miso, for example, is rich in fiber, potassium and isoflavones, but is also high in sodium, which may counteract some of its cardiovascular benefits (33). This mirrors the findings from the literature on dairy products, where the sodium content in cheese similarly attenuates the health effects, highlighting again the importance of considering the whole food matrix and not just individual nutrients.

Overall, the favorable health effects of fermented soy are likely due to a number of integrated mechanisms: improved bioavailability of nutrients, generation of new bioactive compounds, enhancement of gut microbiota diversity and synergistic interactions within the food matrix. Taken together, the evidence from observational studies, microbial mechanisms, and cultural dietary practices emphasizes that fermented soy, particularly natto, is a promising dietary component for reducing the risk of CVD mortality.

#### 3.5.4 Fermented vegetables

Fermented vegetables such as sauerkraut, kimchi, and various regional pickled products are valuable sources of organic acids, dietary fiber, and microbial metabolites that can improve mineral absorption, support microbial diversity in the gut and have antioxidant and anti-inflammatory effects. The fermentation process increases the bioactivity of phytochemicals, particularly flavonoids and polyphenols, thereby reducing oxidative stress and modulating chronic diseases (112). Fermented vegetables contain microbial species such as *Lactiplantibacillus plantarum*, *Leuconostoc mesenteroides*, and *Pediococcus* spp., while spontaneously fermented varieties, such as sauerkraut, also host a diverse yeast microbiota including species like *Debaryomyces hansenii*, *Clavispora lusitaniae*, and *Pichia fermentans*, which contribute to the overall microbial complexity and sensory properties of the final product (6, 113).

Despite these promising mechanistic pathways, the epidemiological evidence remains inconclusive. The EPIC-NL cohort study (40) investigated the association between consumption of fermented vegetables and mortality risk and found association with reduction in all-cause mortality and no significant association with CVD mortality. Notably, the median intake reported in the highest consumption tertile was relatively modest (6.4 g/day), suggesting that potential health effects might require higher habitual intake or could be obscured by confounding dietary or lifestyle factors. Another concern is the often high sodium content of fermented vegetables such as kimchi and pickled products, which could counteract the cardioprotective effects (40, 114).

In summary, fermented vegetables provide valuable bioactive compounds and microbial diversity to the diet and may support immune function, mineral bioavailability, and anti-inflammatory responses. However, evidence from prospective cohort studies remains scarce and limited by relatively low intake levels, which may obscure potential associations. Future research should more clearly define dose-response relationships and account for factors such as sodium content.

#### 3.5.5 Fermented meat

Processed meat consumption has been consistently associated with increased mortality risk in epidemiological studies. In the EPIC cohort, higher intake of processed meat (sausages, bacon, ham, and fermented products such as salami and chorizo) was linked to elevated all-cause and CVD mortality (84). The study further estimated that 3.3% of all deaths could be prevented if processed meat consumption was reduced to below 20 g/day. These adverse health effects are mainly attributed to the chemical profile and structural matrix of processed meat. These products typically contain high levels of saturated fat, sodium, and preservatives such as nitrites and nitrates, which promote the formation of N-nitroso compounds (NOCs), potent carcinogens that are particularly implicated in colorectal cancer (115). In addition, processes such as curing, smoking, and cooking at high temperatures produce polycyclic aromatic hydrocarbons (PAHs) and advanced glycation end-products (AGEs), compounds that increase oxidative stress, impair endothelial function, and trigger pro-inflammatory processes that are central to cardiometabolic diseases (115, 116).

Fermented meat (including dry or semi-dry sausages like salami, pepperoni, and regional cured meats), although classified within the broader category of processed meat, is produced by microbial fermentation, typically involving starter cultures of *Lactobacillus* and *Staphylococcus* species (117). Fermentation contributes to storage stability, distinctive flavor profiles, and improved protein digestibility. It also leads to the formation of bioactive peptides, some of which show antioxidant or antihypertensive effects *in vitro* and in animal models. However, the potentially health-promoting compounds produced by fermentation are overshadowed by high sodium concentrations and the persistent presence of nitrites, which remain central to the preservation and coloring of these products (40).

Importantly, most studies do not differentiate fermented meats from other processed meats, limiting our ability to assess whether fermentation itself modifies health outcomes. For this reason, processed meat was not included as a distinct food group in our analysis, as it was not possible to determine the contribution of fermented meat within this broader category. We identified only one prospective study that examined fermented meat separately, which reported an association with CVD mortality in the direction of adverse effects, but neutral relationship with all-cause or cancer mortality. This association was conservatively classified as unclear, reflecting the need for more consistent evidence before drawing firm conclusions.

These findings highlight the need to classify fermented meat separately from other processed meats and to investigate its health effects independently. Developing a more nuanced understanding of fermented meat, particularly its microbial, chemical, and structural characteristics, is essential to determine whether its health impacts align with or differ from those of the broader processed meat category.

#### 3.5.6 Bread

Similar to our observation, several cohort-based meta-analyses have shown a clear difference in health outcomes between the consumption of whole grain and refined bread. Aune et al. conducted a comprehensive dose-response meta-analysis which showed that each 90 g/day increase in whole grain consumption, roughly equivalent to two slices of whole grain bread, was associated with a reduction in the risk of CHD, CVD, total cancer, and all-cause mortality (76). In contrast, refined grain products such as white bread were associated with neutral or even adverse effects (75, 76).

The health implications of bread are best understood in terms of its nutrient matrix. Whole grain bread retains the bran and germ, providing dietary fiber, B vitamins, magnesium, polyphenols, and phytochemicals that regulate oxidative stress, inflammation, and insulin sensitivity (76). These mechanisms are of central importance for the prevention of cardiovascular and neoplastic diseases. In contrast, refined white bread, which has been stripped of fiber and micronutrients, contains mainly rapidly digestible starch, which increases the glycemic load (118). Ultra-processed white bread often exacerbates this effect by containing additives such as emulsifiers and preservatives and offering hardly any dietary fiber (119). Fermentation introduces an important layer of metabolic nuance. Bread made through spontaneous fermentation, such as traditional sourdough, undergoes microbial transformations involving yeasts (*Saccharomyces cerevisiae*, *Kazachstania humilis*) and lactic acid bacteria (*Lactiplantibacillus plantarum*, *Levilactobacillus brevis*, *Fructilactobacillus sanfranciscensis*), which collaborate to break down anti-nutrients like phytates and produce beneficial metabolites (8). These include bioactive peptides, organic acids and SCFAs, which have been associated with improved bioavailability of minerals (e.g., zinc, magnesium, iron), improved glycemic control and reduced systemic inflammation (35, 74). Mechanistically, fermented wholegrain bread can positively influence lipid profile, glucose metabolism, and inflammatory markers such as C-reactive protein (CRP) (120). Although bread consumption alone does not consistently predict lower CVD mortality, its contribution to dietary patterns rich in fermented or fiber-dense foods has been associated with a reduction in risk factors for IHD and metabolic syndrome (35, 42).

To summarize, the health effects of bread are not uniform and depend crucially on the type of grain, fermentation procedure, degree of processing, and general dietary context. Whole grain and fermented breads such as sourdough appear to be metabolically beneficial due to their nutrient density, bioactive potential, and interaction with the gut microbiota. In contrast, a high intake of refined white bread, especially in low-fiber or high-glycemic diets, is associated with an increased risk of chronic disease. These patterns mirror findings in other food categories and emphasize the central role of food matrix and preparation methods in determining health outcomes.

#### 3.5.7 Chocolate and cocoa

The observational cohort studies we encountered and previously published meta-analyses consistently suggest that moderate consumption of chocolate, especially dark chocolate with a high cocoa content, is associated with reduced mortality risk from CVD and all-causes. A dose-response meta-analysis by Morze et al. found that each 10 g/day increase in chocolate consumption was associated with a reduced risk of CHD and stroke (121). The strongest protective associations were observed at intakes of 10–20 g/day, with no further benefit and even possible harm at higher intakes (>30–35 g/day). Zhao et al. (80) found similar patterns in their meta-analysis: high compared to low chocolate consumption was associated with a 12% reduction in all-cause mortality, 13% reduction in CVD mortality, 16% reduction in heart disease mortality, and a 12% reduction in cancer mortality (80).

Although chocolate is not commonly categorized as a fermented food, microbial fermentation is an important step in the processing of cocoa beans. This spontaneous fermentation, which typically involves *Saccharomyces*, *Lactobacillus*, and *Acetobacter* species, breaks down the cocoa pulp and triggers biochemical changes that increase the bioavailability of key flavonoids such as flavan-3-ols and procyanidins (122–124). These polyphenols have shown strong antioxidant, vasodilatory, and anti-inflammatory effects. Mechanistically, they increase nitric oxide bioactivity, improve endothelial function, inhibit platelet aggregation, and reduce LDL oxidation and the production of inflammatory cytokines (56, 77, 79, 80, 124, 125). The type and composition of chocolate have a significant impact on health outcomes. Dark chocolate, which contains a higher percentage of cocoa and more flavanols, has been more consistently associated with protective effects than milk or white chocolate. Milk proteins can bind polyphenols and reduce their absorption, while alkali treatment (Dutch processing) significantly reduces flavonoid content (124). Zhao et al. emphasized that the health benefits observed in meta-analyses are probably due to the consumption of dark chocolate rather than milk chocolate, which has a higher sugar and saturated fat content and a lower polyphenol content (80).

In summary, moderate consumption of cocoa-rich, flavanol-dense dark chocolate may be associated with reduced mortality and improved cardiovascular health. While the fermentation of cocoa improves the availability of bioactive compounds, the matrix of the final product, including the fat, sugar, and milk content, determines its overall health effects.

### 3.6 Strengths and limitations

Strengths of this study include a focus on prospective cohort designs with long follow-up; a large cumulative sample across diverse populations; systematic, transparent procedures for study selection, data extraction, and quality assessment; and a structured, SWiM-aligned framework that supports consistent cross-category interpretation of the indicative direction and strength of associations. We also complement the synthesis with an EFSA-aligned appraisal of biological plausibility and substantiation, integrating mechanistic considerations and regulatory relevance to contextualize the observed relations.

The limitations of this work stem from the evidence base, in which, several fermented-food categories are sparsely represented and exposure measures are not fully standardized, reducing comparability and the precision of category-level estimates. Formal tests for small-study or publication bias were not feasible at the review-wide level or within several categories due to study number and heterogeneity; thus, such bias cannot be ruled out. In addition, many cohorts under-report sex-specific estimates, limiting assessment of potential effect modification. Our objective was to produce a transparent, field-wide synthesis including as many fermented-food categories as possible. However, for some of the categories, for which the evidence is comparable, quantitative pooling would constitute a valuable complement to the present work.

## 4 Conclusion

This systematic review examined the association between the consumption of non-alcoholic fermented foods and mortality risk in healthy adult populations. Overall, the findings suggest that habitual consumption of certain fermented foods is associated with a modest reduction in all-cause and CVD mortality. The evidence in relation to cancer mortality was less consistent and often inconclusive.

Among fermented foods, fermented dairy products are the most extensively studied, with over 30 cohort studies conducted on several continents. The most consistent and robust associations were observed for yogurt and fermented milk, both of which are associated with a lower risk of all-cause and CVD mortality. For cheese, the results were more variable, with potential benefits for all-cause mortality but largely neutral findings for CVD and cancer mortality, possibly influenced by regional variations in fat and sodium content. Fermented soy products, particularly natto, demonstrated promising associations with reduced CVD mortality in Japanese cohorts, although the evidence remains geographically limited. Miso did not show consistent benefits, which may be attributable to its high salt concentration. Other fermented foods, such as fermented vegetables and fermented meat, were less frequently studied. A single European cohort indicated a slight reduction in mortality for fermented vegetables and neutral association with all-cause and cancer mortality for meat. For CVD mortality, the association with fermented meat pointed toward adverse effects, but was conservatively interpreted as unclear due to limited available data. While the hazard ratio indicated a potential increased risk, the authors of the study noted that fermented meat intake was relatively low and potentially underestimated due to limitations in dietary assessment, which may have diluted any true associations (40). The results for bread were also inconclusive, with whole grain bread and sourdough appearing more favorable than refined varieties, which showed more neutral effects. Chocolate and cocoa were associated with modest reductions in CVD and, to a lesser extent, all-cause mortality, although product composition and consumption patterns may influence outcomes.

It is important to note that certain foods, such as fermented meat, have not been studied as separate categories in epidemiological research. Instead, they are typically subsumed under the broader category of processed meat products, which consistently show strong associations with increased risk of mortality and chronic diseases. This categorization obscures potential differential health effects of fermented meat products highlighting a clear research gap that should be addressed in future studies. Similar to meat, foods such as butter and pickles often have an unclear or inconsistently defined fermentation status as well.

Despite these encouraging trends, the current evidence base on non-alcoholic fermented foods and mortality risk does not meet the standards required by the EFSA for substantiating health claims. To strengthen the evidence base, future research should prioritize robust RCTs to confirm causality, determine optimal intake levels, and examine context-specific effects influenced by geography, genetics, and broader dietary patterns. In this regard, studies focusing on intermediate clinical outcomes can provide mechanistic insights and serve as viable surrogates for long-term mortality risk. Inflammatory markers such as high-sensitivity C-reactive protein (hs-CRP), TNF-α and IL-6 are particularly relevant, alongside metabolic indicators such as plasma glucose, insulin, triglycerides, and HDL cholesterol, which have shown associations with fermented food consumption in cohort and intervention studies (126–128). In addition, validated fermentation-dependent metabolites (e.g., mannitol, 2-ethylmalate, methionine, theabrownins, gallic acid, erythritol, citramalate) offer promising objective intake biomarkers, while gut barrier and microbiome indices (e.g., lipopolysaccharide-binding protein, zonulin, conjugated linoleic acid) may help to clarify causal relationships (129–132). The inclusion of such multidomain biomarkers in future studies will strengthen mechanistic understanding and provide evidence consistent with regulatory expectations.

It is also essential to differentiate between traditional and industrially produced fermented foods, as processing methods, including pasteurization, starter culture selection, and standardization techniques, can significantly influence microbial viability, diversity, and the overall composition of the food matrix (133). These variations not only affect sensory and nutritional properties but may also modulate health outcomes by altering the presence and activity of bioactive compounds, thus highlighting the need for more detailed exposure characterization in both epidemiological and clinical research. Underrepresented food categories such as fermented vegetables and fermented meat require further investigation to clarify their mechanistic and epidemiological role. The role of the food matrix, including its structural, compositional and microbial interactions, should be emphasized more than that of nutrients alone. Standardized definitions, validated exposure measurements, and globally representative cohorts will be crucial to ensure consistency and generalizability of future findings.

It is noteworthy that although most studies included both males and females, stratified analyses by sex were rarely presented. Only a limited number of studies examined sex-specific associations, some of which suggested differential effects, particularly for fermented soy products in relation to all-cause mortality and fermented dairy products in relation to CVD mortality. Given the emerging evidence of sex-related variation in dietary response, future research should disaggregate data more consistently by sex to clarify potential differences in the association between consumption of fermented foods and mortality risk.

In summary, while current findings support the potential of fermented foods to modestly reduce risk of mortality, the available evidence remains insufficient for regulatory endorsement. Addressing these methodological and conceptual gaps is key to developing evidence-based dietary guidance on fermented foods.

## Data Availability

The original contributions presented in this study are included in this article/Supplementary material, further inquiries can be directed to the corresponding author.
