# Supplementary material for: A systematic review of prospective evidence linking non-alcoholic fermented food consumption with lower mortality risk
Source: Front Nutr. 2025 Nov 3;12:1657100. doi: 10.3389/fnut.2025.1657100 (PMC12620449; doi:10.3389/fnut.2025.1657100)
Supplement: Supplementary file 1 [file Data_Sheet_1.docx]

Supplementary Material

# Methods

## Literature Search

**Supplementary Table 1.** Search string used in PubMed, Scopus and Cochrane Library.

| **No.** | **Query PubMed** |
| --- | --- |
| #1 | "Fermented Foods"[Mesh] OR "Fermentation"[Mesh] OR ("Food"[Mesh] AND ferment*[tiab]) OR ((ferment*[tiab] OR cultur*[tiab] OR leaven*[tiab]) AND (food*[tiab] OR drink*[tiab] OR beverage*[tiab])) OR "Fermented product"[tiab:~6] OR "Fermented products"[tiab:~6] OR "cultured product"[tiab:~6] OR "cultured products"[tiab:~6] OR "product fermentation"[tiab:~6] OR "products fermentation"[tiab:~6] OR "starter culture*"[tiab] OR ((ferment*[tiab] OR culture*[tiab] OR sour*[tiab]) AND (milk[tiab] OR dairy[tiab])) OR buttermilk[tiab] OR sour cream*[tiab] OR cheese*[tiab] OR yoghurt[tiab] OR yogurt[tiab] OR "yoghourt"[tiab] OR "yakult"[tiab] OR "quark"[tiab] OR "kefir"[tiab] OR "lassi"[tiab] OR "kumis"[tiab] OR "koumiss"[tiab] OR "kajmak"[tiab] OR "airag"[tiab] OR "ayran"[tiab] OR "calpis"[tiab] OR "borhani"[tiab] OR "chal"[tiab] OR "doogh"[tiab] OR kvass[tiab] OR skyr[tiab] OR amasi[tiab] OR bouza[tiab] OR butter*[tiab] OR chal[tiab] OR filmjolk[tiab] OR kishk[tiab] OR labne*[tiab] OR ((Ferment*[tiab] OR cultur*[tiab] OR cured[tiab]) AND (meat*[tiab] OR fish*[tiab] OR seafood*[tiab] OR shellfish[tiab] OR sausage*[tiab])) OR "salami"[tiab] OR "pepperoni"[tiab] OR peperoni[tiab] OR "chorizo"[tiab] OR "cervelat"[tiab] OR "mettwurst"[tiab] OR "summer sausage"[tiab] OR "sucuk"[tiab] OR "dried meat"[tiab] OR "dried sausage"[tiab] OR "dry sausage"[tiab] OR "fish sauce"[tiab] OR "shrimp paste"[tiab] OR "shrimp sauce"[tiab] OR "oyster sauce"[tiab] OR "prosciutto"[tiab] OR "pancetta"[tiab] OR "saucisson"[tiab] OR sucuk[tiab] OR ((Ferment*[tiab] OR cultur*[tiab]) AND (fruit*[tiab] OR vegetable*[tiab] OR coconut*[tiab] OR almond*[tiab] OR hazelnut*[tiab] OR nut[tiab] OR cucumber*[tiab] OR lemon*[tiab] OR citrus[tiab] OR cabbage*[tiab] OR cauliflower*[tiab] OR pepper*[tiab] OR carrot*[tiab] OR olive*[tiab] OR onion*[tiab] OR sago[tiab])) OR "sauerkraut"[tiab] OR "table olive"[tiab] OR pickle*[tiab] OR "kimchi"[tiab] OR "paocai"[tiab] OR torshi[tiab] OR ((Ferment*[tiab] OR cultur*[tiab]) AND (tea[tiab] OR teas[tiab] OR juice*[tiab])) OR "beer"[tiab] OR "wine"[tiab] OR "cider*"[tiab] OR shochu[tiab] OR "kombucha*"[tiab] OR "pulque"[tiab] OR puer[tiab] OR pu’er[tiab] OR pu-er[tiab] OR pu-erh[tiab] OR "pu erh"[tiab] OR "fuzhuan"[tiab] OR "dark tea*"[tiab] OR "yellow tea*"[tiab] OR coffee[tiab] OR shalgam[tiab] OR hardaliye[tiab] OR ((Ferment*[tiab] OR cultur*[tiab]) AND (soy[tiab] OR soya[tiab] OR bean*[tiab] OR pea[tiab] OR peas[tiab] OR lentil*[tiab] OR chickpea*[tiab] OR legume*[tiab] OR pulse*[tiab] OR (poi[tiab])) OR "soy sauce"[tiab] OR "soya sauce"[tiab] OR "soybean paste"[tiab] OR "miso"[tiab] OR "tempeh"[tiab] OR tempe[tiab] OR "natto"[tiab] OR "doenjang"[tiab] OR "doubanjiang"[tiab] OR douchi[tiab] OR "gochujang"[tiab] OR cheonggukjang[tiab] OR tsukemono[tiab] OR garri[tiab] OR ((Ferment*[tiab] OR cultur*[tiab] OR leaven*[tiab]) AND (cereal*[tiab] OR grain*[tiab] OR wheat*[tiab] OR oat[tiab] OR oats[tiab] OR rice*[tiab] OR millet*[tiab] OR sorghum*[tiab] OR maize*[tiab] OR rye[tiab] OR barley*[tiab] OR chia[tiab] OR oilseed*[tiab] OR teff[tiab])) OR "bread"[tiab] OR "sourdough"[tiab] OR "crispbread"[tiab] OR "boza"[tiab] OR "ogi"[tiab] OR dosa[tiab] OR "tarhana"[tiab] OR "buckwheat"[tiab] OR "spelt"[tiab] OR "einkorn"[tiab] OR "quinoa"[tiab] OR "amaranth"[tiab] OR "tef"[tiab] OR "bushera"[tiab] OR chica[tiab] OR chicha[tiab] OR choujiu[tiab] OR injera[tiab] OR mahewu[tiab] OR ogiri[tiab] OR pozol[tiab] OR ugba[tiab]) OR ((Ferment*[tiab] OR cultur*[tiab]) AND ("condiment*"[tiab] OR relish*[tiab] OR horseradish[tiab] OR "dressing*"[tiab] OR "seasoning*"[tiab] OR "sauce*"[tiab] OR cocoa*[tiab] OR tuber[tiab] OR "acetic acid"[tiab])) OR "chocolate*"[tiab] OR "vinegar*"[tiab] OR "tabasco"[tiab] OR "sriracha"[tiab] OR "Worcestershire"[tiab] OR "Worcester"[tiab] |
| #2 | "Diet"[Mesh] OR "Life Style"[Mesh] OR "Eating"[Mesh] OR "Feeding Behavior"[Mesh] OR ((food[tiab] OR macronutrient*[tiab] OR eating[tiab]) AND (intake*[tiab] OR habit*[tiab] OR behavior*[tiab] OR pattern*[tiab])) OR diet*[tiab] OR intake[tiab] OR ingestion[tiab] OR suppl*[tiab] OR consumption[tiab] OR meal*[tiab] OR nutrient*[tiab] OR nutrit*[tiab] |
| #3 | "Mortality"[Mesh] OR "Death"[Mesh] OR "mortality"[Subheading] OR ((Mortalit*[tiab] OR death*[tiab] OR fatal*[tiab] OR survival[tiab]) AND ("Risk Factors"[Mesh] OR factor*[tiab] OR risk*[tiab] OR rate*[tiab] OR hazard ratio*[tiab])) |
| #4 | #1 AND #2 AND #3 |
| #5 | "Diet Surveys"[Mesh] OR "Cohort Studies"[Mesh] OR cohort*[Tiab] OR prospective[Tiab] OR longitudinal[Tiab] |
| #6 | "Epidemiologic Methods"[Mesh:NoExp] OR "Epidemiologic Studies"[Mesh] OR "Observational Studies as Topic"[Mesh] OR "Clinical Studies as Topic"[Mesh] OR "Single-Case Studies as Topic"[Mesh] OR "Organizational Case Studies"[Mesh] OR observational study[Publication Type] OR validation study[Publication Type] OR clinical study[Publication Type] OR case reports[Publication Type] OR "observational study"[tiab:~3] OR "observational studies"[tiab:~3] OR "observational design"[tiab:~3] OR "observational analysis"[tiab:~3] OR "observational analyses"[tiab:~3] OR ((cohort*[tiab] OR prospective[tiab] OR follow-up[tiab] OR longitudinal[tiab] OR long-term[tiab] OR retrospective[tiab]) AND (study[tiab] OR studies[tiab] OR design[tiab] OR analysis[tiab] OR analyses[tiab] OR data[tiab] OR review[tiab])) OR case control*[tiab] OR case comparison*[tiab] OR case-referent[tiab] OR "population study"[tiab:~3] OR "population studies"[tiab:~3] OR "population analysis"[tiab:~3] OR "population analyses"[tiab:~3] OR "descriptive study"[tiab:~3] OR "descriptive studies"[tiab:~3] OR "descriptive design"[tiab:~3] OR "descriptive analysis"[tiab:~3] OR "descriptive analyses"[tiab:~3] OR "multidimensional study"[tiab:~3] OR "multidimensional studies"[tiab:~3] OR "multidimensional design"[tiab:~3] OR "multidimensional analysis"[tiab:~3] OR "multidimensional analyses"[tiab:~3] OR "cross-sectional study"[tiab:~3] OR "cross-sectional studies"[tiab:~3] OR "cross-sectional design"[tiab:~3] OR "cross-sectional analysis"[tiab:~3] OR "cross-sectional analyses"[tiab:~3] OR "cross-sectional research"[tiab:~3] OR "cross-sectional survey"[tiab:~3] OR "cross-sectional findings"[tiab:~3] OR natural experiment*[tiab] OR quasi experiment*[tiab] OR "nonexperimental study"[tiab:~3] OR "nonexperimental studies"[tiab:~3] OR "nonexperimental design"[tiab:~3] OR "nonexperimental analysis"[tiab:~3] OR "nonexperimental analyses"[tiab:~3] OR "prevalence study"[tiab:~3] OR "prevalence studies"[tiab:~3] OR "prevalence analysis"[tiab:~3] OR "prevalence analyses"[tiab:~3] OR case series[tiab] OR "case report"[tiab:~3] OR "case reports"[tiab:~3] OR "case study"[tiab:~3] OR "case studies"[tiab:~3] OR "case histories"[tiab:~3] |
| #7 | "systematic review" |
| #8 | #5 OR #6 OR #7 |
| #9 | #4 AND #8 |
| #10 | #9 NOT (("Child"[Mesh] OR "Infant"[Mesh] OR "Adolescent"[Mesh]) NOT "Adult"[Mesh]) |
| #11 | #10 NOT (("Animals"[Mesh] OR "Animal Experimentation"[Mesh] OR "Models, Animal"[Mesh] OR "Vertebrates"[Mesh]) NOT ("Humans"[Mesh] OR "Human Experimentation"[Mesh])) |
| #12 | #11 NOT ("Breast Feeding"[Majr] OR "Milk, Human"[Majr]) |
| #13 | #12 AND (English[Filter]) |
| #14 | #13 AND (("1970/01/01"[Date - Publication] : "2023/08/31"[Date - Publication])) |
| **No.** | **Query Scopus** |
| #1 | TITLE-ABS-KEY ((ferment* OR cultur* OR leaven*) W/6 (food* OR drink* OR beverage*) OR "starter culture*") OR TITLE-ABS-KEY ((ferment* OR cultur* OR leaven*) W/2 product*) OR TITLE-ABS-KEY (((ferment* OR culture* OR sour*) W/6 (milk OR dairy OR cream* OR quark)) OR buttermilk OR cheese* OR yoghurt OR yogurt OR yoghourt OR yakult OR kefir OR lassi OR kumis OR koumiss OR kajmak OR airag OR ayran OR calpis OR borhani OR chal OR doogh OR kvass OR skyr OR amasi OR bouza OR butter* OR chal OR filmjolk OR kishk OR labne*) OR TITLE-ABS-KEY (((Ferment* OR cultur* OR cured) W/6 (meat* OR fish* OR seafood* OR shellfish OR sausage*)) OR "salami" OR "pepperoni" OR peperoni OR "chorizo" OR "cervelat" OR "mettwurst" OR "summer sausage" OR "sucuk" OR "dried meat*" OR "dried sausage*" OR "dry sausage*" OR "fish sauce*" OR "shrimp paste" OR "shrimp sauce" OR "oyster sauce" OR "prosciutto" OR "pancetta" OR "saucisson" OR sucuk) OR TITLE-ABS-KEY (((Ferment* OR cultur*) W/6 (fruit* OR vegetable* OR coconut* OR almond* OR hazelnut* OR nut OR cucumber* OR lemon* OR citrus OR cabbage* OR cauliflower* OR pepper* OR carrot* OR olive* OR onion* OR sago)) OR "sauerkraut" OR "table olive*" OR pickle* OR "kimchi" OR "paocai" OR torshi) OR TITLE-ABS-KEY (((Ferment* OR cultur*) W/6 (tea OR teas OR juice*)) OR "beer" OR "wine" OR cider* OR shochu OR kombucha* OR "pulque" OR puer OR "pu-er*" OR "fuzhuan" OR "dark tea*" OR "yellow tea*" OR coffee OR shalgam OR hardaliye) OR TITLE-ABS-KEY (((Ferment* OR cultur*) W/6 (soy OR soya OR bean* OR pea OR peas OR lentil* OR chickpea* OR legume* OR pulse* OR poi)) OR "soy* sauce*" OR "soybean paste*" OR miso* OR tempeh* OR tempe OR "natto" OR "doenjang" OR "doubanjiang" OR douchi OR "gochujang" OR cheonggukjang OR tsukemono OR garri) OR TITLE-ABS-KEY (((Ferment* OR cultur* OR leaven*) W/6 (cereal* OR grain* OR wheat* OR oat OR oats OR rice* OR millet* OR sorghum* OR maize* OR rye OR barley* OR chia OR oilseed* OR teff)) OR "bread" OR "sourdough" OR "crispbread" OR "boza" OR "ogi" OR dosa OR "tarhana" OR "buckwheat" OR "spelt" OR "einkorn" OR "quinoa" OR "amaranth" OR "tef" OR "bushera" OR chica OR chicha OR choujiu OR injera OR mahewu OR ogiri OR pozol OR ugba) OR TITLE-ABS-KEY (((Ferment* OR cultur*) W/6 (condiment* OR relish* OR horseradish OR dressing* OR seasoning* OR sauce* OR cocoa* OR tuber OR "acetic acid")) OR chocolate* OR vinegar* OR "tabasco" OR "sriracha" OR "Worcestershire" OR "Worcester") |
| #2 | TITLE-ABS-KEY (((food OR *nutrient* OR eating OR nutrit*) W/6 (intake* OR habit* OR behavior* OR pattern* OR consumption OR suppl* OR ingestion)) OR diet* OR meal*) |
| #3 | TITLE-ABS-KEY ((Mortalit* OR death* OR fatal* OR survival) AND (factor* OR risk* OR rate* OR “hazard ratio*”)) |
| #4 | #1 AND #2 AND #3 *(add combination of string numbers to the field in advanced search in the field "Combined queries…")* |
| #5 | TITLE-ABS-KEY (observational W/3 (study OR studies OR design OR analysis OR analyses)) OR TITLE-ABS-KEY (cohort*) OR TITLE-ABS-KEY (prospective W/7 (study OR studies OR design OR analysis OR analyses)) OR TITLE-ABS-KEY (("follow up" OR followup) W/7 (study OR studies OR design OR analysis OR analyses)) OR TITLE-ABS-KEY ((longitudinal OR longterm OR (long W/1 term)) W/7 (study OR studies OR design OR analysis OR analyses OR data)) OR TITLE-ABS-KEY (retrospective W/7 (study OR studies OR design OR analysis OR analyses OR data OR review)) OR TITLE-ABS-KEY ((case W/1 control) OR (case W/1 comparison) OR (case W/1 controlled)) OR TITLE-ABS-KEY (case-referent W/3 (study OR studies OR design OR analysis OR analyses)) OR TITLE-ABS-KEY (population W/3 (study OR studies OR analysis OR analyses)) OR TITLE-ABS-KEY (descriptive W/3 (study OR studies OR design OR analysis OR analyses)) OR TITLE-ABS-KEY ((multidimensional OR (multi W/1 dimensional)) W/3 (study OR studies OR design OR analysis OR analyses)) OR TITLE-ABS-KEY (cross W/1 sectional W/7 (study OR studies OR design OR research OR analysis OR analyses OR survey OR findings)) OR TITLE-ABS-KEY ((natural W/1 experiment) OR (natural W/1 experiments)) OR TITLE-ABS-KEY (quasi W/1 (experiment OR experiments OR experimental)) OR TITLE-ABS-KEY (("non experiment" OR nonexperiment OR "non experimental" OR nonexperimental) W/3 (study OR studies OR design OR analysis OR analyses)) OR TITLE-ABS-KEY (prevalence W/3 (study OR studies OR analysis OR analyses)) OR TITLE-ABS-KEY ("case series") OR TITLE-ABS-KEY (case W/3 (report OR reports OR study OR studies OR histories)) |
| #6 | TITLE-ABS-KEY ("systematic review") |
| #7 | #5 OR #6 |
| #8 | #4 AND #7 |
| #9 | (KEY (animal* OR nonhuman)) AND NOT (KEY (human*)) |
| #10 | #8 AND NOT #9 |
| #11 | (KEY (infant* OR child*)) AND NOT (KEY (adult* OR aged)) |
| #12 | #10 AND NOT #11 |
|  | *Limit #12 to English using the language filter* |
|  | *Limit #13 to 1970 - 2023 using the Year filter (Range from 1970 to 2023)* |
| **No.** | **Query Cochrane** |
| #1* | (((ferment* OR cultur* OR leaven*) NEAR/6 (food* OR drink* OR beverage* OR product*) OR (starter NEXT culture*)) OR (((ferment* OR culture* OR sour*) NEAR/6 (milk OR dairy OR cream*)) OR buttermilk OR cheese* OR yoghurt OR yogurt OR yoghourt OR yakult OR quark OR kefir OR lassi OR kumis OR koumiss OR kajmak OR airag OR ayran OR calpis OR borhani OR chal OR doogh OR kvass OR skyr OR amasi OR bouza OR butter* OR chal OR filmjolk OR kishk OR labne*) OR (((Ferment* OR cultur* OR cured) NEAR/6 (meat* OR fish* OR seafood* OR shellfish OR sausage*)) OR "salami" OR "pepperoni" OR peperoni OR "chorizo" OR "cervelat" OR "mettwurst" OR "summer sausage" OR "sucuk" OR (dried NEXT meat*) OR (dried NEXT sausage*) OR (dry NEXT sausage*) OR (fish NEXT sauce*) OR "shrimp paste" OR "shrimp sauce" OR "oyster sauce" OR "prosciutto" OR "pancetta" OR "saucisson" OR sucuk) OR (((Ferment* OR cultur*) NEAR/6 (fruit* OR vegetable* OR coconut* OR almond* OR hazelnut* OR nut OR cucumber* OR lemon* OR citrus OR cabbage* OR cauliflower* OR pepper* OR carrot* OR olive* OR onion* OR sago)) OR "sauerkraut" OR (table NEXT olive*) OR pickle* OR "kimchi" OR "paocai" OR torshi) OR (((Ferment* OR cultur*) NEAR/6 (tea OR teas OR juice*)) OR "beer" OR "wine" OR cider* OR shochu OR kombucha* OR "pulque" OR puer OR pu-er* OR "fuzhuan" OR (dark NEXT tea*) OR (yellow NEXT tea*) OR coffee OR shalgam OR hardaliye) OR (((Ferment* OR cultur*) NEAR/6 (soy OR soya OR bean* OR pea OR peas OR lentil* OR chickpea* OR legume* OR pulse* OR poi)) OR (soy* NEXT sauce*) OR (soybean NEXT paste*) OR miso* OR tempeh* OR tempe OR "natto" OR "doenjang" OR "doubanjiang" OR douchi OR "gochujang" OR cheonggukjang OR tsukemono OR garri) OR (((Ferment* OR cultur* OR leaven*) NEAR/6 (cereal* OR grain* OR wheat* OR oat OR oats OR rice* OR millet* OR sorghum* OR maize* OR rye OR barley* OR chia OR oilseed* OR teff)) OR "bread" OR "sourdough" OR "crispbread" OR "boza" OR “ogi” OR dosa OR "tarhana" OR "buckwheat" OR "spelt" OR "einkorn" OR "quinoa" OR "amaranth" OR "tef" OR "bushera" OR chica OR chicha OR choujiu OR injera OR mahewu OR ogiri OR pozol OR ugba) OR (((Ferment* OR cultur*) NEAR/6 (condiment* OR relish* OR horseradish OR dressing* OR seasoning* OR sauce* OR cocoa* OR tuber OR "acetic acid")) OR chocolate* OR vinegar* OR "tabasco" OR "sriracha" OR "Worcestershire" OR "Worcester")):ti,ab,kw |
| #2* | (((food OR macronutrient* OR eating) NEAR/6 (intake* OR habit* OR behavior* OR pattern*)) OR diet* OR intake OR ingestion OR suppl* OR consumption OR meal* OR nutrient* OR nutrit*):ti,ab,kw |
| #3* | ((mortalit* OR death* OR fatal* OR survival) AND (factor* OR risk* OR rate* OR (hazard NEXT ratio*))):ti,ab,kw |
| #4* | #1 AND #2 AND #3 |
| * | Search with filter "Title Abstract Keyword" |
| ** | Choose in the field "Limits" the following options: (a) Content type: "Cochrane Reviews" and "Trials"; (b) Date published on the Cochrane Library: Between "January 1970" and "August 2023" |

## Data analysis and data synthesis – indicative direction of effect and strength of evidence

We conducted a narrative synthesis following the SWiM guideline (Synthesis Without Meta-analysis) and determined how the results would be summarized and classified. To keep findings comparable across heterogeneous cohorts (without implying a precision-weighted pooled estimate), we used a simple unweighted mean hazard ratio (HR) as a descriptive summary for each exposure-outcome pair. Associations were then classified by combining magnitude, consistency, and precision, following GRADE conventions and also narrative GRADE guidelines (1–5). This rule-based approach standardizes terminology, limits subjectivity and preserves interpretability in heterogeneous exposures in narrative reviews.

An association was classified as “reduce” if the arithmetic mean of HRs across included studies was less than 0.90, 25% or fewer of the included studies had confidence intervals (CIs) that overlapped 1.0, and at least 75% of the reported HRs fell within a range narrower than 0.5. The “slightly reduce” was assigned if the mean HR was less than 1.00, the CIs overlapped 1.0, but at least 75% of the HRs fell within a range narrower than 1.0, indicating a generally consistent but more modest effect. This category was intentionally defined to capture subtle or emerging protective trends that may not reach conventional levels of statistical significance, particularly in nutritional epidemiology where effect sizes are often small and confidence intervals may overlap 1.0 due to heterogeneity or limited power. The designation “neutral” was used if the mean HR was less than 1.10, the CIs overlapped 1.0, and at least 75% of the HRs fell within a range narrower than 2.0, indicating little to no meaningful or interpretable effect. Exposure-outcome pairs were categorized as “unclear” if the results were highly inconsistent, such as when the HR range exceeded 2.0, the mean HR was near 1.0 but the CIs overlapped considerably, or if no clear directional trend was apparent. Conversely, results would be classified as “increase” if the majority of CIs lay entirely above 1.0, indicating a consistent trend toward elevated risk. However, no such pattern emerged in the current analysis. The duration of follow-up was summarized as a range, and the geographical regions of the studies involved were documented to facilitate contextual interpretation.

The strength of evidence was assessed using a structured framework that included the number of studies, cumulative sample size, average quality of studies based on the Newcastle-Ottawa scale (NOS), and the level of precision of effect estimates. Imprecision was assessed based on the distribution of 95% CI widths across studies. Imprecision was categorized as not serious if at least 75% of the studies had CI widths below 0.5, moderate if at least 75% of the CI widths were below 1.0, and very serious if 75% or more of the CI widths were above 1.0. Based on these criteria, evidence was categorized as “high” if there were at least five studies, a mean NOS score of 8.0 or more, a cumulative sample size of more than 200,000 participants and no serious imprecision. “Moderate” evidence was defined as having at least three studies of moderate to high quality (NOS ≥7.0) and no serious or only moderate imprecision. A “low” rating was assigned for exposure-outcome pairs supported by at least two studies with NOS scores of 6.0 or higher. A “very low” rating was used if only one study was available, the average NOS score was below 6.0, or the imprecision was considered very serious.

# Supplementary Table 2. Association of total fermented food, fermented vegetables, and fermented meat with all-cause mortality and cause-specific mortality.

| **Study & subgroup details** | **Region** | **Start year** | **Cohort** | **Age range at entry** | **Sex** | **Dietary assessm.** | **Exposure levels** | **No. of subjects** | **No. of deaths** | **Years of FU** | **HR (95% CI)** | **p-value** | **NOS** | **Adjustments** | **Ref.** |
| --- | --- | --- | --- | --- | --- | --- | --- | --- | --- | --- | --- | --- | --- | --- | --- |
| **Fermented foods** | | | | | | | | | | | | | | | |
| **Fermented foods / All-causes** | | | | | | | | | | | | | | | |
| Praagman et al. 2015 | Netherlands | 1992 | EPIC-NL | 20–70 | Male & female | FFQ | Q4: 398.6 g/d (energy-adj.)  Q1: 46.1 g/day (ref.)* | 34409 | 2436 | 15 | 1.0 (0.88-1.13) | 0.6 | 8 | Age, sex, total energy intake, smoking habit, BMI, physical activity, education level, hypertension at baseline, intake of alcohol, energy-adjusted intakes of fruit and vegetables | (6) |
| **Fermented foods / CVD** | | | | | | | | | | | | | | | |
| Praagman et al. 2015 | Netherlands | 1992 | EPIC-NL | 20–70 | Male & female | FFQ | Q4: 398.6 g/d (energy-adj.)  Q1: 46.1 g/day (ref.)* | 34409 | 727 | 15 | 1.04 (0.83-1.3) | 0.7 | 8 | Age, sex, total energy intake, smoking habit, BMI, physical activity, education level, hypertension at baseline, intake of alcohol, energy-adjusted intakes of fruit and vegetables | (6) |
| **Fermented foods / Cancer** | | | | | | | | | | | | | | | |
| Praagman et al. 2015 | Netherlands | 1992 | EPIC-NL | 20–70 | Male & female | FFQ | Q4: 398.6 g/d (energy-adj.)  Q1: 46.1 g/day (ref.)* | 34409 | 1216 | 15 | 1.02 (0.86-1.21) | 0.9 | 8 | Age, sex, total energy intake, smoking habit, BMI, physical activity, education level, hypertension at baseline, intake of alcohol, energy-adjusted intakes of fruit and vegetables | (6) |
| **Fermented vegetables** | | | | | | | | | | | | | | | |
| **Fermented vegetables / All-causes** | | | | | | | | | | | | | | | |
| Praagman et al. 2015 | Netherlands | 1992 | EPIC-NL | 20–70 | Male & female | FFQ | Q4: 6.4 g/d (energy-adj.)  Q1: 1.1 g/day (ref.)* | 34409 | 2436 | 15 | 0.88 (0.78-1.0) | 0.034 | 8 | Age, sex, total energy intake, smoking habit, BMI, physical activity, education level, hypertension at baseline, intake of alcohol, energy-adjusted intakes of fruit and vegetables (adjusted for total vegetable intake without fermented vegetable intake) | (6) |
| **Fermented vegetables / CVD** | | | | | | | | | | | | | | | |
| Praagman et al. 2015 | Netherlands | 1992 | EPIC-NL | 20–70 | Male & female | FFQ | Q4: 6.4 g/d (energy-adj.)  Q1: 1.1 g/day (ref.)* | 34409 | 727 | 15 | 1.05 (0.83-1.32) | 0.7 | 8 | Age, sex, total energy intake, smoking habit, BMI, physical activity, education level, hypertension at baseline, intake of alcohol, energy-adjusted intakes of fruit and vegetables (adjusted for total vegetable intake without fermented vegetable intake) | (6) |
| **Fermented vegetables / Cancer** | | | | | | | | | | | | | | | |
| Praagman et al. 2015 | Netherlands | 1992 | EPIC-NL | 20–70 | Male & female | FFQ | Q4: 6.4 g/d (energy-adj.)  Q1: 1.1 g/day (ref.)* | 34409 | 1216 | 15 | 0.93 (0.77-1.11) | 0.3 | 8 | Age, sex, total energy intake, smoking habit, BMI, physical activity, education level, hypertension at baseline, intake of alcohol, energy-adjusted intakes of fruit and vegetables (adjusted for total vegetable intake without fermented vegetable intake) | (6) |
| **Fermented meat** | | | | | | | | | | | | | | | |
| **Fermented meat / All-causes** | | | | | | | | | | | | | | | |
| Praagman et al. 2015 | Netherlands | 1992 | EPIC-NL | 20–70 | Male & female | FFQ | Q4: 15.9 g/d (energy-adj.)  Q1: 0.6 g/day (ref.)* | 34409 | 2436 | 15 | 1.0 (0.89-1.12) | 0.9 | 8 | Age, sex, total energy intake, smoking habit, BMI, physical activity, education level, hypertension at baseline, intake of alcohol, energy-adjusted intakes of fruit and vegetables | (6) |
| **Fermented meat / CVD** | | | | | | | | | | | | | | | |
| Praagman et al. 2015 | Netherlands | 1992 | EPIC-NL | 20–70 | Male & female | FFQ | Q4: 15.9 g/d (energy-adj.)  Q1: 0.6 g/day (ref.)* | 34409 | 727 | 15 | 1.17 (0.95-1.44) | 0.034 | 8 | Age, sex, total energy intake, smoking habit, BMI, physical activity, education level, hypertension at baseline, intake of alcohol, energy-adjusted intakes of fruit and vegetables | (6) |
| **Fermented meat / Cancer** | | | | | | | | | | | | | | | |
| Praagman et al. 2015 | Netherlands | 1992 | EPIC-NL | 20–70 | Male & female | FFQ | Q4: 15.9 g/d (energy-adj.)  Q1: 0.6 g/day (ref.)* | 34409 | 1216 | 15 | 0.99 (0.84-1.17) | 0.8 | 8 | Age, sex, total energy intake, smoking habit, BMI, physical activity, education level, hypertension at baseline, intake of alcohol, energy-adjusted intakes of fruit and vegetables | (6) |

*Median **Mean

CI, confidence interval; CVD, cardiovascular disease; FFQ, food frequency questionnaire; FU, follow-up; HR, hazard ratio; NOS, Newcastle-Ottawa Scale; Ref., reference.

# Supplementary Table 3. Association of fermented dairy products with all-cause mortality and cause-specific mortality.

| **Study & subgroup details** | **Region** | **Start year** | **Cohort** | **Age range at entry** | **Sex** | **Dietary assessm.** | **Exposure levels** | **No. of subjects** | **No. of deaths** | **Years of FU** | **HR (95% CI)** | **p-value** | **NOS** | **Adjustments** | **Ref.** |
| --- | --- | --- | --- | --- | --- | --- | --- | --- | --- | --- | --- | --- | --- | --- | --- |
| **Fermented dairy** | | | | | | | | | | | | | | | |
| **Fermented dairy / All-causes** | | | | | | | | | | | | | | | |
| Soedamah-Muthu et al. 2013 | UK | 1997 | Whitehall II cohort | 35–55 | Male & female | FFQ | T3: 105 g/day (energy-adj.)  T1: 17 g/day (ref.)* | 4526 | 237 | 11.7 | 0.65 (0.47-0.9) | 0.01 | 7 | Age, ethnicity, employment grade, smoking, BMI, alcohol intake, physical activity, family history of CHD/hypertension, fruit and vegetable, bread, meat, fish, coffee and tea intake | (7) |
| van Aerde et al. 2013 | Netherlands | 1989 | The Hoorn Study | 50–75 | Male & female | FFQ | Q4: ≥14.7 g/day Q1: ≤6.0 g/day (ref.)* | 1956 | 403 | 12.4 | 0.98 (0.87-1.11) | 0.77 | 8 | Age, sex, BMI, smoking, educational level, total energy intake, alcohol consumption, physical activity, intake of meat, fish, bread, vegetables, fruit, coffee, tea | (8) |
| Virtanen et al. 2019 | Finland | 1984 | KIHD | 42–60 | Male | 4-day food record | Q4: 437 g/day (energy-adj.)  Q1: 3 g/day (ref.)* | 2641 | 1225 | 22.3 | 1.04 (0.89-1.21) | 0.53 | 8 | Age, examination year, energy intake, income, education years, marital status, leisure-time physical activity, pack-years of smoking, alcohol intake, BMI, diagnosis of type 2 diabetes, cardiovascular disease, cancer, or hypertension or use of cardiac, hypercholesterolemia, hypertension, or diabetes medications, intakes of fiber and saturated, monounsaturated, polyunsaturated, trans fatty acids | (9) |
| Guo et al. 2022 | Denmark | 1982 | MONICA | 30–60 | Male & female | 7-day weighed food record | Q4: 249.7 g/week  Q1: 12.6 g/week** | 1746 | 660 | 30 | 0.99 (0.78-1.26) | 0.7 | 8 | Sex, BMI, food energy intake, alcohol consumption, education, smoking, physical activity, family history of myocardial infarction, multivitamin use, serum total cholesterol, serum triaclyglycerols, incidence of hypertension | (10) |
| **Fermented dairy / CVD** | | | | | | | | | | | | | | | |
| van Aerde et al. 2013 | Netherlands | 1989 | The Hoorn Study | 50–75 | Male & female | FFQ | Q4: ≥14.7 g/day Q1: ≤6.0 g/day (ref.)* | 1956 | 116 | 12.4 | 1.01 (0.8-1.27) | 0.97 | 8 | Age, sex, BMI, smoking, educational level, total energy intake, alcohol consumption, physical activity, intake of meat, fish, bread, vegetables, fruit, coffee, tea | (8) |
| Praagman et al. 2015 (Stroke M.) | Netherlands | 1990 | Rotterdam Study | ≥ 55 | Male & female | SFFQ | T3: >100 g/day  T1: <50 g/day (ref.)* | 4235 | 182 | 17.3 | 0.85 (0.59-1.22) | 0.85 | 8 | Age, sex, total energy intake, BMI, smoking, education level, alcohol intake, intakes of vegetables, fruit, meat, bread, fish coffee, tea | (11) |
| Praagman et al. 2015 (CHD M.) | Netherlands | 1990 | Rotterdam Study | ≥ 55 | Male & female | SFFQ | T3: >100 g/day  T1: <50 g/day (ref.)* | 4235 | 350 | 17.3 | 0.92 (0.71-1.19) | 0.55 | 8 | Age, sex, total energy intake, BMI, smoking, education level, alcohol intake, intakes of vegetables, fruit, meat, bread, fish coffee, tea | (11) |
| Silva et al. 2022 | Brazil | 2008 | ELSA-Brasil | 35–74 | Male & female | FFQ | Males  Q4: ≥ 361.5 g/day (energy-adj.)  Q1: ≤ 102.8 g/day (ref.)  Females  Q4: ≥ 479.1 g/day (energy-adj.)  Q1: ≤ 187.3 g/day (ref.)* | 6671 | 42 | 8 | 1.34 (0.53-3.36) |  | 8 | Age, sex, educational level, physical activity, smoking status, alcohol consumption, BMI, diabetes, hypertension, hypercholesterolemia | (12) |

| **Study & subgroup details** | **Region** | **Start year** | **Cohort** | **Age range at entry** | **Sex** | **Dietary assessm.** | **Exposure levels** | **No. of subjects** | **No. of deaths** | **Years of FU** | **HR (95% CI)** | **p-value** | **NOS** | **Adjustments** | **Ref.** |
| --- | --- | --- | --- | --- | --- | --- | --- | --- | --- | --- | --- | --- | --- | --- | --- |
| **Fermented milks** | | | | | | | | | | | | | | | |
| **Fermented milks / All-causes** | | | | | | | | | | | | | | | |
| Bonthuis et al. 2010 | Australia | 1992 | Nambour Skin Cancer Study | 25–78 | Male & female | FFQ | Q3: 76 g/day Q1: 0 g/day (ref.)* | 1529 | 177 | 14.4 | 1.22 (0.77-1.93) | 0.36 | 8 | Age, sex, total energy intake, BMI, alcohol intake, school leaving age, physical activity level, pack-years of smoking, dietary supplement use, beta-carotene treatment during trial, presence of any medical condition, dietary calcium | (13) |
| Goldbohm et al. 2011 (Female LF Milk) | Netherlands | 1986 | NLCS | 55–69 | Male & female (data shown for females) | FFQ | Q3/C3: 192 g/day  Q1/C1: 0 g/day (ref.)* | 62573 | 5478 | 10 | 1.02 (0.95-1.09) | 0.265 | 8 | Age, education, smoking, physical activity, BMI, multivitamin use, alcohol, energy, energy-adjusted mono- and polyunsaturated fat intakes, vegetable and fruit consumption | (14) |
| Goldbohm et al. 2011 (Female FF Milk) | Netherlands | 1986 | NLCS | 55–69 | Male & female (data shown for females) | FFQ | Q2/C2: 53 g/day  Q1/C1: 0 g/day (ref.)* | 62573 | 5478 | 10 | 0.93 (0.87-1.0) |  | 8 | Age, education, smoking, physical activity, BMI, multivitamin use, alcohol, energy, energy-adjusted mono- and polyunsaturated fat intakes, vegetable and fruit consumption | (14) |
| Goldbohm et al. 2011 (Male LF Milk) | Netherlands | 1986 | NLCS | 55–69 | Male & female (data shown for males) | FFQ | Q3/C3: 146 g/day  Q1/C1: 0 g/day (ref.)* | 58279 | 10658 | 10 | 0.97 (0.93-1.03) | 0.893 | 8 | Age, education, smoking, physical activity, BMI, multivitamin use, alcohol, energy, energy-adjusted mono- and polyunsaturated fat intakes, vegetable and fruit consumption | (14) |
| Goldbohm et al. 2011 (Male FF Milk) | Netherlands | 1986 | NLCS | 55–69 | Male & female (data shown for males) | FFQ | Q2/C2: 53 g/day  Q1/C1: 0 g/day (ref.)* | 58279 | 10658 | 10 | 0.93 (0.88-0.98) |  | 8 | Age, education, smoking, physical activity, BMI, multivitamin use, alcohol, energy, energy-adjusted mono- and polyunsaturated fat intakes, vegetable and fruit consumption | (14) |
| Sluik et al. 2014 | Multiple | 1992 | EPIC | 45–64 | Male & female | FFQ | Q3: 71 g/day (energy-adj.)  Q1: 3 g/day (ref.)* | 258911 | 12135 | 9.9 | 1 (1.0-1.0) | 0.21 | 8 | Age, region, sex, educational attainment, alcohol consumption, physical activity, smoking status and smoking intensity, factor loadings for the first three dietary patterns derived from factor analysis on 26 food groups | (15) |
| Praagman et al. 2015 | Netherlands | 1993 | EPIC-NL | 20–70 | Male & female | FFQ | Q4: 144.5 g/day (energy-adj.)  Q1: 3.8 g/day (ref.)* | 34409 | 2436 | 15 | 0.97 (0.86-1.09) | 0.9 | 8 | Age, sex, smoking habit, BMI, physical activity, education level, hypertension at baseline, intakes of alcohol and energy-adjusted intakes of fruit and vegetables, total energy intake | (6) |
| Bongard et al. 2016 | France | 1995 | MONICA | 45–64 | Male | 3-day food record | Q4: 1188 g/day (energy-adj.)  Q1: 0 g/day (ref.)* | 960 | 150 | 14.8 | 0.96 (0.62-1.49) | 0.35 | 8 | Center, age, payment of income tax, obesity, alcohol consumption, smoking habits, physical activity, presence of a serious chronic condition, diet quality score | (16) |
| Tognon et al. 2017 | Sweden | 1986 | NSHDS | 24–74 | Male & female | FFQ | Q4: ≥2.5 times/day (energy-adj.)  Q1: <1 times/week (ref.) | 103256 | 6892 | 13.7 | 0.96 (0.92-1.01) |  | 8 | Age, sex, BMI, screening year, smoking, education, energy intake | (17) |
| Farvid et al. 2017 | Iran | 2004 | Golestan Study | 36–85 | Male & female | FFQ | Q5: 0.9 servings/day  Q1: 0.1 servings/day (ref.)** | 42403 | 3291 | 8 | 0.89 (0.89-1.0) | 0.03 | 8 | Age, ethnicity, education, marital status, residency, smoking, opium use, alcohol use, BMI, systolic blood pressure, occupational physical activity, family history of cancer, wealth score, medication use, energy intake | (18) |
| Dehghan et al. 2018 | Multiple | 2003 | PURE | 35–70 | Male & female | FFQ | Q4: 1.5 servings/day Q1: 0 servings/day (ref.)** | 136384 | 6796 | 9.1 | 0.83 (0.69-0.99) | 0.0051 | 8 | Age, sex, education, urban or rural location, smoking, physical activity, history of diabetes, family history of cardiovascular disease, family history of cancer, quintiles of fruit, vegetable, red meat, starchy foods intake, total energy intake, centre was included as a random effect to account for clustering by location | (19) |
| Pala et al. 2019 | Italy | 1993 | EPIC-Italy | 45–64 | Male & female | FFQ | Q4: >120 g/day  Q1 : 0 g/day (ref.) | 45009 | 2468 | 14.9 | 0.95 (0.82-1.09) | 0.14 | 8 | Region, sex, age, energy intake, weight, height, waist-to-hip ratio, alcohol consumption, smoking status, physical activity, relative index of inequality, Italian Mediterranean Index, intake of sugar | (20) |
| Mazidi et al. 2019 | USA | 1999 | NHANES | > 20 | Male & female | 24-h recall | Q4: 3.08 cup eq servings/d Q1: 0.25 cup eq/day (ref.)* | 24474 | 3520 | 6.4 | 0.93 (0.85-1.01) | 0.523 | 9 | Age, sex, race, education, marital status, poverty to income ratio, total energy intake, physical activity, smoking, alcohol consumption, carbohydrates, saturated fat, protein, dietary fiber, BMI, hypertension, diabetes | (21) |
| Schmid et al. 2020 (Female) | USA | 1980 | NHS | 30–59 | Male & female (data shown for females) | SFFQ | >4 servings/week  Never (ref.) | 82348 | 20831 | 32 | 0.91 (0.85-0.98) | 0.34 | 8 | Height, BMI, BMI at age 18 (females) or 21 (males), race, physical activity, smoking status, history of hypertension, history of hypercholesterolemia, history of diabetes, family history of cancer, family history of diabetes, family history of myocardial infarction, current multivitamin use, regular aspirin use, menopausal status and hormone use (only for females), total caloric intake, alcohol consumption, glycemic load, intakes of unprocessed red meat, processed meat, nuts, fruits, vegetables, total calcium, total fiber | (22) |
| Schmid et al. 2020 (Male) | USA | 1980 | NHS | 40–79 | Male & female (data shown for males) | SFFQ | >4 servings/week  Never (ref.) | 40278 | 12397 | 26 | 1.05 (0.95-1.16) | 0.7 | 8 | Height, BMI, BMI at age 18 (females) or 21 (males), race, physical activity, smoking status, history of hypertension, history of hypercholesterolemia, history of diabetes, family history of cancer, family history of diabetes, family history of myocardial infarction, current multivitamin use, regular aspirin use, menopausal status and hormone use (only for females), total caloric intake, alcohol consumption, glycemic load, intakes of unprocessed red meat, processed meat, nuts, fruits, vegetables, total calcium, total fiber | (22) |
| Nakanishi et al. 2021 | Japan | 2009 | Yamagata Study | 40–74 | Male & female | FFQ | High (>1 times/day) None (<1 times/month) (ref.) | 14264 | 265 | 9 | 0.7 (0.49-0.99) | 0.04 | 7 | Age, sex, smoking status, alcohol consumption, BMI, hypertension, diabetes, education | (23) |
| Sonestedt et al. 2021 | Sweden | 1991 | MDCS | 45–73 | Male & female | SFFQ, food record, and interview | C5: > 300 g/day C1: 0 g/day (ref.) | 26190 | 7156 | 19 | 0.9 (0.79-1.03) | 0.009 | 9 | Age, sex, diet assessment method, season, energy, BMI, education, physical activity, smoking, alcohol habits, diet (fruit and vegetables, meat, fiber, sugar-sweetened beverages) | (24) |
| Lin et al. 2022 | USA | 1999 | NHANES | > 18 | Male & female | 24-h recall | NR | 32625 | 3881 | 8.1 | 0.83 (0.71-0.98) | 0.035 | 9 | Age, sex, race, BMI, white blood cell count, hemoglobin, platelet count, total bilirubin, creatinine, blood urea nitrogen, hypertension, diabetes, asthma congestive heart failure, coronary heart disease, stroke, chronic bronchitis, and cancer | (25) |
| Lu et al. 2022 (Male) | Japan | 1990 | Miyagi Cohort | 40–64 | Male & female (data shown for males) | FFQ | 3 times/week or almost daily  Almost never (ref.) | 16565 | 4354 | 25 | 1.04 (0.92-1.17) | 0.253 | 9 | Age (continuous), education level, BMI, smoking status, alcohol drinking status, history of hypertension, history of diabetes, energy intake, fish intake, vegetable and fruit intake | (26) |
| Lu et al. 2022 (Female) | Japan | 1990 | Miyagi Cohort | 40–64 | Male & female (data shown for females) | FFQ | 3 times/week or almost daily  Almost never (ref.) | 17596 | 2522 | 25 | 0.92 (0.81-1.03) | 0.146 | 9 | Age (continuous), education level, BMI, smoking status, alcohol drinking status, history of hypertension, history of diabetes, energy intake, fish intake, vegetable and fruit intake | (26) |
| Guo et al. 2022 | Denmark | 1982 | MONICA | 30–60 | Male & female | 7-day weighed food record | Q4: 194.7 g/week  Q1: 0 g/week** | 1746 | 660 | 30 | 1.05 (0.76-1.45) | 0.95 | 8 | Sex, BMI, food energy intake, alcohol consumption, education, smoking, physical activity, family history of myocardial infarction, multivitamin use, serum total cholesterol, serum triaclyglycerols, incidence of hypertension | (10) |
| Ge et al. 2023 (Male) | Japan | 1995 | JPHC | 40–69 | Male & female (data shown for males) | FFQ | Q4: 76.6 g/day (energy-adj.)  Q1: 0 g/day (ref.)* | 43117 | 14211 | 19.3 | 0.94 (0.9-0.995) | 0.02 | 7 | Age, study area, smoking status, alcohol frequency, BMI, physical activity, hypertension with medication, self, reported diabetes, green tea, coffee, energy-adjusted consumption of vegetables and fruits, total energy and total fat, menopausal status (only for females), exogenous hormone use (only for females), dairy intake | (27) |
| Ge et al. 2023 (Female) | Japan | 1995 | JPHC | 40–69 | Male & female (data shown for females) | FFQ | Q4: 100.8 g/day (energy-adj.)  Q1: 0 g/day (ref.)* | 50193 | 9547 | 19.3 | 0.93 (0.88-0.99) | 0.15 | 7 | Age, study area, smoking status, alcohol frequency, BMI, physical activity, hypertension with medication, self, reported diabetes, green tea, coffee, energy-adjusted consumption of vegetables and fruits, total energy and total fat, menopausal status (only for females), exogenous hormone use (only for females), dairy intake | (27) |
| Miyagawa et al. 2024 (Female) | Japan | 2005 | J-MICC | 35–69 | Male & female (data shown for females) | FFQ | T3: 61.6 g/day (energy-adj.)  T1: 5.5 g/day (ref.)* | 45597 | 1344 | 12 | 0.87 (0.76-0.997) | 0.046 | 9 | Age, study site, history of cardiometabolic diseases, BMI, smoking status, drinking status, physical activity, dietary intake of red meat, fish, vegetables, fruits | (28) |
| Miyagawa et al. 2024 (Male) | Japan | 2005 | J-MICC | 35–69 | Male & female (data shown for males) | FFQ | T3: 40.2 g/day (energy-adj.)  T1: 0 g/day (ref.)* | 34118 | 2379 | 12 | 0.9 (0.82-0.999) | 0.034 | 9 | Age, study site, history of cardiometabolic diseases, BMI, smoking status, drinking status, physical activity, dietary intake of red meat, fish, vegetables, fruits | (28) |
| **Fermented milks / CVD** | | | | | | | | | | | | | | | |
| Bonthuis et al. 2010 | Australia | 1992 | Nambour Skin Cancer Study | 25–78 | Male & female | FFQ | Q3: 76 g/day Q1: 0 g/day (ref.)* | 1529 | 61 | 14.4 | 0.65 (0.26-1.58) | 0.52 | 8 | Age, sex, total energy intake, BMI, alcohol intake, school leaving age, physical activity level, pack-years of smoking, dietary supplement use, beta-carotene treatment during trial, presence of any medical condition, dietary calcium | (13) |
| Goldbohm et al. 2011 (Male IHD M., FF Milk) | Netherlands | 1986 | NLCS | 55–69 | Male & female (data shown for males) | FFQ | Q2/C2: 53 g/day  Q1/C1: 0 g/day (ref.)* | 58279 | 1997 | 10 | 0.77 (0.64-0.92) |  | 8 | Age, education, smoking, physical activity, BMI, multivitamin use, alcohol, energy, energy-adjusted mono- and polyunsaturated fat intakes, vegetable and fruit consumption | (14) |
| Goldbohm et al. 2011 (Female Stroke M., LF Milk) | Netherlands | 1986 | NLCS | 55–69 | Male & female (data shown for females) | FFQ | Q3/C3: 192 g/day  Q1/C1: 0 g/day (ref.)* | 62573 | 322 | 10 | 0.76 (0.55-1.05) | 0.032 | 8 | Age, education, smoking, physical activity, BMI, multivitamin use, alcohol, energy, energy-adjusted mono- and polyunsaturated fat intakes, vegetable and fruit consumption | (14) |
| Goldbohm et al. 2011 (Female Stroke M., FF Milk) | Netherlands | 1986 | NLCS | 55–69 | Male & female (data shown for females) | FFQ | Q2/C2: 53 g/day  Q1/C1: 0 g/day (ref.)* | 62573 | 322 | 10 | 0.81 (0.6-1.1) |  | 8 | Age, education, smoking, physical activity, BMI, multivitamin use, alcohol, energy, energy-adjusted mono- and polyunsaturated fat intakes, vegetable and fruit consumption | (14) |
| Goldbohm et al. 2011 (Male Stroke M., LF Milk) | Netherlands | 1986 | NLCS | 55–69 | Male & female (data shown for males) | FFQ | Q3/C3: 146 g/day  Q1/C1: 0 g/day (ref.)* | 58279 | 520 | 10 | 0.84 (0.64-1.11) | 0.382 | 8 | Age, education, smoking, physical activity, BMI, multivitamin use, alcohol, energy, energy-adjusted mono- and polyunsaturated fat intakes, vegetable and fruit consumption | (14) |
| Goldbohm et al. 2011 (Male Stroke M., FF Milk) | Netherlands | 1986 | NLCS | 55–69 | Male & female (data shown for males) | FFQ | Q2/C2: 53 g/day  Q1/C1: 0 g/day (ref.)* | 58279 | 520 | 10 | 0.86 (0.65-1.15) |  | 8 | Age, education, smoking, physical activity, BMI, multivitamin use, alcohol, energy, energy-adjusted mono- and polyunsaturated fat intakes, vegetable and fruit consumption | (14) |
| Goldbohm et al. 2011 (Female IHD M., LF Milk) | Netherlands | 1986 | NLCS | 55–69 | Male & female (data shown for females) | FFQ | Q3/C3: 192 g/day  Q1/C1: 0 g/day (ref.)* | 62573 | 692 | 10 | 1.19 (0.94-1.51) | 0.056 | 8 | Age, education, smoking, physical activity, BMI, multivitamin use, alcohol, energy, energy-adjusted mono- and polyunsaturated fat intakes, vegetable and fruit consumption | (14) |
| Goldbohm et al. 2011 (Female IHD M., FF Milk) | Netherlands | 1986 | NLCS | 55–69 | Male & female (data shown for females) | FFQ | Q2/C2: 53 g/day  Q1/C1: 0 g/day (ref.)* | 62573 | 692 | 10 | 0.99 (0.79-1.24) |  | 8 | Age, education, smoking, physical activity, BMI, multivitamin use, alcohol, energy, energy-adjusted mono- and polyunsaturated fat intakes, vegetable and fruit consumption | (14) |
| Goldbohm et al. 2011 (Male IHD M., LF Milk) | Netherlands | 1986 | NLCS | 55–69 | Male & female (data shown for males) | FFQ | Q3/C3: 146 g/day  Q1/C1: 0 g/day (ref.)* | 58279 | 1997 | 10 | 0.93 (0.78-1.11) | 0.961 | 8 | Age, education, smoking, physical activity, BMI, multivitamin use, alcohol, energy, energy-adjusted mono- and polyunsaturated fat intakes, vegetable and fruit consumption | (14) |
| Praagman et al. 2015 | Netherlands | 1993 | EPIC-NL | 20–70 | Male & female | FFQ | Q4: 144.5 g/day (energy-adj.)  Q1: 3.8 g/day (ref.)* | 34409 | 727 | 15 | 0.98 (0.79-1.22) | 0.9 | 8 | Age, sex, smoking habit, BMI, physical activity, education level, hypertension at baseline, intakes of alcohol and energy-adjusted intakes of fruit and vegetables, total energy intake | (6) |
| Praagman et al. 2015 (Stroke M.) | Netherlands | 1990 | Rotterdam Study | ≥ 55 | Male & female | SFFQ | T3: >100 g/day  T1: <50 g/day (ref.)* | 4235 | 182 | 17.3 | 1.01 (0.71-1.44) | 0.93 | 8 | Age, sex, total energy intake, BMI, smoking, education level, alcohol intake, intakes of vegetables, fruit, meat, bread, fish coffee, tea | (11) |
| Praagman et al. 2015 (CHD M.) | Netherlands | 1990 | Rotterdam Study | ≥ 55 | Male & female | SFFQ | T3: >100 g/day  T1: <50 g/day (ref.)* | 4235 | 350 | 17.3 | 0.98 (0.76-1.26) | 0.84 | 8 | Age, sex, total energy intake, BMI, smoking, education level, alcohol intake, intakes of vegetables, fruit, meat, bread, fish coffee, tea | (11) |
| Farvid et al. 2017 | Iran | 2004 | Golestan Study | 36–85 | Male & female | FFQ | Q5: 0.9 servings/day  Q1: 0.1 servings/day (ref.)** | 42403 | 1467 | 8 | 0.84 (0.7-1.0) | 0.03 | 8 | Age, ethnicity, education, marital status, residency, smoking, opium use, alcohol use, BMI, systolic blood pressure, occupational physical activity, family history of cancer, wealth score, medication use, energy intake | (18) |
| Pala et al. 2019 | Italy | 1993 | EPIC-Italy | 45–64 | Male & female | FFQ | Q4: >120 g/day  Q1 : 0 g/day (ref.) | 45009 | 249 | 14.9 | 0.85 (0.59-1.23) | 0.15 | 8 | Region, sex, age, energy intake, weight, height, waist-to-hip ratio, alcohol consumption, smoking status, physical activity, relative index of inequality, Italian Mediterranean Index, intake of sugar | (20) |
| Mazidi et al. 2019 | USA | 1999 | NHANES | > 20 | Male & female | 24-h recall | Q4: 3.08 cup eq servings/d Q1: 0.25 cup eq/day (ref.)* | 24474 | 709 | 6.4 | 0.98 (0.97-0.99) | 0.125 | 9 | Age, sex, race, education, marital status, poverty to income ratio, total energy intake, physical activity, smoking, alcohol consumption, carbohydrates, saturated fat, protein, dietary fiber, BMI, hypertension, diabetes | (21) |
| Schmid et al. 2020 (Female) | USA | 1980 | NHS | 30–59 | Male & female (data shown for females) | SFFQ | >4 servings/week  Never (ref.) | 82348 | 4207 | 31.5 | 0.92 (0.79-1.08) | 0.41 | 8 | Height, BMI, BMI at age 18 (females) or 21 (males), race, physical activity, smoking status, history of hypertension, history of hypercholesterolemia, history of diabetes, family history of cancer, family history of diabetes, family history of myocardial infarction, current multivitamin use, regular aspirin use, menopausal status and hormone use (only for females), total caloric intake, alcohol consumption, glycemic load, intakes of unprocessed red meat, processed meat, nuts, fruits, vegetables, total calcium, total fiber | (22) |
| Schmid et al. 2020 (Male) | USA | 1980 | NHS | 40–79 | Male & female (data shown for males) | SFFQ | >4 servings/week  Never (ref.) | 40278 | 3733 | 25 | 1.1 (0.93-1.3) | 0.42 | 8 | Height, BMI, BMI at age 18 (females) or 21 (males), race, physical activity, smoking status, history of hypertension, history of hypercholesterolemia, history of diabetes, family history of cancer, family history of diabetes, family history of myocardial infarction, current multivitamin use, regular aspirin use, menopausal status and hormone use (only for females), total caloric intake, alcohol consumption, glycemic load, intakes of unprocessed red meat, processed meat, nuts, fruits, vegetables, total calcium, total fiber | (22) |
| Nakanishi et al. 2021 | Japan | 2009 | Yamagata Study | 40–74 | Male & female | FFQ | High (>1 times/day) None (<1 times/month) (ref.) | 14264 | 40 | 9 | 1.06 (0.39-2.84) | 0.91 | 7 | Age, sex, smoking status, alcohol consumption, BMI, hypertension, diabetes, education | (23) |
| Lin et al. 2022 | USA | 1999 | NHANES | > 18 | Male & female | 24-h recall | NR | 32625 | 651 | 8.1 | 0.68 (0.43-1.08) | 0.109 | 9 | Age, sex, race, BMI, white blood cell count, hemoglobin, platelet count, total bilirubin, creatinine, blood urea nitrogen, hypertension, diabetes, asthma congestive heart failure, coronary heart disease, stroke, chronic bronchitis, and cancer | (25) |
| Lu et al. 2022 (Male) | Japan | 1990 | Miyagi Cohort | 40–64 | Male & female (data shown for males) | FFQ | 3 times/week or almost daily  Almost never (ref.) | 16565 | 1048 | 25 | 0.99 (0.78-1.26) | 0.488 | 9 | Age (continuous), education level, BMI, smoking status, alcohol drinking status, history of hypertension, history of diabetes, energy intake, fish intake, vegetable and fruit intake | (26) |
| Lu et al. 2022 (Female) | Japan | 1990 | Miyagi Cohort | 40–64 | Male & female (data shown for females) | FFQ | 3 times/week or almost daily  Almost never (ref.) | 17596 | 645 | 25 | 0.87 (0.69-1.11) | 0.221 | 9 | Age (continuous), education level, BMI, smoking status, alcohol drinking status, history of hypertension, history of diabetes, energy intake, fish intake, vegetable and fruit intake | (26) |
| Zhang et al. 2023 | Swedish | 1991 | MDCS | 41–73 | Male & female | FFQ | Per 100 g/day increase | 20499 | 2531 | 21 | 0.95 (0.92-0.99) | 0.02 | 9 | Age, sex, dietary assessment version, season, total energy intake, leisure-time physical activity, smoking status, alcohol consumption, educational level, heredity score (including cancer, myocardial infarction, stroke, diabetes), diet quality index | (29) |
| Ge et al. 2023 (Female) | Japan | 1995 | JPHC | 40–69 | Male & female (data shown for females) | FFQ | Q4: 100.8 g/day (energy-adj.)  Q1: 0 g/day (ref.)* | 50193 | 2582 | 19.3 | 0.91 (0.81-1.02) | 0.32 | 7 | Age, study area, smoking status, alcohol frequency, BMI, physical activity, hypertension with medication, self, reported diabetes, green tea, coffee, energy-adjusted consumption of vegetables and fruits, total energy and total fat, menopausal status (only for females), exogenous hormone use (only for females), dairy intake | (27) |
| Ge et al. 2023 (Male) | Japan | 1995 | JPHC | 40–69 | Male & female (data shown for males) | FFQ | Q4: 76.6 g/day (energy-adj.)  Q1: 0 g/day (ref.)* | 43117 | 3379 | 19.3 | 0.9 (0.81-0.996) | 0.02 | 7 | Age, study area, smoking status, alcohol frequency, BMI, physical activity, hypertension with medication, self, reported diabetes, green tea, coffee, energy-adjusted consumption of vegetables and fruits, total energy and total fat, menopausal status (only for females), exogenous hormone use (only for females), dairy intake | (27) |
| Miyagawa et al. 2024 (Male) | Japan | 2005 | J-MICC | 35–69 | Male & female (data shown for males) | FFQ | T3: 40.2 g/day (energy-adj.)  T1: 0 g/day (ref.)* | 34118 | 307 | 12 | 0.96 (0.73-1.28) | 0.772 | 9 | Age, study site, history of hypertension, diabetes, dyslipidemia, BMI, smoking status, drinking status, physical activity, dietary intake of red meat, fish, vegetables, fruits | (28) |
| Miyagawa et al. 2024 (Female) | Japan | 2005 | J-MICC | 35–69 | Male & female (data shown for females) | FFQ | T3: 61.6 g/day (energy-adj.)  T1: 5.5 g/day (ref.)* | 45597 | 223 | 12 | 0.64 (0.46-0.9) | 0.007 | 9 | Age, study site, history of hypertension, diabetes, dyslipidemia, BMI, smoking status, drinking status, physical activity, dietary intake of red meat, fish, vegetables, fruits | (28) |
| **Fermented milks / Cancer** | | | | | | | | | | | | | | | |
| Khan et al. 2004 (Female) | Japan | 1984 | Hokkaido Study | 40–97 | Male & female (data shown for females) | FFQ | C5: several times per week, everyday  C1: never, several times per year, several times per month (ref.) | 1634 | 89 | 14.8 | 0.7 (0.4-1.3) |  | 7 | Age, health status, health education, health screening, smoking | (30) |
| Khan et al. 2004 (Male) | Japan | 1984 | Hokkaido Study | 40–97 | Male & female (data shown for males) | FFQ | C5: several times per week, everyday  C1: never, several times per year, several times per month (ref.) | 1524 | 155 | 13.8 | 0.8 (0.5-1.3) |  | 7 | Age, health status, health education, health screening, smoking | (30) |
| Matsumoto et al. 2007 | Japan | 1992 | JMS | 19–93 | Male & female | FFQ | Q5: almost everyday Q1: seldom (ref.) | 11606 | 255 | 9.15 | 1.48 (0.59-3.72) | 0.41 | 5 | Age, sex | (31) |
| Bonthuis et al. 2010 | Australia | 1992 | Nambour Skin Cancer Study | 25–78 | Male & female | FFQ | Q3: 76 g/day Q1: 0 g/day (ref.) | 1529 | 58 | 14.4 | There was no association between yoghurt intake and Cancer mortality (Data are not provided). (nan-nan) | | 8 | Age, sex, total energy intake, BMI, alcohol intake, school leaving age, physical activity level, pack-years of smoking, dietary supplement use, beta-carotene treatment during trial, presence of any medical condition, dietary calcium | (13) |
| Praagman et al. 2015 | Netherlands | 1993 | EPIC-NL | 20–70 | Male & female | FFQ | Q4: 144.5 g/day (energy-adj.)  Q1: 3.8 g/day (ref.)* | 34409 | 1216 | 15 | 1.02 (0.86-1.2) | 0.6 | 8 | Age, sex, smoking habit, BMI, physical activity, education level, hypertension at baseline, intakes of alcohol and energy-adjusted intakes of fruit and vegetables, total energy intake | (6) |
| Farvid et al. 2017 | Iran | 2004 | Golestan Study | 36–85 | Male & female | FFQ | Q5: 0.9 servings/day  Q1: 0.1 servings/day (ref.)** | 42403 | 859 | 8 | 0.86 (0.69-1.08) | 0.18 | 8 | Age, ethnicity, education, marital status, residency, smoking, opium use, alcohol use, BMI, systolic blood pressure, occupational physical activity, family history of cancer, wealth score, medication use, energy intake | (18) |
| Mazidi et al. 2019 | USA | 1999 | NHANES | > 20 | Male & female | 24-h recall | Q4: 3.08 cup eq servings/d Q1: 0.25 cup eq/day (ref.)* | 24474 | 827 | 6.4 | 1 (0.99-1.01) | 0.352 | 9 | Age, sex, race, education, marital status, poverty to income ratio, total energy intake, physical activity, smoking, alcohol consumption, carbohydrates, saturated fat, protein, dietary fiber, BMI, hypertension, diabetes | (21) |
| Pala et al. 2019 | Italy | 1993 | EPIC-Italy | 45–64 | Male & female | FFQ | Q4: >120 g/day  Q1 : 0 g/day (ref.) | 45009 | 1456 | 14.9 | 1 (0.83-1.2) | 0.82 | 8 | Region, sex, age, energy intake, weight, height, waist-to-hip ratio, alcohol consumption, smoking status, physical activity, relative index of inequality, Italian Mediterranean Index, intake of sugar | (20) |
| Schmid et al. 2020 (Female) | USA | 1980 | NHS | 30–59 | Male & female (data shown for females) | SFFQ | >4 servings/week  Never (ref.) | 82348 | 7985 | 31.5 | 0.87 (0.78-0.98) | 0.04 | 8 | Height, BMI, BMI at age 18 (females) or 21 (males), race, physical activity, smoking status, history of hypertension, history of hypercholesterolemia, history of diabetes, family history of cancer, family history of diabetes, family history of myocardial infarction, current multivitamin use, regular aspirin use, menopausal status and hormone use (only for females), total caloric intake, alcohol consumption, glycemic load, intakes of unprocessed red meat, processed meat, nuts, fruits, vegetables, total calcium, total fiber | (22) |
| Schmid et al. 2020 (Male) | USA | 1980 | NHS | 40–79 | Male & female (data shown for males) | SFFQ | >4 servings/week  Never (ref.) | 40278 | 4000 | 25 | 0.95 (0.79-1.13) | 0.19 | 8 | Height, BMI, BMI at age 18 (females) or 21 (males), race, physical activity, smoking status, history of hypertension, history of hypercholesterolemia, history of diabetes, family history of cancer, family history of diabetes, family history of myocardial infarction, current multivitamin use, regular aspirin use, menopausal status and hormone use (only for females), total caloric intake, alcohol consumption, glycemic load, intakes of unprocessed red meat, processed meat, nuts, fruits, vegetables, total calcium, total fiber | (22) |
| Nakanishi et al. 2021 | Japan | 2009 | Yamagata Study | 40–74 | Male & female | FFQ | High (>1 times/day) None (<1 times/month) (ref.) | 14264 | 90 | 9 | 0.53 (0.27-0.99) | 0.047 | 7 | Age, sex, smoking status, alcohol consumption, BMI, hypertension, diabetes, education | (23) |
| Lin et al. 2022 | USA | 1999 | NHANES | > 18 | Male & female | 24-h recall | NR | 32625 | 863 | 8.1 | 1 (0.72-1.38) | 0.972 | 9 | Age, sex, race, BMI, white blood cell count, hemoglobin, platelet count, total bilirubin, creatinine, blood urea nitrogen, hypertension, diabetes, asthma congestive heart failure, coronary heart disease, stroke, chronic bronchitis, and cancer | (25) |
| Lu et al. 2022 (Male) | Japan | 1990 | Miyagi Cohort | 40–64 | Male & female (data shown for males) | FFQ | 3 times/week or almost daily  Almost never (ref.) | 16565 | 1713 | 25 | 1.03 (0.85-1.24) | 0.791 | 9 | Age (continuous), education level, BMI, smoking status, alcohol drinking status, history of hypertension, history of diabetes, energy intake, fish intake, vegetable and fruit intake | (26) |
| Lu et al. 2022 (Female) | Japan | 1990 | Miyagi Cohort | 40–64 | Male & female (data shown for females) | FFQ | 3 times/week or almost daily  Almost never (ref.) | 17596 | 839 | 25 | 1.1 (0.89-1.34) | 0.541 | 9 | Age (continuous), education level, BMI, smoking status, alcohol drinking status, history of hypertension, history of diabetes, energy intake, fish intake, vegetable and fruit intake | (26) |
| Ge et al. 2023 (Male) | Japan | 1995 | JPHC | 40–69 | Male & female (data shown for males) | FFQ | Q4: 76.6 g/day (energy-adj.)  Q1: 0 g/day (ref.)* | 43117 | 5364 | 19.3 | 0.91 (0.84-0.99) | 0.04 | 7 | Age, study area, smoking status, alcohol frequency, BMI, physical activity, hypertension with medication, self, reported diabetes, green tea, coffee, energy-adjusted consumption of vegetables and fruits, total energy and total fat, menopausal status (only for females), exogenous hormone use (only for females), dairy intake | (27) |
| Ge et al. 2023 (Female) | Japan | 1995 | JPHC | 40–69 | Male & female (data shown for females) | FFQ | Q4: 100.8 g/day (energy-adj.)  Q1: 0 g/day (ref.)* | 50193 | 3076 | 19.3 | 0.92 (0.82-1.02) | 0.33 | 7 | Age, study area, smoking status, alcohol frequency, BMI, physical activity, hypertension with medication, self, reported diabetes, green tea, coffee, energy-adjusted consumption of vegetables and fruits, total energy and total fat, menopausal status (only for females), exogenous hormone use (only for females), dairy intake | (27) |
| Miyagawa et al. 2024 (Female) | Japan | 2005 | J-MICC | 35–69 | Male & female (data shown for females) | FFQ | T3: 61.6 g/day (energy-adj.)  T1: 5.5 g/day (ref.)* | 45597 | 725 | 12 | 1.03 (0.85-1.24) | 0.793 | 9 | Age, study site, history of hypertension, diabetes, dyslipidemia, BMI, smoking status, drinking status, physical activity, dietary intake of red meat, fish, vegetables, fruits | (28) |
| Miyagawa et al. 2024 (Male) | Japan | 2005 | J-MICC | 35–69 | Male & female (data shown for males) | FFQ | T3: 40.2 g/day (energy-adj.)  T1: 0 g/day (ref.)* | 34118 | 1363 | 12 | 0.93 (0.81-1.06) | 0.251 | 9 | Age, study site, history of hypertension, diabetes, dyslipidemia, BMI, smoking status, drinking status, physical activity, dietary intake of red meat, fish, vegetables, fruits | (28) |
| **Yogurt** | | | | | | | | | | | | | | | |
| **Yogurt / All-causes** | | | | | | | | | | | | | | | |
| Bonthuis et al. 2010 | Australia | 1992 | Nambour Skin Cancer Study | 25–78 | Male & female | FFQ | Q3: 76 g/day Q1: 0 g/day (ref.)* | 1529 | 177 | 14.4 | 1.22 (0.77-1.93) | 0.36 | 8 | Age, sex, total energy intake, BMI, alcohol intake, school leaving age, physical activity level, pack-years of smoking, dietary supplement use, beta-carotene treatment during trial, presence of any medical condition, dietary calcium | (13) |
| Sluik et al. 2014 | Multiple | 1992 | EPIC | 45–64 | Male & female | FFQ | Q3: 71 g/day (energy-adj.)  Q1: 3 g/day (ref.)* | 258911 | 12135 | 9.9 | 1 (1.0-1.0) | 0.21 | 8 | Age, region, sex, educational attainment, alcohol consumption, physical activity, smoking status and smoking intensity, factor loadings for the first three dietary patterns derived from factor analysis on 26 food groups | (15) |
| Praagman et al. 2015 | Netherlands | 1993 | EPIC-NL | 20–70 | Male & female | FFQ | Q4: 144.5 g/day (energy-adj.)  Q1: 3.8 g/day (ref.)* | 34409 | 2436 | 15 | 0.97 (0.86-1.09) | 0.9 | 8 | Age, sex, smoking habit, BMI, physical activity, education level, hypertension at baseline, intakes of alcohol and energy-adjusted intakes of fruit and vegetables, total energy intake | (6) |
| Farvid et al. 2017 | Iran | 2004 | Golestan Study | 36–85 | Male & female | FFQ | Q5: 0.9 servings/day  Q1: 0.1 servings/day (ref.)** | 42403 | 3291 | 8 | 0.89 (0.89-1.0) | 0.03 | 8 | Age, ethnicity, education, marital status, residency, smoking, opium use, alcohol use, BMI, systolic blood pressure, occupational physical activity, family history of cancer, wealth score, medication use, energy intake | (18) |
| Dehghan et al. 2018 | Multiple | 2003 | PURE | 35–70 | Male & female | FFQ | Q4: 1.5 servings/day Q1: 0 servings/day (ref.)* | 136384 | 6796 | 9.1 | 0.83 (0.69-0.99) | 0.0051 | 8 | Age, sex, education, urban or rural location, smoking, physical activity, history of diabetes, family history of cardiovascular disease, family history of cancer, quintiles of fruit, vegetable, red meat, starchy foods intake, total energy intake, centre was included as a random effect to account for clustering by location | (19) |
| Pala et al. 2019 | Italy | 1993 | EPIC-Italy | 45–64 | Male & female | FFQ | Q4: >120 g/day  Q1 : 0 g/day (ref.) | 45009 | 2468 | 14.9 | 0.95 (0.82-1.09) | 0.14 | 8 | Region, sex, age, energy intake, weight, height, waist-to-hip ratio, alcohol consumption, smoking status, physical activity, relative index of inequality, Italian Mediterranean Index, intake of sugar | (20) |
| Mazidi et al. 2019 | USA | 1999 | NHANES | > 20 | Male & female | 24-h recall | Q4: 3.08 cup eq servings/d Q1: 0.25 cup eq/day (ref.)* | 24474 | 3520 | 6.4 | 0.93 (0.85-1.01) | 0.523 | 9 | Age, sex, race, education, marital status, poverty to income ratio, total energy intake, physical activity, smoking, alcohol consumption, carbohydrates, saturated fat, protein, dietary fiber, BMI, hypertension, diabetes | (21) |
| Schmid et al. 2020 (Male) | USA | 1980 | NHS | 40–79 | Male & female (data shown for males) | SFFQ | >4 servings/week  Never (ref.) | 40278 | 12397 | 26 | 1.05 (0.95-1.16) | 0.7 | 8 | Height, BMI, BMI at age 18 (females) or 21 (males), race, physical activity, smoking status, history of hypertension, history of hypercholesterolemia, history of diabetes, family history of cancer, family history of diabetes, family history of myocardial infarction, current multivitamin use, regular aspirin use, menopausal status and hormone use (only for females), total caloric intake, alcohol consumption, glycemic load, intakes of unprocessed red meat, processed meat, nuts, fruits, vegetables, total calcium, total fiber | (22) |
| Schmid et al. 2020 (Female) | USA | 1980 | NHS | 30–59 | Male & female (data shown for females) | SFFQ | >4 servings/week  Never (ref.) | 82348 | 20831 | 32 | 0.91 (0.85-0.98) | 0.34 | 8 | Height, BMI, BMI at age 18 (females) or 21 (males), race, physical activity, smoking status, history of hypertension, history of hypercholesterolemia, history of diabetes, family history of cancer, family history of diabetes, family history of myocardial infarction, current multivitamin use, regular aspirin use, menopausal status and hormone use (only for females), total caloric intake, alcohol consumption, glycemic load, intakes of unprocessed red meat, processed meat, nuts, fruits, vegetables, total calcium, total fiber | (22) |
| Nakanishi et al. 2021 | Japan | 2009 | Yamagata Study | 40–74 | Male & female | FFQ | High (>1 times/day) None (<1 times/month) (ref.) | 14264 | 265 | 9 | 0.7 (0.49-0.99) | 0.04 | 7 | Age, sex, smoking status, alcohol consumption, BMI, hypertension, diabetes, education | (23) |
| Guo et al. 2022 | Denmark | 1982 | MONICA | 30–60 | Male & female | 7-day weighed food record | Q4: 194.7 g/week  Q1: 0 g/week** | 1746 | 660 | 30 | 1.05 (0.76-1.45) | 0.95 | 8 | Sex, BMI, food energy intake, alcohol consumption, education, smoking, physical activity, family history of myocardial infarction, multivitamin use, serum total cholesterol, serum triaclyglycerols, incidence of hypertension | (10) |
| Lin et al. 2022 | USA | 1999 | NHANES | > 18 | Male & female | 24-h recall | NR | 32625 | 3881 | 8.1 | 0.83 (0.71-0.98) | 0.035 | 9 | Age, sex, race, BMI, white blood cell count, hemoglobin, platelet count, total bilirubin, creatinine, blood urea nitrogen, hypertension, diabetes, asthma congestive heart failure, coronary heart disease, stroke, chronic bronchitis, and cancer | (25) |
| Lu et al. 2022 (Male) | Japan | 1990 | Miyagi Cohort | 40–64 | Male & female (data shown for males) | FFQ | 3 times/week or almost daily  Almost never (ref.) | 16565 | 4354 | 25 | 1.04 (0.92-1.17) | 0.253 | 9 | Age (continuous), education level, BMI, smoking status, alcohol drinking status, history of hypertension, history of diabetes, energy intake, fish intake, vegetable and fruit intake | (26) |
| Lu et al. 2022 (Female) | Japan | 1990 | Miyagi Cohort | 40–64 | Male & female (data shown for females) | FFQ | 3 times/week or almost daily  Almost never (ref.) | 17596 | 2522 | 25 | 0.92 (0.81-1.03) | 0.146 | 9 | Age (continuous), education level, BMI, smoking status, alcohol drinking status, history of hypertension, history of diabetes, energy intake, fish intake, vegetable and fruit intake | (26) |
| Miyagawa et al. 2024 (Male) | Japan | 2005 | J-MICC | 35–69 | Male & female (data shown for males) | FFQ | T3: 40.2 g/day (energy-adj.)  T1: 0 g/day (ref.)* | 34118 | 2379 | 12 | 0.9 (0.82-0.999) | 0.034 | 9 | Age, study site, history of cardiometabolic diseases, BMI, smoking status, drinking status, physical activity, dietary intake of red meat, fish, vegetables, fruits | (28) |
| Miyagawa et al. 2024 (Female) | Japan | 2005 | J-MICC | 35–69 | Male & female (data shown for females) | FFQ | T3: 61.6 g/day (energy-adj.)  T1: 5.5 g/day (ref.)* | 45597 | 1344 | 12 | 0.87 (0.76-0.997) | 0.046 | 9 | Age, study site, history of cardiometabolic diseases, BMI, smoking status, drinking status, physical activity, dietary intake of red meat, fish, vegetables, fruits | (28) |

| **Study & subgroup details** | **Region** | **Start year** | **Cohort** | **Age range at entry** | **Sex** | **Dietary assessm.** | **Exposure levels** | **No. of subjects** | **No. of deaths** | **Years of FU** | **HR (95% CI)** | **p-value** | **NOS** | **Adjustments** | **Ref.** |
| --- | --- | --- | --- | --- | --- | --- | --- | --- | --- | --- | --- | --- | --- | --- | --- |
| **Yogurt / CVD** | | | | | | | | | | | | | | | |
| Bonthuis et al. 2010 | Australia | 1992 | Nambour Skin Cancer Study | 25–78 | Male & female | FFQ | Q3: 76 g/day Q1: 0 g/day (ref.)* | 1529 | 61 | 14.4 | 0.65 (0.26-1.58) | 0.52 | 8 | Age, sex, total energy intake, BMI, alcohol intake, school leaving age, physical activity level, pack-years of smoking, dietary supplement use, beta-carotene treatment during trial, presence of any medical condition, dietary calcium | (13) |
| Praagman et al. 2015 | Netherlands | 1993 | EPIC-NL | 20–70 | Male & female | FFQ | Q4: 144.5 g/day (energy-adj.)  Q1: 3.8 g/day (ref.)* | 34409 | 727 | 15 | 0.98 (0.79-1.22) | 0.9 | 8 | Age, sex, smoking habit, BMI, physical activity, education level, hypertension at baseline, intakes of alcohol and energy-adjusted intakes of fruit and vegetables, total energy intake | (6) |
| Praagman et al. 2015 (Stroke M.) | Netherlands | 1990 | Rotterdam Study | ≥ 55 | Male & female | SFFQ | T3: >100 g/day  T1: <50 g/day (ref.)* | 4235 | 182 | 17.3 | 1.01 (0.71-1.44) | 0.93 | 8 | Age, sex, total energy intake, BMI, smoking, education level, alcohol intake, intakes of vegetables, fruit, meat, bread, fish coffee, tea | (11) |
| Praagman et al. 2015 (CHD M.) | Netherlands | 1990 | Rotterdam Study | ≥ 55 | Male & female | SFFQ | T3: >100 g/day  T1: <50 g/day (ref.)* | 4235 | 350 | 17.3 | 0.98 (0.76-1.26) | 0.84 | 8 | Age, sex, total energy intake, BMI, smoking, education level, alcohol intake, intakes of vegetables, fruit, meat, bread, fish coffee, tea | (11) |
| Farvid et al. 2017 | Iran | 2004 | Golestan Study | 36–85 | Male & female | FFQ | Q5: 0.9 servings/day  Q1: 0.1 servings/day (ref.)** | 42403 | 1467 | 8 | 0.84 (0.7-1.0) | 0.03 | 8 | Age, ethnicity, education, marital status, residency, smoking, opium use, alcohol use, BMI, systolic blood pressure, occupational physical activity, family history of cancer, wealth score, medication use, energy intake | (18) |
| Pala et al. 2019 | Italy | 1993 | EPIC-Italy | 45–64 | Male & female | FFQ | Q4: >120 g/day  Q1 : 0 g/day (ref.) | 45009 | 249 | 14.9 | 0.85 (0.59-1.23) | 0.15 | 8 | Region, sex, age, energy intake, weight, height, waist-to-hip ratio, alcohol consumption, smoking status, physical activity, relative index of inequality, Italian Mediterranean Index, intake of sugar | (20) |
| Mazidi et al. 2019 | USA | 1999 | NHANES | > 20 | Male & female | 24-h recall | Q4: 3.08 cup eq servings/d Q1: 0.25 cup eq/day (ref.)* | 24474 | 709 | 6.4 | 0.98 (0.97-0.99) | 0.125 | 9 | Age, sex, race, education, marital status, poverty to income ratio, total energy intake, physical activity, smoking, alcohol consumption, carbohydrates, saturated fat, protein, dietary fiber, BMI, hypertension, diabetes | (21) |
| Schmid et al. 2020 (Male) | USA | 1980 | NHS | 40–79 | Male & female (data shown for males) | SFFQ | >4 servings/week  Never (ref.) | 40278 | 3733 | 25 | 1.1 (0.93-1.3) | 0.42 | 8 | Height, BMI, BMI at age 18 (females) or 21 (males), race, physical activity, smoking status, history of hypertension, history of hypercholesterolemia, history of diabetes, family history of cancer, family history of diabetes, family history of myocardial infarction, current multivitamin use, regular aspirin use, menopausal status and hormone use (only for females), total caloric intake, alcohol consumption, glycemic load, intakes of unprocessed red meat, processed meat, nuts, fruits, vegetables, total calcium, total fiber | (22) |
| Schmid et al. 2020 (Female) | USA | 1980 | NHS | 30–59 | Male & female (data shown for females) | SFFQ | >4 servings/week  Never (ref.) | 82348 | 4207 | 31.5 | 0.92 (0.79-1.08) | 0.41 | 8 | Height, BMI, BMI at age 18 (females) or 21 (males), race, physical activity, smoking status, history of hypertension, history of hypercholesterolemia, history of diabetes, family history of cancer, family history of diabetes, family history of myocardial infarction, current multivitamin use, regular aspirin use, menopausal status and hormone use (only for females), total caloric intake, alcohol consumption, glycemic load, intakes of unprocessed red meat, processed meat, nuts, fruits, vegetables, total calcium, total fiber | (22) |
| Nakanishi et al. 2021 | Japan | 2009 | Yamagata Study | 40–74 | Male & female | FFQ | High (>1 times/day) None (<1 times/month) (ref.) | 14264 | 40 | 9 | 1.06 (0.39-2.84) | 0.91 | 7 | Age, sex, smoking status, alcohol consumption, BMI, hypertension, diabetes, education | (23) |
| Lin et al. 2022 | USA | 1999 | NHANES | > 18 | Male & female | 24-h recall | NR | 32625 | 651 | 8.1 | 0.68 (0.43-1.08) | 0.109 | 9 | Age, sex, race, BMI, white blood cell count, hemoglobin, platelet count, total bilirubin, creatinine, blood urea nitrogen, hypertension, diabetes, asthma congestive heart failure, coronary heart disease, stroke, chronic bronchitis, and cancer | (25) |
| Lu et al. 2022 (Male) | Japan | 1990 | Miyagi Cohort | 40–64 | Male & female (data shown for males) | FFQ | 3 times/week or almost daily  Almost never (ref.) | 16565 | 1048 | 25 | 0.99 (0.78-1.26) | 0.488 | 9 | Age (continuous), education level, BMI, smoking status, alcohol drinking status, history of hypertension, history of diabetes, energy intake, fish intake, vegetable and fruit intake | (26) |
| Lu et al. 2022 (Female) | Japan | 1990 | Miyagi Cohort | 40–64 | Male & female (data shown for females) | FFQ | 3 times/week or almost daily  Almost never (ref.) | 17596 | 645 | 25 | 0.87 (0.69-1.11) | 0.221 | 9 | Age (continuous), education level, BMI, smoking status, alcohol drinking status, history of hypertension, history of diabetes, energy intake, fish intake, vegetable and fruit intake | (26) |
| Miyagawa et al. 2024 (Male) | Japan | 2005 | J-MICC | 35–69 | Male & female (data shown for males) | FFQ | T3: 40.2 g/day (energy-adj.)  T1: 0 g/day (ref.)* | 34118 | 307 | 12 | 0.96 (0.73-1.28) | 0.772 | 9 | Age, study site, history of hypertension, diabetes, dyslipidemia, BMI, smoking status, drinking status, physical activity, dietary intake of red meat, fish, vegetables, fruits | (28) |
| Miyagawa et al. 2024 (Female) | Japan | 2005 | J-MICC | 35–69 | Male & female (data shown for females) | FFQ | T3: 61.6 g/day (energy-adj.)  T1: 5.5 g/day (ref.)* | 45597 | 223 | 12 | 0.64 (0.46-0.9) | 0.007 | 9 | Age, study site, history of hypertension, diabetes, dyslipidemia, BMI, smoking status, drinking status, physical activity, dietary intake of red meat, fish, vegetables, fruits | (28) |
| **Yogurt / Cancer** | | | | | | | | | | | | | | | |
| Khan et al. 2004 (Male) | Japan | 1984 | Hokkaido Study | 40–97 | Male & female (data shown for males) | FFQ | C5: several times per week, everyday  C1: never, several times per year, several times per month (ref.) | 1524 | 155 | 13.8 | 0.8 (0.5-1.3) |  | 7 | Age, health status, health education, health screening, smoking | (30) |
| Khan et al. 2004 (Female) | Japan | 1984 | Hokkaido Study | 40–97 | Male & female (data shown for females) | FFQ | C5: several times per week, everyday  C1: never, several times per year, several times per month (ref.) | 1634 | 89 | 14.8 | 0.7 (0.4-1.3) |  | 7 | Age, health status, health education, health screening, smoking | (30) |
| Matsumoto et al. 2007 | Japan | 1992 | JMS | 19–93 | Male & female | FFQ | Q5: almost everyday Q1: seldom (ref.) | 11606 | 255 | 9.15 | 1.48 (0.59-3.72) | 0.41 | 5 | Age, sex | (31) |
| Bonthuis et al. 2010 | Australia | 1992 | Nambour Skin Cancer Study | 25–78 | Male & female | FFQ | Q3: 76 g/day Q1: 0 g/day (ref.) | 1529 | 58 | 14.4 | There was no association between yoghurt intake and Cancer mortality (Data are not provided). (nan-nan) | | 8 | Age, sex, total energy intake, BMI, alcohol intake, school leaving age, physical activity level, pack-years of smoking, dietary supplement use, beta-carotene treatment during trial, presence of any medical condition, dietary calcium | (13) |
| Praagman et al. 2015 | Netherlands | 1993 | EPIC-NL | 20–70 | Male & female | FFQ | Q4: 144.5 g/day (energy-adj.)  Q1: 3.8 g/day (ref.)* | 34409 | 1216 | 15 | 1.02 (0.86-1.2) | 0.6 | 8 | Age, sex, smoking habit, BMI, physical activity, education level, hypertension at baseline, intakes of alcohol and energy-adjusted intakes of fruit and vegetables, total energy intake | (6) |
| Farvid et al. 2017 | Iran | 2004 | Golestan Study | 36–85 | Male & female | FFQ | Q5: 0.9 servings/day  Q1: 0.1 servings/day (ref.)** | 42403 | 859 | 8 | 0.86 (0.69-1.08) | 0.18 | 8 | Age, ethnicity, education, marital status, residency, smoking, opium use, alcohol use, BMI, systolic blood pressure, occupational physical activity, family history of cancer, wealth score, medication use, energy intake | (18) |
| Pala et al. 2019 | Italy | 1993 | EPIC-Italy | 45–64 | Male & female | FFQ | Q4: >120 g/day  Q1 : 0 g/day (ref.) | 45009 | 1456 | 14.9 | 1 (0.83-1.2) | 0.82 | 8 | Region, sex, age, energy intake, weight, height, waist-to-hip ratio, alcohol consumption, smoking status, physical activity, relative index of inequality, Italian Mediterranean Index, intake of sugar | (20) |
| Mazidi et al. 2019 | USA | 1999 | NHANES | > 20 | Male & female | 24-h recall | Q4: 3.08 cup eq servings/d Q1: 0.25 cup eq/day (ref.)* | 24474 | 827 | 6.4 | 1 (0.99-1.01) | 0.352 | 9 | Age, sex, race, education, marital status, poverty to income ratio, total energy intake, physical activity, smoking, alcohol consumption, carbohydrates, saturated fat, protein, dietary fiber, BMI, hypertension, diabetes | (21) |
| Schmid et al. 2020 (Male) | USA | 1980 | NHS | 40–79 | Male & female (data shown for males) | SFFQ | >4 servings/week  Never (ref.) | 40278 | 4000 | 25 | 0.95 (0.79-1.13) | 0.19 | 8 | Height, BMI, BMI at age 18 (females) or 21 (males), race, physical activity, smoking status, history of hypertension, history of hypercholesterolemia, history of diabetes, family history of cancer, family history of diabetes, family history of myocardial infarction, current multivitamin use, regular aspirin use, menopausal status and hormone use (only for females), total caloric intake, alcohol consumption, glycemic load, intakes of unprocessed red meat, processed meat, nuts, fruits, vegetables, total calcium, total fiber | (22) |
| Schmid et al. 2020 (Female) | USA | 1980 | NHS | 30–59 | Male & female (data shown for females) | SFFQ | >4 servings/week  Never (ref.) | 82348 | 7985 | 31.5 | 0.87 (0.78-0.98) | 0.04 | 8 | Height, BMI, BMI at age 18 (females) or 21 (males), race, physical activity, smoking status, history of hypertension, history of hypercholesterolemia, history of diabetes, family history of cancer, family history of diabetes, family history of myocardial infarction, current multivitamin use, regular aspirin use, menopausal status and hormone use (only for females), total caloric intake, alcohol consumption, glycemic load, intakes of unprocessed red meat, processed meat, nuts, fruits, vegetables, total calcium, total fiber | (22) |
| Nakanishi et al. 2021 | Japan | 2009 | Yamagata Study | 40–74 | Male & female | FFQ | High (>1 times/day) None (<1 times/month) (ref.) | 14264 | 90 | 9 | 0.53 (0.27-0.99) | 0.047 | 7 | Age, sex, smoking status, alcohol consumption, BMI, hypertension, diabetes, education | (23) |
| Lin et al. 2022 | USA | 1999 | NHANES | > 18 | Male & female | 24-h recall | NR | 32625 | 863 | 8.1 | 1 (0.72-1.38) | 0.972 | 9 | Age, sex, race, BMI, white blood cell count, hemoglobin, platelet count, total bilirubin, creatinine, blood urea nitrogen, hypertension, diabetes, asthma congestive heart failure, coronary heart disease, stroke, chronic bronchitis, and cancer | (25) |
| Lu et al. 2022 (Male) | Japan | 1990 | Miyagi Cohort | 40–64 | Male & female (data shown for males) | FFQ | 3 times/week or almost daily  Almost never (ref.) | 16565 | 1713 | 25 | 1.03 (0.85-1.24) | 0.791 | 9 | Age (continuous), education level, BMI, smoking status, alcohol drinking status, history of hypertension, history of diabetes, energy intake, fish intake, vegetable and fruit intake | (26) |
| Lu et al. 2022 (Female) | Japan | 1990 | Miyagi Cohort | 40–64 | Male & female (data shown for females) | FFQ | 3 times/week or almost daily  Almost never (ref.) | 17596 | 839 | 25 | 1.1 (0.89-1.34) | 0.541 | 9 | Age (continuous), education level, BMI, smoking status, alcohol drinking status, history of hypertension, history of diabetes, energy intake, fish intake, vegetable and fruit intake | (26) |
| Miyagawa et al. 2024 (Male) | Japan | 2005 | J-MICC | 35–69 | Male & female (data shown for males) | FFQ | T3: 40.2 g/day (energy-adj.)  T1: 0 g/day (ref.)* | 34118 | 1363 | 12 | 0.93 (0.81-1.06) | 0.251 | 9 | Age, study site, history of hypertension, diabetes, dyslipidemia, BMI, smoking status, drinking status, physical activity, dietary intake of red meat, fish, vegetables, fruits | (28) |
| Miyagawa et al. 2024 (Female) | Japan | 2005 | J-MICC | 35–69 | Male & female (data shown for females) | FFQ | T3: 61.6 g/day (energy-adj.)  T1: 5.5 g/day (ref.)* | 45597 | 725 | 12 | 1.03 (0.85-1.24) | 0.793 | 9 | Age, study site, history of hypertension, diabetes, dyslipidemia, BMI, smoking status, drinking status, physical activity, dietary intake of red meat, fish, vegetables, fruits | (28) |
| **Yogurt / GI cancer** | | | | | | | | | | | | | | | |
| Kojima et al. 2004 (Male Colon CA) | Japan | 1988 | JACC | 40–79 | Male & female (data shown for males) | FFQ | 1–7 times/week  Seldom (ref.) | 45181 | 138 | 9.9 | 0.8 (0.42-1.51) | 0.37 | 8 | Age, family history of colorectal cancer, BMI, frequency of alcohol intake, current smoking status, walking time per day, educational level | (32) |
| Kojima et al. 2004 (Female Colon CA) | Japan | 1988 | JACC | 40–79 | Male & female (data shown for females) | FFQ | 1–7 times/week  Seldom (ref.) | 62643 | 146 | 9.9 | 0.97 (0.61-1.56) | 0.93 | 8 | Age, family history of colorectal cancer, BMI, frequency of alcohol intake, current smoking status, walking time per day, educational level | (32) |
| Kojima et al. 2004 (Male Rectal CA) | Japan | 1988 | JACC | 40–79 | Male & female (data shown for males) | FFQ | 1–7 times/week  Seldom (ref.) | 45181 | 116 | 9.9 | 0.46 (0.21-1.02) | 0.04 | 8 | Age, family history of colorectal cancer, BMI, frequency of alcohol intake, current smoking status, walking time per day, educational level | (32) |
| Kojima et al. 2004 (Female Rectal CA) | Japan | 1988 | JACC | 40–79 | Male & female (data shown for females) | FFQ | 1–7 times/week  Seldom (ref.) | 62643 | 57 | 9.9 | 1.51 (0.6-3.8) | 0.14 | 8 | Age, family history of colorectal cancer, BMI, frequency of alcohol intake, current smoking status, walking time per day, educational level | (32) |
| Khan et al. 2004 (Male Stomach CA) | Japan | 1984 | Hokkaido Study | 40–97 | Male & female (data shown for males) | FFQ | C5: several times per week, everyday  C1: never, several times per year, several times per month (ref.) | 1524 | 36 | 13.8 | 1.6 (0.8-3.6) |  | 7 | Age, health status, health education, health screening, smoking | (30) |
| Khan et al. 2004 (Female Stomach CA) | Japan | 1984 | Hokkaido Study | 40–97 | Male & female (data shown for females) | FFQ | C5: several times per week, everyday  C1: never, several times per year, several times per month (ref.) | 1634 | 15 | 14.8 | 0.3 (0.0-2.3) |  | 7 | Age, health status, health education, health screening, smoking | (30) |
| Khan et al. 2004 (Male Pancreatic CA) | Japan | 1984 | Hokkaido Study | 40–97 | Male & female (data shown for males) | FFQ | C5: several times per week, everyday  C1: never, several times per year, several times per month (ref.) | 1524 | 12 | 13.8 | 0.5 (0.1-4.2) |  | 7 | Age, health status, health education, health screening, smoking | (30) |
| Khan et al. 2004 (Female Pancreatic CA) | Japan | 1984 | Hokkaido Study | 40–97 | Male & female (data shown for females) | FFQ | C5: several times per week, everyday  C1: never, several times per year, several times per month (ref.) | 1634 | 13 | 14.8 | 0.9 (0.2-3.9) |  | 7 | Age, health status, health education, health screening, smoking | (30) |
| Khan et al. 2004 (Male Colorectal CA) | Japan | 1984 | Hokkaido Study | 40–97 | Male & female (data shown for males) | FFQ | C5: several times per week, everyday  C1: never, several times per year, several times per month (ref.) | 1524 | 15 | 13.8 | 0.7 (0.3-2.0) |  | 7 | Age, health status, health education, health screening, smoking | (30) |
| Khan et al. 2004 (Female Colorectal CA) | Japan | 1984 | Hokkaido Study | 40–97 | Male & female (data shown for females) | FFQ | C5: several times per week, everyday  C1: never, several times per year, several times per month (ref.) | 1634 | 14 | 14.8 | 0.4 (0.1-2.8) |  | 7 | Age, health status, health education, health screening, smoking | (30) |
| Matsumoto et al. 2007 (Colon CA) | Japan | 1992 | JMS | 19–93 | Male & female | FFQ | Q5: almost everyday Q1: seldom (ref.) | 11606 | 25 | 9.15 | 1.28 (0.3-5.48) | 0.74 | 5 | Age, sex | (31) |
| Matsumoto et al. 2007 (Stomach CA) | Japan | 1992 | JMS | 19–93 | Male & female | FFQ | Q5: almost everyday Q1: seldom (ref.) | 11606 | 32 | 9.15 | 0.47 (0.06-3.46) | 0.46 | 5 | Age, sex | (31) |
| Matsumoto et al. 2007 (Bile duct CA) | Japan | 1992 | JMS | 19–93 | Male & female | FFQ | Q5: almost everyday Q1: seldom (ref.) | 11606 | 13 | 9.15 | 1.17 (0.15-9.1) | 0.88 | 5 | Age, sex | (31) |
| Matsumoto et al. 2007 (Pancreatic CA) | Japan | 1992 | JMS | 19–93 | Male & female | FFQ | Q5: almost everyday Q1: seldom (ref.) | 11606 | 10 | 9.15 | 2.77 (0.58-13.3) | 0.2 | 5 | Age, sex | (31) |
| Tokui et al. 2022 (Male Stomach CA) | Japan | 1988 | JACC | 40–79 | Male & female (data shown for males) | FFQ | >1 times/day  None (ref.) | 46465 | 574 | 9.9 | 0.82 (0.5-1.37) | 0.47 | 6 | Age | (33) |
| Tokui et al. 2022 (Female Stomach CA) | Japan | 1988 | JACC | 40–79 | Male & female (data shown for females) | FFQ | >1 times/day  None (ref.) | 64327 | 285 | 9.9 | 0.88 (0.47-1.64) | 0.93 | 6 | Age | (33) |
| **Yogurt / Lung cancer** | | | | | | | | | | | | | | | |
| Ozasa et al. 2001 (Male) | Japan | 1988 | JACC | 40–79 | Male & female (data shown for males) | FFQ | Q3: >3 dishes/day Q1: <1 dishes/day (ref.) | 42940 | 446 | 7.7 | 0.81 (0.54-1.22) | 0.14 | 8 | Age, parents' history of lung cancer, smoking status, smoking index, time since quitting smoking | (34) |
| Ozasa et al. 2001 (Female) | Japan | 1988 | JACC | 40–79 | Male & female (data shown for females) | FFQ | Q3: >3 dishes/day Q1: <1 dishes/day (ref.) | 55308 | 126 | 7.7 | 0.82 (0.45-1.52) | 0.45 | 8 | Age, parents' history of lung cancer, smoking status, smoking index, time since quitting smoking | (34) |
| Khan et al. 2004 (Male) | Japan | 1984 | Hokkaido Study | 40–97 | Male & female (data shown for males) | FFQ | C5: several times per week, everyday  C1: never, several times per year, several times per month (ref.) | 1524 | 41 | 13.8 | 1.3 (0.6-2.8) |  | 7 | Age, health status, health education, health screening, smoking | (30) |
| Khan et al. 2004 (Female) | Japan | 1984 | Hokkaido Study | 40–97 | Male & female (data shown for females) | FFQ | C5: several times per week, everyday  C1: never, several times per year, several times per month (ref.) | 1634 | 10 | 14.8 | 0.5 (0.1-4.0) |  | 7 | Age, health status, health education, health screening, smoking | (30) |
| Matsumoto et al. 2007 | Japan | 1992 | JMS | 19–93 | Male & female | FFQ | Q5: almost everyday Q1: seldom (ref.) | 11606 | 56 | 9.15 | 0.95 (0.29-3.03) | 0.92 | 5 | Age, sex | (31) |
| **Yogurt / Reproductive cancer** | | | | | | | | | | | | | | | |
| Park et al. 2007 | USA | 1995 | NIH-AARP | 50–71 | Male | FFQ | C5: > 3 servings/day  C1: < 0.5 servings/day (ref.) | 293888 | 178 | 6 | 0.78 (0.25-2.5) | 0.68 | 7 | Age, race/ethnicity, education, marital status, BMI, physical activity, smoking, alcohol consumption, history of diabetes, family history of prostate cancer, PSA screening, dietary calcium, energy intake | (35) |
| Sakauchi et al. 2007 | Japan | 1988 | JACC | 40–79 | Female | FFQ | Q3: ≥1–2 times/week Q1: seldom (ref.) | 63541 | 77 | 13.3 | 1.66 (0.71-3.91) | 0.24 | 7 | Age, menopausal status, number of pregnancies, history of sex hormone use, BMI, physical activity, education | (36) |
| **Cheese** | | | | | | | | | | | | | | | |
| **Cheese / All-causes** | | | | | | | | | | | | | | | |
| Mann et al. 1997 | UK | 1980 | UK Health-Conscious Diet Cohort | 16–79 | Male & female | SFFQ | C3: ≥ 5 times/week C1: < 1 times/week (ref.) | 10802 | 383 | 13.3 | 1.02 (0.76-1.37) |  | 6 | Age, sex, smoking, social class | (37) |
| Fortes et al. 2000 | Italy | 1993 | Rome Elderly Cohort | ≥ 65 | Male & female | FFQ | T3: > 3 times/week  T1: <1 times/week (ref.) | 161 | 53 | 5 | 1.3 (0.51-3.34) |  | 7 | No adjustments | (38) |
| Bonthuis et al. 2010 | Australia | 1992 | Nambour Skin Cancer Study | 25–78 | Male & female | FFQ | Q3: 30 g/day  Q1: 4 g/day (ref.)* | 1529 | 177 | 14.4 | 0.91 (0.57-1.45) | 0.56 | 8 | Age, sex, total energy intake, BMI, alcohol intake, school leaving age, physical activity level, pack-years of smoking, dietary supplement use, beta-carotene treatment during trial, presence of any medical condition, dietary calcium | (13) |
| Goldbohm et al. 2011 (Female) | Netherlands | 1986 | NLCS | 55–69 | Male & female (data shown for females) | FFQ | Q5/C5: 56 g/day  Q1/C1: 1 g/day (ref.)* | 62573 | 5478 | 10 | 0.98 (0.88-1.1) | 0.607 | 8 | Age, education, smoking, physical activity, BMI, multivitamin use, alcohol, energy, energy-adjusted mono- and polyunsaturated fat intakes, vegetable and fruit consumption | (14) |
| Goldbohm et al. 2011 (Male) | Netherlands | 1986 | NLCS | 55–69 | Male & female (data shown for males) | FFQ | Q5/C5: 56 g/day  Q1/C1: 1 g/day (ref.)* | 58279 | 10658 | 10 | 1.04 (0.96-1.12) | 0.083 | 8 | Age, education, smoking, physical activity, BMI, multivitamin use, alcohol, energy, energy-adjusted mono- and polyunsaturated fat intakes, vegetable and fruit consumption | (14) |
| van et al. Aerde 2013 | Netherlands | 1989 | The Hoorn Study | 50–75 | Male & female | FFQ | Q4: ≥14.7 g/day Q1: ≤6.0 g/day (ref.)* | 1956 | 403 | 12.4 | 0.96 (0.84-1.09) | 0.51 | 8 | Age, sex, BMI, smoking, educational level, total energy intake, alcohol consumption, physical activity, intake of meat, fish, bread, vegetables, fruit, coffee, tea | (8) |
| Sluik et al. 2014 | Multiple | 1992 | EPIC | 45–64 | Male & female | FFQ | Q3: 39 g/day (energy-adj.)  Q1: 24 g/day (ref.)* | 258911 | 12135 | 9.9 | 0.96 (0.95-0.98) | 0.23 | 8 | Age, region, sex, educational attainment, alcohol consumption, physical activity, smoking status and smoking intensity, factor loadings for the first three dietary patterns derived from factor analysis on 26 food groups | (15) |
| Praagman et al. 2015 | Netherlands | 1993 | EPIC-NL | 20–70 | Male & female | FFQ | Q4: 53.2 g/day (energy-adj.)  Q1: 6.6 g/day (ref.)* | 34409 | 2436 | 15 | 1 (0.89-1.12) | 0.9 | 8 | Age, sex, smoking habit, BMI, physical activity, education level, hypertension at baseline, intakes of alcohol and energy-adjusted intakes of fruit and vegetables, total energy intake | (6) |
| Bongard et al. 2016 | France | 1995 | MONICA | 45–64 | Male | 3-day food record | Q4: 283 g/day (energy-adj.)  Q1: 0 g/day (ref.)* | 960 | 150 | 14.8 | 1.16 (0.77-1.77) | 0.68 | 8 | Center, age, payment of income tax, obesity, alcohol consumption, smoking habits, physical activity, presence of a serious chronic condition, diet quality score | (16) |
| Farvid et al. 2017 | Iran | 2004 | Golestan Study | 36–85 | Male & female | FFQ | Q5: 0.8 servings/day  Q1: 0 servings/day (ref.)** | 42403 | 3291 | 8 | 0.84 (0.73-0.96) | 0.02 | 8 | Age, ethnicity, education, marital status, residency, smoking, opium use, alcohol use, BMI, systolic blood pressure, occupational physical activity, family history of cancer, wealth score, medication use, energy intake | (18) |
| Tognon et al. 2017 | Sweden | 1986 | NSHDS | 24–74 | Male & female | FFQ | Q4: ≥2.5 times/day (energy-adj.)  Q1: <1 times/week (ref.) | 103256 | 6892 | 13.7 | 0.94 (0.91-0.97) | 0.001 | 8 | Age, sex, BMI, screening year, smoking, education, energy intake | (17) |
| Dehghan et al. 2018 | Multiple | 2003 | PURE | 35–70 | Male & female | FFQ | Q4: 1.7 servings/day Q1: 0 servings/day (ref.)* | 136384 | 6796 | 9.1 | 0.87 (0.72-1.05) | 0.2383 | 8 | Age, sex, education, urban or rural location, smoking, physical activity, history of diabetes, family history of cardiovascular disease, family history of cancer, quintiles of fruit, vegetable, red meat, starchy foods intake, total energy intake, centre was included as a random effect to account for clustering by location | (19) |
| Tognon et al. 2018 | Sweden | 1971 | H70 | 70 | Male & female | Interview questionnaire | T3: >45 g/day (males and females)  T1: <28.7 (males) and <21.4 (females) g/day (ref.) | 1213 | 833 | 13.2 | 0.71 (0.48-1.04) |  | 9 | Sex, birth cohort (included as a stratification variable), smoking status, BMI, education, marital status, physical activity, total energy intake | (39) |
| Virtanen et al. 2019 | Finland | 1984 | KIHD | 42–60 | Male | 4-day food record | Q4: 50 g/day (energy-adj.)  Q1: 0 g/day (ref.)* | 2641 | 1225 | 22.3 | 0.99 (0.83-1.17) | 0.91 | 8 | Age, examination year, energy intake, income, education years, marital status, leisure-time physical activity, pack-years of smoking, alcohol intake, BMI, diagnosis of type 2 diabetes, cardiovascular disease, cancer, or hypertension or use of cardiac, hypercholesterolemia, hypertension, or diabetes medications, intakes of fiber and saturated, monounsaturated, polyunsaturated, trans fatty acids | (9) |
| Ding et al. 2019 (NHS) | USA | 1976 | NHS, NHS II, HPFS | 30–55 | Male & female (data shown for females) | FFQ | C4: > 32.7 g/day C1: < 30 g/week (ref.) | 74805 | 25182 | 40 | 1.09 (1.0-1.18) | 0.07 | 8 | Age, family history of CVD, history of cancer, family history of cardiovascular disease, baseline disease status (hypertension, hypercholesterolemia), baseline BMI, physical activity, alternate healthy eating index score, total energy intake, smoking status, alcohol consumption, postmenopausal status (only for females), current postmenopausal hormone use (only for females) | (40) |
| Ding et al. 2019 (HPFS) | USA | 1986 | NHS, NHS II, HPFS | 40–75 | Male & female (data shown for males) | FFQ | C4: > 27.6 g/day C1: < 30 g/week (ref.) | 49602 | 23560 | 30 | 0.92 (0.85-0.99) | 0.04 | 8 | Age, family history of CVD, history of cancer, family history of cardiovascular disease, baseline disease status (hypertension, hypercholesterolemia), baseline BMI, physical activity, alternate healthy eating index score, total energy intake, smoking status, alcohol consumption, postmenopausal status (only for females), current postmenopausal hormone use (only for females) | (40) |
| Ding et al. 2019 (NHSII) | USA | 1989 | NHS, NHS II, HPFS | 25–42 | Male & female (data shown for females) | FFQ | C4: > 36 g/day C1: < 30 g/week (ref.) | 93348 | 2696 | 27 | 1.2 (0.96-1.51) | 0.009 | 8 | Age, family history of CVD, history of cancer, family history of cardiovascular disease, baseline disease status (hypertension, hypercholesterolemia), baseline BMI, physical activity, alternate healthy eating index score, total energy intake, smoking status, alcohol consumption, postmenopausal status (only for females), current postmenopausal hormone use (only for females) | (40) |
| Pala et al. 2019 | Italy | 1993 | EPIC-Italy | 45–64 | Male & female | FFQ | Q4: >100 g/day  Q1: 0 to ≤28 g/day (ref.) | 45009 | 2468 | 14.9 | 0.99 (0.84-1.16) | 0.72 | 8 | Region, sex, age, energy intake, weight, height, waist-to-hip ratio, alcohol consumption, smoking status, physical activity, relative index of inequality, Italian Mediterranean Index, intake of sugar | (20) |
| Mazidi et al. 2019 | USA | 1999 | NHANES | > 20 | Male & female | 24-h recall | Q4: 4.6 oz natural cheese or 6.2 oz processed cheese Q1: 0.38 oz natural cheese or 0.5 oz processed cheese* | 24474 | 3520 | 6.4 | 0.92 (0.87-0.97) | 0.001 | 9 | Age, sex, race, education, marital status, poverty to income ratio, total energy intake, physical activity, smoking, alcohol consumption, carbohydrates, saturated fat, protein, dietary fiber, BMI, hypertension, diabetes | (21) |
| Sonestedt et al. 2021 | Sweden | 1991 | MDCS | 45–73 | Male & female | SFFQ, food record, and interview | C6: >100 g/day C1: 0-20 g/day (ref.) | 26190 | 7156 | 19 | 0.83 (0.72-0.95) | 0.001 | 9 | Age, sex, diet assessment method, season, energy, BMI, education, physical activity, smoking, alcohol habits, diet (fruit and vegetables, meat, fiber, sugar-sweetened beverages) | (24) |
| Guo et al. 2022 | Denmark | 1982 | MONICA | 30–60 | Male & female | 7-day weighed food record | Q4: 73.7 g/week  Q1: 6.3 g/week** | 1746 | 660 | 30 | 0.96 (0.76-1.22) | 0.46 | 8 | Sex, BMI, food energy intake, alcohol consumption, education, smoking, physical activity, family history of myocardial infarction, multivitamin use, serum total cholesterol, serum triaclyglycerols, incidence of hypertension | (10) |
| Lu et al. 2022 (Male) | Japan | 1990 | Miyagi Cohort | 40–64 | Male & female (data shown for males) | FFQ | 3 times/week or almost daily  Almost never (ref.) | 16565 | 4354 | 25 | 1.05 (0.91-1.22) | 0.356 | 9 | Age (continuous), education level, BMI, smoking status, alcohol drinking status, history of hypertension, history of diabetes, energy intake, fish intake, vegetable and fruit intake | (26) |
| Lu et al. 2022 (Female) | Japan | 1990 | Miyagi Cohort | 40–64 | Male & female (data shown for females) | FFQ | 3 times/week or almost daily  Almost never (ref.) | 17596 | 2522 | 25 | 0.89 (0.74-1.07) | 0.016 | 9 | Age (continuous), education level, BMI, smoking status, alcohol drinking status, history of hypertension, history of diabetes, energy intake, fish intake, vegetable and fruit intake | (26) |
| Ge et al. 2023 (Male) | Japan | 1995 | JPHC | 40–69 | Male & female (data shown for males) | FFQ | Q4: 6.1 g/day (energy-adj.)  Q1: 0 g/day (ref.)* | 43117 | 14211 | 19.3 | 0.99 (0.94-1.04) | 0.79 | 7 | Age, study area, smoking status, alcohol frequency, BMI, physical activity, hypertension with medication, self, reported diabetes, green tea, coffee, energy-adjusted consumption of vegetables and fruits, total energy and total fat, menopausal status (only for females), exogenous hormone use (only for females), dairy intake | (27) |
| Ge et al. 2023 (Female) | Japan | 1995 | JPHC | 40–69 | Male & female (data shown for females) | FFQ | Q4: 7.6 g/day (energy-adj.)  Q1: 0 g/day (ref.)* | 50193 | 9547 | 19.3 | 0.99 (0.92-1.05) | 0.68 | 7 | Age, study area, smoking status, alcohol frequency, BMI, physical activity, hypertension with medication, self, reported diabetes, green tea, coffee, energy-adjusted consumption of vegetables and fruits, total energy and total fat, menopausal status (only for females), exogenous hormone use (only for females), dairy intake | (27) |
| **Cheese / CVD** | | | | | | | | | | | | | | | |
| Mann et al. 1997 | UK | 1980 | UK Health-Conscious Diet Cohort | 16–79 | Male & female | SFFQ | C3: ≥ 5 times/week C1: < 1 times/week (ref.) | 10802 | 64 | 13.3 | 2.47 (0.97-6.26) | 0.01 | 6 | Age, sex, smoking, social class | (37) |
| Bonthuis et al. 2010 | Australia | 1992 | Nambour Skin Cancer Study | 25–78 | Male & female | FFQ | Q3: 30 g/day  Q1: 4 g/day (ref.)* | 1529 | 61 | 14.4 | 0.64 (0.27-1.49) | 0.54 | 8 | Age, sex, total energy intake, BMI, alcohol intake, school leaving age, physical activity level, pack-years of smoking, dietary supplement use, beta-carotene treatment during trial, presence of any medical condition, dietary calcium | (13) |
| Goldbohm et al. 2011 (Female Stroke M.) | Netherlands | 1986 | NLCS | 55–69 | Male & female (data shown for females) | FFQ | Q5/C5: 56 g/day  Q1/C1: 1 g/day (ref.)* | 62573 | 322 | 10 | 0.65 (0.39-1.1) | 0.169 | 8 | Age, education, smoking, physical activity, BMI, multivitamin use, alcohol, energy, energy-adjusted mono- and polyunsaturated fat intakes, vegetable and fruit consumption | (14) |
| Goldbohm et al. 2011 (Male Stroke M.) | Netherlands | 1986 | NLCS | 55–69 | Male & female (data shown for males) | FFQ | Q5/C5: 56 g/day  Q1/C1: 1 g/day (ref.)* | 58279 | 520 | 10 | 1.11 (0.75-1.64) | 0.403 | 8 | Age, education, smoking, physical activity, BMI, multivitamin use, alcohol, energy, energy-adjusted mono- and polyunsaturated fat intakes, vegetable and fruit consumption | (14) |
| Goldbohm et al. 2011 (Female IHD M.) | Netherlands | 1986 | NLCS | 55–69 | Male & female (data shown for females) | FFQ | Q5/C5: 56 g/day  Q1/C1: 1 g/day (ref.)* | 62573 | 692 | 10 | 1.01 (0.68-1.5) | 0.832 | 8 | Age, education, smoking, physical activity, BMI, multivitamin use, alcohol, energy, energy-adjusted mono- and polyunsaturated fat intakes, vegetable and fruit consumption | (14) |
| Goldbohm et al. 2011 (Male IHD M.) | Netherlands | 1986 | NLCS | 55–69 | Male & female (data shown for males) | FFQ | Q5/C5: 56 g/day  Q1/C1: 1 g/day (ref.)* | 58279 | 1997 | 10 | 0.97 (0.74-1.27) | 0.639 | 8 | Age, education, smoking, physical activity, BMI, multivitamin use, alcohol, energy, energy-adjusted mono- and polyunsaturated fat intakes, vegetable and fruit consumption | (14) |
| van Aerde et al. 2013 | Netherlands | 1989 | The Hoorn Study | 50–75 | Male & female | FFQ | Q4: ≥14.7 g/day Q1: ≤6.0 g/day (ref.)* | 1956 | 116 | 12.4 | 1.09 (0.87-1.35) | 0.46 | 8 | Age, sex, BMI, smoking, educational level, total energy intake, alcohol consumption, physical activity, intake of meat, fish, bread, vegetables, fruit, coffee, tea | (8) |
| Praagman et al. 2015 | Netherlands | 1993 | EPIC-NL | 20–70 | Male & female | FFQ | Q4: 53.2 g/day (energy-adj.)  Q1: 6.6 g/day (ref.)* | 34409 | 727 | 15 | 0.8 (0.65-0.99) | 0.1 | 8 | Age, sex, smoking habit, BMI, physical activity, education level, hypertension at baseline, intakes of alcohol and energy-adjusted intakes of fruit and vegetables, total energy intake | (6) |
| Praagman et al. 2015 (CHD M.) | Netherlands | 1990 | Rotterdam Study | ≥ 55 | Male & female | SFFQ | T3: >40 g/day  T1: <20 g/day (ref.)* | 4235 | 350 | 17.3 | 1.18 (0.86-1.64) | 0.36 | 8 | Age, sex, total energy intake, BMI, smoking, education level, alcohol intake, intakes of vegetables, fruit, meat, bread, fish coffee, tea | (11) |
| Praagman et al. 2015 (Stroke M.) | Netherlands | 1990 | Rotterdam Study | ≥ 55 | Male & female | SFFQ | T3: >40 g/day  T1: <20 g/day (ref.)* | 4235 | 182 | 17.3 | 1.07 (0.7-1.64) | 0.79 | 8 | Age, sex, total energy intake, BMI, smoking, education level, alcohol intake, intakes of vegetables, fruit, meat, bread, fish coffee, tea | (11) |
| Farvid et al. 2017 | Iran | 2004 | Golestan Study | 36–85 | Male & female | FFQ | Q5: 0.8 servings/day  Q1: 0 servings/day (ref.)** | 42403 | 1467 | 8 | 0.74 (0.61-0.91) | 0.02 | 8 | Age, ethnicity, education, marital status, residency, smoking, opium use, alcohol use, BMI, systolic blood pressure, occupational physical activity, family history of cancer, wealth score, medication use, energy intake | (18) |
| Ding et al. 2019 (NHS) | USA | 1976 | NHS, NHS II, HPFS | 30–55 | Male & female (data shown for females) | FFQ | C4: > 34.5 g/day C1: < 30 g/week (ref.) | 74805 | 4418 | 40 | 1.15 (0.95-1.39) | 0.23 | 8 | Age, family history of CVD, history of cancer, family history of cardiovascular disease, baseline disease status (hypertension, hypercholesterolemia), baseline BMI, physical activity, alternate healthy eating index score, total energy intake, smoking status, alcohol consumption, postmenopausal status (only for females), current postmenopausal hormone use (only for females) | (40) |
| Ding et al. 2019 (HPFS) | USA | 1986 | NHS, NHS II, HPFS | 40–75 | Male & female (data shown for males) | FFQ | C4: > 34.8 g/day C1: < 30 g/week (ref.) | 49602 | 7467 | 30 | 0.86 (0.75-0.99) | 0.05 | 8 | Age, family history of CVD, history of cancer, family history of cardiovascular disease, baseline disease status (hypertension, hypercholesterolemia), baseline BMI, physical activity, alternate healthy eating index score, total energy intake, smoking status, alcohol consumption, postmenopausal status (only for females), current postmenopausal hormone use (only for females) | (40) |
| Ding et al. 2019 (NHSII) | USA | 1989 | NHS, NHS II, HPFS | 25–42 | Male & female (data shown for females) | FFQ | C4: > 42 g/day C1: < 30 g/week (ref.) | 93348 | 258 | 27 | 1.4 (0.7-2.79) | 0.01 | 8 | Age, family history of CVD, history of cancer, family history of cardiovascular disease, baseline disease status (hypertension, hypercholesterolemia), baseline BMI, physical activity, alternate healthy eating index score, total energy intake, smoking status, alcohol consumption, postmenopausal status (only for females), current postmenopausal hormone use (only for females) | (40) |
| Pala et al. 2019 | Italy | 1993 | EPIC-Italy | 45–64 | Male & female | FFQ | Q4: >100 g/day  Q1: 0 to ≤28 g/day (ref.) | 45009 | 459 | 14.9 | 0.88 (0.6-1.3) | 0.61 | 8 | Region, sex, age, energy intake, weight, height, waist-to-hip ratio, alcohol consumption, smoking status, physical activity, relative index of inequality, Italian Mediterranean Index, intake of sugar | (20) |
| Mazidi et al. 2019 | USA | 1999 | NHANES | > 20 | Male & female | 24-h recall | Q4: 4.6 oz natural cheese or 6.2 oz processed cheese Q1: 0.38 oz natural cheese or 0.5 oz processed cheese* | 24474 | 709 | 6.4 | 1.04 (0.88-1.11) | 0.723 | 9 | Age, sex, race, education, marital status, poverty to income ratio, total energy intake, physical activity, smoking, alcohol consumption, carbohydrates, saturated fat, protein, dietary fiber, BMI, hypertension, diabetes | (21) |
| Lu et al. 2022 (Male) | Japan | 1990 | Miyagi Cohort | 40–64 | Male & female (data shown for males) | FFQ | 3 times/week or almost daily  Almost never (ref.) | 16565 | 1048 | 25 | 1.01 (0.75-1.34) | 0.136 | 9 | Age (continuous), education level, BMI, smoking status, alcohol drinking status, history of hypertension, history of diabetes, energy intake, fish intake, vegetable and fruit intake | (26) |
| Lu et al. 2022 (Female) | Japan | 1990 | Miyagi Cohort | 40–64 | Male & female (data shown for females) | FFQ | 3 times/week or almost daily  Almost never (ref.) | 17596 | 645 | 25 | 0.99 (0.7-1.41) | 0.634 | 9 | Age (continuous), education level, BMI, smoking status, alcohol drinking status, history of hypertension, history of diabetes, energy intake, fish intake, vegetable and fruit intake | (26) |
| Ge et al. 2023 (Male) | Japan | 1995 | JPHC | 40–69 | Male & female (data shown for males) | FFQ | Q4: 6.1 g/day (energy-adj.)  Q1: 0 g/day (ref.)* | 43117 | 3379 | 19.3 | 0.87 (0.78-0.97) | 0.04 | 7 | Age, study area, smoking status, alcohol frequency, BMI, physical activity, hypertension with medication, self, reported diabetes, green tea, coffee, energy-adjusted consumption of vegetables and fruits, total energy and total fat, menopausal status (only for females), exogenous hormone use (only for females), dairy intake | (27) |
| Ge et al. 2023 (Female) | Japan | 1995 | JPHC | 40–69 | Male & female (data shown for females) | FFQ | Q4: 7.6 g/day (energy-adj.)  Q1: 0 g/day (ref.)* | 50193 | 2582 | 19.3 | 0.91 (0.8-1.04) | 0.43 | 7 | Age, study area, smoking status, alcohol frequency, BMI, physical activity, hypertension with medication, self, reported diabetes, green tea, coffee, energy-adjusted consumption of vegetables and fruits, total energy and total fat, menopausal status (only for females), exogenous hormone use (only for females), dairy intake | (27) |
| **Cheese / Cancer** | | | | | | | | | | | | | | | |
| Khan et al. 2004 (Male) | Japan | 1984 | Hokkaido Study | 40–97 | Male & female (data shown for males) | FFQ | C5: several times per week, everyday  C1: never, several times per year, several times per month (ref.) | 1524 | 155 | 13.8 | 1 (0.7-1.7) |  | 7 | Age, health status, health education, health screening, smoking | (30) |
| Khan et al. 2004 (Female) | Japan | 1984 | Hokkaido Study | 40–97 | Male & female (data shown for females) | FFQ | C5: several times per week, everyday  C1: never, several times per year, several times per month (ref.) | 1634 | 89 | 14.8 | 1.1 (0.6-2.2) |  | 7 | Age, health status, health education, health screening, smoking | (30) |
| Bonthuis et al. 2010 | Australia | 1992 | Nambour Skin Cancer Study | 25–78 | Male & female | FFQ | Q3: 30 g/day  Q1: 4 g/day (ref.) | 1529 | 58 | 14.4 | No association between cheese intake and Cancer mortality (Data are not provided). (nan-nan) | | 8 | Age, sex, total energy intake, BMI, alcohol intake, school leaving age, physical activity level, pack-years of smoking, dietary supplement use, beta-carotene treatment during trial, presence of any medical condition, dietary calcium | (13) |
| Praagman et al. 2015 | Netherlands | 1993 | EPIC-NL | 20–70 | Male & female | FFQ | Q4: 53.2 g/day (energy-adj.)  Q1: 6.6 g/day (ref.)* | 34409 | 1216 | 15 | 1.11 (0.94-1.3) | 0.3 | 8 | Age, sex, smoking habit, BMI, physical activity, education level, hypertension at baseline, intakes of alcohol and energy-adjusted intakes of fruit and vegetables, total energy intake | (6) |
| Farvid et al. 2017 | Iran | 2004 | Golestan Study | 36–85 | Male & female | FFQ | Q5: 0.8 servings/day  Q1: 0 servings/day (ref.)** | 42403 | 859 | 8 | 0.98 (0.75-1.29) | 0.99 | 8 | Age, ethnicity, education, marital status, residency, smoking, opium use, alcohol use, BMI, systolic blood pressure, occupational physical activity, family history of cancer, wealth score, medication use, energy intake | (18) |
| Ding et al. 2019 (NHS) | USA | 1976 | NHS, NHS II, HPFS | 30–55 | Male & female (data shown for females) | FFQ | C4: > 33.9 g/day C1: < 30 g/week (ref.) | 74805 | 7641 | 40 | 1.13 (0.98-1.3) | 0.01 | 8 | Age, family history of CVD, history of cancer, family history of cardiovascular disease, baseline disease status (hypertension, hypercholesterolemia), baseline BMI, physical activity, alternate healthy eating index score, total energy intake, smoking status, alcohol consumption, postmenopausal status (only for females), current postmenopausal hormone use (only for females) | (40) |
| Ding et al. 2019 (NHSII) | USA | 1989 | NHS, NHS II, HPFS | 25–42 | Male & female (data shown for females) | FFQ | C4: > 32.7 g/day C1: < 30 g/week (ref.) | 93348 | 1157 | 27 | 1.09 (0.76-1.55) | 0.41 | 8 | Age, family history of CVD, history of cancer, family history of cardiovascular disease, baseline disease status (hypertension, hypercholesterolemia), baseline BMI, physical activity, alternate healthy eating index score, total energy intake, smoking status, alcohol consumption, postmenopausal status (only for females), current postmenopausal hormone use (only for females) | (40) |
| Ding et al. 2019 (HPFS) | USA | 1986 | NHS, NHS II, HPFS | 40–75 | Male & female (data shown for males) | FFQ | C4: > 27.3 g/day C1: < 30 g/week (ref.) | 49602 | 6322 | 30 | 0.91 (0.78-1.07) | 0.39 | 8 | Age, family history of CVD, history of cancer, family history of cardiovascular disease, baseline disease status (hypertension, hypercholesterolemia), baseline BMI, physical activity, alternate healthy eating index score, total energy intake, smoking status, alcohol consumption, postmenopausal status (only for females), current postmenopausal hormone use (only for females) | (40) |
| Pala et al. 2019 | Italy | 1993 | EPIC-Italy | 45–64 | Male & female | FFQ | Q4: >100 g/day  Q1 : 0 to ≤28 g/day (ref.) | 45009 | 1456 | 14.9 | 1.08 (0.88-1.32) | 0.77 | 8 | Region, sex, age, energy intake, weight, height, waist-to-hip ratio, alcohol consumption, smoking status, physical activity, relative index of inequality, Italian Mediterranean Index, intake of sugar | (20) |
| Mazidi et al. 2019 | USA | 1999 | NHANES | > 20 | Male & female | 24-h recall | Q4: 4.6 oz natural cheese or 6.2 oz processed cheese Q1: 0.38 oz natural cheese or 0.5 oz processed cheese* | 24474 | 827 | 6.4 | 0.99  (0.98-1.02) | 0.852 | 9 | Age, sex, race, education, marital status, poverty to income ratio, total energy intake, physical activity, smoking, alcohol consumption, carbohydrates, saturated fat, protein, dietary fiber, BMI, hypertension, diabetes | (21) |
| Lu et al. 2022 (Male) | Japan | 1990 | Miyagi Cohort | 40–64 | Male & female (data shown for males) | FFQ | 3 times/week or almost daily  Almost never (ref.) | 16565 | 1713 | 25 | 1.08 (0.85-1.36) | 0.14 | 9 | Age (continuous), education level, BMI, smoking status, alcohol drinking status, history of hypertension, history of diabetes, energy intake, fish intake, vegetable and fruit intake | (26) |
| Lu et al. 2022 (Female) | Japan | 1990 | Miyagi Cohort | 40–64 | Male & female (data shown for females) | FFQ | 3 times/week or almost daily  Almost never (ref.) | 17596 | 839 | 25 | 0.95 (0.7-1.31) | 0.923 | 9 | Age (continuous), education level, BMI, smoking status, alcohol drinking status, history of hypertension, history of diabetes, energy intake, fish intake, vegetable and fruit intake | (26) |
| Ge et al. 2023 (Male) | Japan | 1995 | JPHC | 40–69 | Male & female (data shown for males) | FFQ | Q4: 6.1 g/day (energy-adj.)  Q1: 0 g/day (ref.)* | 43117 | 5364 | 19.3 | 1.07 (0.98-1.16) | 0.11 | 7 | Age, study area, smoking status, alcohol frequency, BMI, physical activity, hypertension with medication, self, reported diabetes, green tea, coffee, energy-adjusted consumption of vegetables and fruits, total energy and total fat, menopausal status (only for females), exogenous hormone use (only for females), dairy intake | (27) |
| Ge et al. 2023 (Female) | Japan | 1995 | JPHC | 40–69 | Male & female (data shown for females) | FFQ | Q4: 7.6 g/day (energy-adj.)  Q1: 0 g/day (ref.)* | 50193 | 3076 | 19.3 | 1.1 (0.98-1.22) | 0.06 | 7 | Age, study area, smoking status, alcohol frequency, BMI, physical activity, hypertension with medication, self, reported diabetes, green tea, coffee, energy-adjusted consumption of vegetables and fruits, total energy and total fat, menopausal status (only for females), exogenous hormone use (only for females), dairy intake | (27) |
| **Cheese / GI cancer** | | | | | | | | | | | | | | | |
| Kojima et al. 2004 (Male Colon CA) | Japan | 1988 | JACC | 40–79 | Male & female (data shown for males) | FFQ | 1–7 times/week  Seldom (ref.) | 45181 | 138 | 9.9 | 1.17 (0.68-2.01) | 0.53 | 8 | Age, family history of colorectal cancer, BMI, frequency of alcohol intake, current smoking status, walking time per day, educational level | (32) |
| Kojima et al. 2004 (Female Colon CA) | Japan | 1988 | JACC | 40–79 | Male & female (data shown for females) | FFQ | 1–7 times/week  Seldom (ref.) | 62643 | 146 | 9.9 | 1.01 (0.61-1.69) | 0.98 | 8 | Age, family history of colorectal cancer, BMI, frequency of alcohol intake, current smoking status, walking time per day, educational level | (32) |
| Kojima et al. 2004 (Male Rectal CA) | Japan | 1988 | JACC | 40–79 | Male & female (data shown for males) | FFQ | 1–7 times/week  Seldom (ref.) | 45181 | 116 | 9.9 | 1.19 (0.7-2.02) | 0.38 | 8 | Age, family history of colorectal cancer, BMI, frequency of alcohol intake, current smoking status, walking time per day, educational level | (32) |
| Kojima et al. 2004 (Female Rectal CA) | Japan | 1988 | JACC | 40–79 | Male & female (data shown for females) | FFQ | 1–7 times/week  Seldom (ref.) | 62643 | 57 | 9.9 | 2.52 (1.11-5.72) | 0.07 | 8 | Age, family history of colorectal cancer, BMI, frequency of alcohol intake, current smoking status, walking time per day, educational level | (32) |
| Khan et al. 2004 (Male Stomach CA) | Japan | 1984 | Hokkaido Study | 40–97 | Male & female (data shown for males) | FFQ | C5: several times per week, everyday  C1: never, several times per year, several times per month (ref.) | 1524 | 36 | 13.8 | 1.2 (0.5-3.0) |  | 7 | Age, health status, health education, health screening, smoking | (30) |
| Khan et al. 2004 (Female Stomach CA) | Japan | 1984 | Hokkaido Study | 40–97 | Male & female (data shown for females) | FFQ | C5: several times per week, everyday  C1: never, several times per year, several times per month (ref.) | 1634 | 15 | 14.8 | 1.2 (0.3-5.4) |  | 7 | Age, health status, health education, health screening, smoking | (30) |
| Khan et al. 2004 (Male Pancreatic CA) | Japan | 1984 | Hokkaido Study | 40–97 | Male & female (data shown for males) | FFQ | C5: several times per week, everyday  C1: never, several times per year, several times per month (ref.) | 1524 | 12 | 13.8 | 0.6 (0.1-4.4) |  | 7 | Age, health status, health education, health screening, smoking | (30) |
| Khan et al. 2004 (Female Pancreatic CA) | Japan | 1984 | Hokkaido Study | 40–97 | Male & female (data shown for females) | FFQ | C5: several times per week, everyday  C1: never, several times per year, several times per month (ref.) | 1634 | 13 | 14.8 | 1.7 (0.4-7.9) |  | 7 | Age, health status, health education, health screening, smoking | (30) |
| Khan et al. 2004 (Female Colorectal CA) | Japan | 1984 | Hokkaido Study | 40–97 | Male & female (data shown for females) | FFQ | C5: several times per week, everyday  C1: never, several times per year, several times per month (ref.) | 1634 | 14 | 14.8 | 1.5 (0.3-6.8) |  | 7 | Age, health status, health education, health screening, smoking | (30) |
| Tokui et al. 2022 (Male Stomach CA) | Japan | 1988 | JACC | 40–79 | Male & female (data shown for males) | FFQ | >1 times/day  None (ref.) | 46465 | 574 | 9.9 | 0.79 (0.39-1.61) | 0.64 | 6 | Age | (33) |
| Tokui et al. 2022 (Female Stomach CA) | Japan | 1988 | JACC | 40–79 | Male & female (data shown for females) | FFQ | >1 times/day  None (ref.) | 64327 | 285 | 9.9 | 1.18 (0.52-2.69) | 0.8 | 6 | Age | (33) |
| **Cheese / Lung cancer** | | | | | | | | | | | | | | | |
| Ozasa et al. 2001 (Male) | Japan | 1988 | JACC | 40–79 | Male & female (data shown for males) | FFQ | Q3: >3 dishes/day Q1: <1 dishes/day (ref.) | 42940 | 446 | 7.7 | 0.59 (0.38-0.91) | 0.0029 | 8 | Age, parents' history of lung cancer, smoking status, smoking index, time since quitting smoking | (34) |
| Ozasa et al. 2001 (Female) | Japan | 1988 | JACC | 40–79 | Male & female (data shown for females) | FFQ | Q3: >3 dishes/day Q1: <1 dishes/day (ref.) | 55308 | 126 | 7.7 | 0.81 (0.39-1.66) | 0.33 | 8 | Age, parents' history of lung cancer, smoking status, smoking index, time since quitting smoking | (34) |
| Khan et al. 2004 (Male) | Japan | 1984 | Hokkaido Study | 40–97 | Male & female (data shown for males) | FFQ | C5: several times per week, everyday  C1: never, several times per year, several times per month (ref.) | 1524 | 41 | 13.8 | 1.6 (0.8-3.4) |  | 7 | Age, health status, health education, health screening, smoking | (30) |
| Khan et al. 2004 (Female) | Japan | 1984 | Hokkaido Study | 40–97 | Male & female (data shown for females) | FFQ | C5: several times per week, everyday  C1: never, several times per year, several times per month (ref.) | 1634 | 10 | 14.8 | 1.1 (0.1-8.8) |  | 7 | Age, health status, health education, health screening, smoking | (30) |
| **Cheese / Reproductive cancer** | | | | | | | | | | | | | | | |
| Mills et al. 1988 | USA | 1960 | AHS | 30–85 | Female | FFQ | C4: >3 days/week C1: none/occasional (ref.) | 16190 | 142 | 20 | 1.25 (0.6-2.61) | 0.98 | 8 | Age at menarche, age at first pregnancy, age at menopause, percent desirable weight, education, consumption of other animal products | (41) |
| Park et al. 2007 | USA | 1995 | NIH-AARP | 50–71 | Male | FFQ | C5: > 3 servings/day  C1: < 0.5 servings/day (ref.) | 293888 | 178 | 6 | 1.24 (0.56-2.75) | 0.74 | 7 | Age, race/ethnicity, education, marital status, BMI, physical activity, smoking, alcohol consumption, history of diabetes, family history of prostate cancer, PSA screening, dietary calcium, energy intake | (35) |
| Sakauchi et al. 2007 | Japan | 1988 | JACC | 40–79 | Female | FFQ | Q3: ≥1–2 times/week Q1: seldom (ref.) | 63541 | 77 | 13.3 | 1.66 (0.65-4.25) | 0.27 | 7 | Age, menopausal status, number of pregnancies, history of sex hormone use, BMI, physical activity, education | (36) |

*Median **Mean

CA, cancer; CHD, coronary heart disease; CI, confidence interval; CVD, cardiovascular disease; FF, full-fat; FFQ, food frequency questionnaire; FU, follow-up; GI, gastrointestinal; HR, hazard ratio; IHD, ischemic heart disease; LF, low-fat; M., mortality; NOS, Newcastle-Ottawa Scale; NR, not reported; Ref., reference; SFFQ, semi-quantitative food frequency questionnaire.

# Supplementary Table 4. Association of fermented soy products with all-cause mortality and cause-specific mortality.

| **Study & subgroup details** | **Region** | **Start year** | **Cohort** | **Age range at entry** | **Sex** | **Dietary assessm.** | **Exposure levels** | **No. of subjects** | **No. of deaths** | **Years of FU** | **HR (95% CI)** | **p-value** | **NOS** | **Adjustments** | **Ref.** |
| --- | --- | --- | --- | --- | --- | --- | --- | --- | --- | --- | --- | --- | --- | --- | --- |
| **Fermented soy** | | | | | | | | | | | | | | | |
| **Fermented soy / All-causes** | | | | | | | | | | | | | | | |
| Katagiri et al. 2019 | Japan | 1990 | JPHC | 45–74 | Male & female (data shown for males) | FFQ | Q5: >50.2 g/day (energy-adj.) Q1: <13.4 g/day (ref.) | 42750 | 8370 | 14.8 | 0.9 (0.83-0.97) | 0.05 | 8 | Age, geographical area, smoking, frequency of alcohol intake, BMI, sports or physical exercise, history of diabetes or taking drugs for diabetes, taking antihypertensives, health check-up, total energy intake, intake of green tea, coffee, fish, meat, fruit, vegetables | (42) |
| Katagiri et al. 2019 | Japan | 1990 | JPHC | 45–74 | Male & female (data shown for females) | FFQ | Q5: >46.6 g/day (energy-adj.) Q1: <12.5 g/day (ref.) | 50165 | 4933 | 14.8 | 0.89 (0.8-0.98) | 0.01 | 8 | Age, geographical area, smoking, frequency of alcohol intake, BMI, sports or physical exercise, history of diabetes or taking drugs for diabetes, taking antihypertensives, health check-up, total energy intake, intake of green tea, coffee, fish, meat, fruit, vegetables | (42) |
| **Fermented soy / CVD** | | | | | | | | | | | | | | | |
| Katagiri et al. 2019 | Japan | 1990 | JPHC | 45–74 | Male & female (data shown for males) | FFQ | Q5: >50.2 g/day (energy-adj.) Q1: <13.4 g/day (ref.) | 42750 | 2000 | 14.8 | 0.82 (0.7-0.97) | 0.04 | 8 | Age, geographical area, smoking, frequency of alcohol intake, BMI, sports or physical exercise, history of diabetes or taking drugs for diabetes, taking antihypertensives, health check-up, total energy intake, intake of green tea, coffee, fish, meat, fruit, vegetables | (42) |
| Katagiri et al. 2019 | Japan | 1990 | JPHC | 45–74 | Male & female (data shown for females) | FFQ | Q5: >46.6 g/day (energy-adj.) Q1: <12.5 g/day (ref.) | 50165 | 1326 | 14.8 | 0.89 (0.73-1.07) | 0.25 | 8 | Age, geographical area, smoking, frequency of alcohol intake, BMI, sports or physical exercise, history of diabetes or taking drugs for diabetes, taking antihypertensives, health check-up, total energy intake, intake of green tea, coffee, fish, meat, fruit, vegetables | (42) |
| **Fermented soy / Cancer** | | | | | | | | | | | | | | | |
| Katagiri et al. 2019 | Japan | 1990 | JPHC | 45–74 | Male & female (data shown for males) | FFQ | Q5: >50.2 g/day (energy-adj.) Q1: <13.4 g/day (ref.) | 42750 | 3320 | 14.8 | 0.97 (0.85-1.09) | 0.66 | 8 | Age, geographical area, smoking, frequency of alcohol intake, BMI, sports or physical exercise, history of diabetes or taking drugs for diabetes, taking antihypertensives, health check-up, total energy intake, intake of green tea, coffee, fish, meat, fruit, vegetables | (42) |
| Katagiri et al. 2019 | Japan | 1990 | JPHC | 45–74 | Male & female (data shown for females) | FFQ | Q5: >46.6 g/day (energy-adj.) Q1: <12.5 g/day (ref.) | 50165 | 1817 | 14.8 | 0.94 (0.79-1.11) | 0.3 | 8 | Age, geographical area, smoking, frequency of alcohol intake, BMI, sports or physical exercise, history of diabetes or taking drugs for diabetes, taking antihypertensives, health check-up, total energy intake, intake of green tea, coffee, fish, meat, fruit, vegetables | (42) |

| **Study & subgroup details** | **Region** | **Start year** | **Cohort** | **Age range at entry** | **Sex** | **Dietary assessm.** | **Exposure levels** | **No. of subjects** | **No. of deaths** | **Years of FU** | **HR (95% CI)** | **p-value** | **NOS** | **Adjustments** | **Ref.** |
| --- | --- | --- | --- | --- | --- | --- | --- | --- | --- | --- | --- | --- | --- | --- | --- |
| **Miso** | | | | | | | | | | | | | | | |
| **Miso / All-causes** | | | | | | | | | | | | | | | |
| Katagiri et al. 2020 (Male) | Japan | 1990 | JPHC | 45–74 | Male & female (data shown for males) | FFQ | Q5: >31.1 g/day (energy-adj.) Q1: <7.7 g/day (ref.) | 42750 | 8370 | 14.8 | 0.95 (0.87-1.02) | 0.2 | 8 | Age, geographical area, smoking, frequency of alcohol intake, BMI, sports or physical exercise, history of diabetes or taking drugs for diabetes, taking antihypertensives, health check-up, total energy intake, intake of green tea, coffee, fish, meat, fruit, vegetables | (42) |
| Katagiri et al. 2020 (Female) | Japan | 1990 | JPHC | 45–74 | Male & female (data shown for females) | FFQ | Q5: >26.3 g/day (energy-adj.) Q1: <6.4 g/day (ref.) | 50165 | 4933 | 14.8 | 0.89 (0.81-0.97) | 0.03 | 8 | Age, geographical area, smoking, frequency of alcohol intake, BMI, sports or physical exercise, history of diabetes or taking drugs for diabetes, taking antihypertensives, health check-up, total energy intake, intake of green tea, coffee, fish, meat, fruit, vegetables | (42) |
| **Miso / CVD** | | | | | | | | | | | | | | | |
| Nguyen et al. 2018 | Japan | 1980 | NIPPON DATA80 | ≥ 30 | Male & female | 3-day weighed food record | Q4 : 11.0 ± 7.5 g/1000kcal (energy-adj.)  Q1 : 8.8 ± 7.0 g/1000kcal (ref.)** | 9244 | 417 | 24 | No significant association (Data are not provided). (nan-nan) | | 9 | Age, smoking status, drinking status, BMI, residential area, sodium intake, vegetable intake, fruit intake, fish intake, meat intake, milk and dairy products intake | (43) |
| Katagiri et al. 2020 (Male) | Japan | 1990 | JPHC | 45–74 | Male & female (data shown for males) | FFQ | Q5: >31.1 g/day (energy-adj.) Q1: <7.7 g/day (ref.) | 42750 | 2000 | 14.8 | 0.95 (0.82-1.1) | 0.84 | 8 | Age, geographical area, smoking, frequency of alcohol intake, BMI, sports or physical exercise, history of diabetes or taking drugs for diabetes, taking antihypertensives, health check-up, total energy intake, intake of green tea, coffee, fish, meat, fruit, vegetables | (42) |
| Katagiri et al. 2020 (Female) | Japan | 1990 | JPHC | 45–74 | Male & female (data shown for females) | FFQ | Q5: >26.3 g/day (energy-adj.) Q1: <6.4 g/day (ref.) | 50165 | 1326 | 14.8 | 0.94 (0.79-1.13) | 0.86 | 8 | Age, geographical area, smoking, frequency of alcohol intake, BMI, sports or physical exercise, history of diabetes or taking drugs for diabetes, taking antihypertensives, health check-up, total energy intake, intake of green tea, coffee, fish, meat, fruit, vegetables | (42) |
| **Miso / Cancer** | | | | | | | | | | | | | | | |
| Khan et al. 2004 | Japan | 1984 | Hokkaido Study | 40–97 | Male & female (data shown for males) | FFQ | C5: several times per week, everyday  C1: never, several times per year, several times per month (ref.) | 1524 | 155 | 13.8 | 0.4 (0.1-1.1) | | 7 | Age, health status, health education, health screening, smoking | (30) |
| Katagiri et al. 2020 (Male) | Japan | 1990 | JPHC | 45–74 | Male & female (data shown for males) | FFQ | Q5: >31.1 g/day (energy-adj.) Q1: <7.7 g/day (ref.) | 42750 | 3320 | 14.8 | 1.02 (0.91-1.16) | 0.74 | 8 | Age, geographical area, smoking, frequency of alcohol intake, BMI, sports or physical exercise, history of diabetes or taking drugs for diabetes, taking antihypertensives, health check-up, total energy intake, intake of green tea, coffee, fish, meat, fruit, vegetables | (42) |
| Katagiri et al. 2020 (Female) | Japan | 1990 | JPHC | 45–74 | Male & female (data shown for females) | FFQ | Q5: >26.3 g/day (energy-adj.) Q1: <6.4 g/day (ref.) | 50165 | 1817 | 14.8 | 0.88 (0.75-1.03) | 0.23 | 8 | Age, geographical area, smoking, frequency of alcohol intake, BMI, sports or physical exercise, history of diabetes or taking drugs for diabetes, taking antihypertensives, health check-up, total energy intake, intake of green tea, coffee, fish, meat, fruit, vegetables | (42) |
| **Miso / GI cancer** | | | | | | | | | | | | | | | |
| Hirayama et al. 1981 (Male Gastric CA) | Japan | 1966 | Hirayama Cohort | ≥ 49 | Male & female (data shown for males) | Interview questionnaire | Daily intake  No intake (ref.) | 122261 | 2562 | 13 | 0.67 (0.56-0.8) | | 6 | Sex, age, smoking, occupation, residence, marital status, alcohol, rice, meat, fish, milk, pickles, green-yellow vegetables, hot green tea intake | (44) |
| Hirayama et al. 1981 (Female Gastric CA) | Japan | 1966 | Hirayama Cohort | ≥ 49 | Male & female (data shown for females) | Interview questionnaire | Daily intake  No intake (ref.) | 142857 | 1351 | 13 | 0.69 (0.54-0.87) | | 6 | Sex, age, smoking, occupation, residence, marital status, alcohol, rice, meat, fish, milk, pickles, green-yellow vegetables, hot green tea intake | (44) |
| Ngoan et al. 2002 (Male Stomach CA) | Japan | 1986 | Fukuoka Prefecture cohort | 15–96 | Male & female (data shown for males) | FFQ | C3: ≥ 2 times/day C1: ≤2-4 times/week (ref.) | 5917 | 77 | 13 | 1.4 (0.7-3.2) | | 5 | Age, sex | (45) |
| Ngoan et al. 2002 (Female Stomach CA) | Japan | 1986 | Fukuoka Prefecture cohort | 20–92 | Male & female (data shown for females) | FFQ | C3: ≥ 2 times/day C1: ≤2-4 times/week (ref.) | 7333 | 39 | 13 | 0.7 (0.2-3.4) | | 5 | Age, sex | (45) |
| Kurozawa et al. 2004 (Male Hepato CA) | Japan | 1988 | JACC | 40–59 | Male & female (data shown for males) | FFQ | Q3: 2- servings/day Q1: ≤ servings/day (ref.) | 46465 | 287 | 9.9 | 4.36 (0.99-19.33) | | 5 | No adjustment | (46) |
| Kurozawa et al. 2004 (old Men Hepato CA) | Japan | 1988 | JACC | 60–79 | Male & female (data shown for males) | FFQ | Q3: 2- servings/day Q1: ≤ servings/day (ref.) | 46465 | 287 | 9.9 | 1.12 (0.43-2.91) | | 5 | No adjustment | (46) |
| Kurozawa et al. 2004 (Female Hepato CA) | Japan | 1988 | JACC | 40–59 | Male & female (data shown for females) | FFQ | Q3: 2- servings/day Q1: ≤ servings/day (ref.) | 64327 | 114 | 9.9 | 0.31 (0.03-3.12) | | 5 | No adjustment | (46) |
| Kurozawa et al. 2004 (old Female Hepato CA) | Japan | 1988 | JACC | 60–79 | Male & female (data shown for females) | FFQ | Q3: 2- servings/day Q1: ≤ servings/day (ref.) | 64327 | 114 | 9.9 | 0.17 (0.04-0.67) | 0 .05 | 5 | No adjustment | (46) |
| Khan et al. 2004 (Stomach CA) | Japan | 1984 | Hokkaido Study | 40–97 | Male & female (data shown for males) | FFQ | C5: several times per week, everyday  C1: never, several times per year, several times per month (ref.) | 1524 | 36 | 13.8 | 0.2 (0.1-0.8) | 0.05 | 7 | Age, health status, health education, health screening, smoking | (30) |
| Tokui et al. 2022 (Male Stomach CA) | Japan | 1988 | JACC | 40–79 | Male & female (data shown for males) | FFQ | >1 times/day  None (ref.) | 46465 | 574 | 9.9 | 1.44 (0.86-2.42) | 0.36 | 6 | Age | (33) |
| Tokui et al. 2022 (Female Stomach CA) | Japan | 1988 | JACC | 40–79 | Male & female (data shown for females) | FFQ | >1 times/day  None (ref.) | 64327 | 285 | 9.9 | 1.46 (0.81-2.61) | 0.19 | 6 | Age | (33) |

| **Study & subgroup details** | **Region** | **Start year** | **Cohort** | **Age range at entry** | **Sex** | **Dietary assessm.** | **Exposure levels** | **No. of subjects** | **No. of deaths** | **Years of FU** | **HR (95% CI)** | **p-value** | **NOS** | **Adjustments** | **Ref.** |
| --- | --- | --- | --- | --- | --- | --- | --- | --- | --- | --- | --- | --- | --- | --- | --- |
| **Natto** | | | | | | | | | | | | | | | |
| **Natto / All-causes** | | | | | | | | | | | | | | | |
| Katagiri et al. 2019 | Japan | 1990 | JPHC | 45–74 | Male & female (data shown for males) | FFQ | Q5: >26.2 g/day (energy-adj.) Q1: 0 g/day (ref.) | 42750 | 8370 | 14.8 | 0.94 (0.87-1.02) | 0.1 | 8 | Age, geographical area, smoking, frequency of alcohol intake, BMI, sports or physical exercise, history of diabetes or taking drugs for diabetes, taking antihypertensives, health check-up, postmenopausal status (only for females), use of exogenous female hormones (only for females), total energy intake, intake of green tea, coffee, fish, meat, fruit, vegetables | (42) |
| Katagiri et al. 2019 | Japan | 1990 | JPHC | 45–74 | Male & female (data shown for females) | FFQ | Q5: >26.2 g/day (energy-adj.) Q1: 0 g/day (ref.) | 50165 | 4933 | 14.8 | 0.84 (0.76-0.93) | 0.001 | 8 | Age, geographical area, smoking, frequency of alcohol intake, BMI, sports or physical exercise, history of diabetes or taking drugs for diabetes, taking antihypertensives, health check-up, postmenopausal status (only for females), use of exogenous female hormones (only for females), total energy intake, intake of green tea, coffee, fish, meat, fruit, vegetables | (42) |
| **Natto / CVD** | | | | | | | | | | | | | | | |
| Nagata et al. 2017 | Japan | 1992 | Takayama Study | ≥ 35 | Male & female | FFQ | Q4: 7.3 g/day (energy-adj.)  Q1: 0 g/day (ref.)* | 29079 | 1678 | 16 | 0.75 (0.64-0.88) | 0.0004 | 8 | Age, sex, total energy, BMI, physical activity, smoking status, education, marital status, history of diabetes and hypertension, intakes of saturated fat, polyunsaturated fat, salt, vegetables, fruit | (47) |
| Katagiri et al. 2019 | Japan | 1990 | JPHC | 45–74 | Male & female (data shown for males) | FFQ | Q5: >26.2 g/day (energy-adj.) Q1: 0 g/day (ref.) | 42750 | 2000 | 14.8 | 0.76 (0.65-0.9) | 0.002 | 8 | Age, geographical area, smoking, frequency of alcohol intake, BMI, sports or physical exercise, history of diabetes or taking drugs for diabetes, taking antihypertensives, health check-up, total energy intake, intake of green tea, coffee, fish, meat, fruit, vegetables | (42) |
| Katagiri et al. 2019 | Japan | 1990 | JPHC | 45–74 | Male & female (data shown for females) | FFQ | Q5: >26.2 g/day (energy-adj.) Q1: 0 g/day (ref.) | 50165 | 1326 | 14.8 | 0.79 (0.65-0.95) | 0.01 | 8 | Age, geographical area, smoking, frequency of alcohol intake, BMI, sports or physical exercise, history of diabetes or taking drugs for diabetes, taking antihypertensives, health check-up, total energy intake, intake of green tea, coffee, fish, meat, fruit, vegetables | (42) |
| **Natto / Cancer** | | | | | | | | | | | | | | | |
| Katagiri et al. 2019 | Japan | 1990 | JPHC | 45–74 | Male & female (data shown for males) | FFQ | Q5: >26.2 g/day (energy-adj.) Q1: 0 g/day (ref.) | 42750 | 3320 | 14.8 | 0.98 (0.87-1.11) | 0.74 | 8 | Age, geographical area, smoking, frequency of alcohol intake, BMI, sports or physical exercise, history of diabetes or taking drugs for diabetes, taking antihypertensives, health check-up, total energy intake, intake of green tea, coffee, fish, meat, fruit, vegetables | (42) |
| Katagiri et al. 2019 | Japan | 1990 | JPHC | 45–74 | Male & female (data shown for females) | FFQ | Q5: >26.2 g/day (energy-adj.) Q1: 0 g/day (ref.) | 50165 | 1817 | 14.8 | 0.88 (0.75-1.04) | 0.28 | 8 | Age, geographical area, smoking, frequency of alcohol intake, BMI, sports or physical exercise, history of diabetes or taking drugs for diabetes, taking antihypertensives, health check-up, total energy intake, intake of green tea, coffee, fish, meat, fruit, vegetables | (42) |

*Median **Mean

CA, cancer; CI, confidence interval; CVD, cardiovascular disease; FFQ, food frequency questionnaire; FU, follow-up; GI, gastrointestinal; HR, hazard ratio; NOS, Newcastle-Ottawa Scale; Ref., reference; SFFQ, semi-quantitative food frequency questionnaire.

# Supplementary Table 5. Association of bread and chocolate/cocoa products with all-cause mortality and cause-specific mortality.

| **Study & subgroup details** | **Region** | **Start year** | **Cohort** | **Age range at entry** | **Sex** | **Dietary assessm.** | **Exposure levels** | **No. of subjects** | **No. of deaths** | **Years of FU** | **HR (95% CI)** | **p-value** | **NOS** | **Adjustments** | **Ref.** |
| --- | --- | --- | --- | --- | --- | --- | --- | --- | --- | --- | --- | --- | --- | --- | --- |
| **Bread** | | | | | | | | | | | | | | | |
| **Bread / All-causes** | | | | | | | | | | | | | | | |
| Fortes et al. 2000 | Italy | 1993 | Rome Elderly Cohort | ≥ 65 | Male & female | FFQ | T3: > 3 times/week  T1: <1 times/week (ref.) | 161 | 53 | 5 | 0.65 (0.29-1.45) |  | 7 | No adjustments | (38) |
| Bongard et al. 2016 | France | 1995 | MONICA | 45–64 | Male | 3-day food record | Q4: 460 g/day (energy-adj.)  Q1: 0 g/day (ref.)* | 960 | 150 | 14.8 | 0.8 (0.51-1.26) | 0.08 | 8 | Center, age, payment of income tax, obesity, alcohol consumption, smoking habits, physical activity, presence of a serious chronic condition, diet quality score | (16) |
| **Bread / CVD** | | | | | | | | | | | | | | | |
| Rebello et al. 2014 (Male Whole W.) | Singapore | 1993 | SCHS | 45–74 | Male & female (data shown for males) | SFFQ | Q3: >1 slices/day  Q1: 0 slices/day (ref.)* | 23501 | 1022 | 15 | 0.94 (0.66-1.33) | 0.095 | 9 | Age, dialect group, year of interview, energy intake, cigarette smoking, alcohol intake, physical activity, education, BMI, history of hypertension, use of hormone-replacement therapy (only for females), ratio of PUFAs to SFAs, cholesterol intake, fiber intake | (48) |
| Rebello et al. 2014 (Female Whole W.) | Singapore | 1993 | SCHS | 45–74 | Male & female (data shown for females) | SFFQ | Q3: >1 slices/day  Q1: 0 slices/day (ref.)* | 29968 | 638 | 15 | 0.51 (0.3-0.89) | 0.01 | 9 | Age, dialect group, year of interview, energy intake, cigarette smoking, alcohol intake, physical activity, education, BMI, history of hypertension, use of hormone-replacement therapy (only for females), ratio of PUFAs to SFAs, cholesterol intake, fiber intake | (48) |
| Rebello et al. 2014 (Male White W.) | Singapore | 1993 | SCHS | 45–74 | Male & female (data shown for males) | SFFQ | Q3: >1 slices/day  Q1: 0 slices/day (ref.)* | 23501 | 1022 | 15 | 1.12 (0.9-1.39) | 0.2 | 9 | Age, dialect group, year of interview, energy intake, cigarette smoking, alcohol intake, physical activity, education, BMI, history of hypertension, use of hormone-replacement therapy (only for females), ratio of PUFAs to SFAs, cholesterol intake, fiber intake | (48) |
| Rebello et al. 2014 (Female White W.) | Singapore | 1993 | SCHS | 45–74 | Male & female (data shown for females) | SFFQ | Q3: >1 slices/day  Q1: 0 slices/day (ref.)* | 29968 | 638 | 15 | 0.79 (0.6-1.04) | 0.11 | 9 | Age, dialect group, year of interview, energy intake, cigarette smoking, alcohol intake, physical activity, education, BMI, history of hypertension, use of hormone-replacement therapy (only for females), ratio of PUFAs to SFAs, cholesterol intake, fiber intake | (48) |
| Wada et al. 2022 (Male) | Japan | 1993 | Takayama Study | > 35 | Male & female (data shown for males) | FFQ | Q4: 76.7 g/day Q1: 2.9 g/day (ref.)* | 13355 | 779 | 14.1 | 0.92 (0.74-1.15) |  | 8 | Smoking status, physical activity, alcohol intake, coffee intake, salt Intake, marital status, education level, BMI, history of diabetes and hypertension, menopausal status (only for females) | (49) |
| Wada et al. 2022 (Female) | Japan | 1993 | Takayama Study | > 35 | Male & female (data shown for females) | FFQ | Q4: 78.8 g/day Q1: 7.2 g/day (ref.)* | 15724 | 907 | 14.1 | 0.97 (0.8-1.19) |  | 8 | Smoking status, physical activity, alcohol intake, coffee intake, salt Intake, marital status, education level, BMI, history of diabetes and hypertension, menopausal status (only for females) | (49) |
| **Bread / Lung cancer** | | | | | | | | | | | | | | | |
| Chow et al. 1992 | USA | 1966 | Lutheran Brotherhood Cohort | ≥ 35 | Male | FFQ | Q4: > 240 times/month Q1: < 91 times/month (ref.) | 17633 | 219 | 20 | 1.0 (0.6-1.7) |  | 8 | Age, smoking status, industry/occupation | (50) |
| Khan et al. 2004 (Male) | Japan | 1984 | Hokkaido Study | 40–97 | Male & female (data shown for males) | FFQ | C5: several times per week, everyday  C1: never, several times per year, several times per month (ref.) | 1524 | 41 | 13.8 | 0.7 (0.3-1.6) |  | 7 | Age, health status, health education, health screening, smoking | (30) |
| Khan et al. 2004 (Female) | Japan | 1984 | Hokkaido Study | 40–97 | Male & female (data shown for females) | FFQ | C5: several times per week, everyday  C1: never, several times per year, several times per month (ref.) | 1634 | 10 | 14.8 | 0.3 (0.0-2.5) |  | 7 | Age, health status, health education, health screening, smoking | (30) |
| **Choco&cocoa** | | | | | | | | | | | | | | | |
| **Choco&cocoa / All-causes** | | | | | | | | | | | | | | | |
| Paganini-Hill et al. 2007 | USA | 1980 | Leisure World Cohort | 44–101 | Male & female | FFQ | C5: few days/week to daily C1: rarely or never (ref.) | 13624 | 11386 | 23 | 0.98 (0.93-1.04) |  | 7 | Age, sex, smoking, exercise, BMI, alcohol intake, histories of hypertension, angina, heart attack, stroke, diabetes, rheumatoid arthritis, cancer | (51) |
| Zhong et al. 2021 | USA | 1993 | PLCO | 55–74 | Male & female | FFQ | Q4: >2 servings/week (energy-adj.)  Q1: 0 servings/week (ref.)** | 91891 | 19586 | 13.5 | 0.87 (0.82-0.93) | 0.009 | 8 | Age, sex, ethnicity, educational level, marital status, study center, history of hypertension, history of diabetes, aspirin use, hormone use status (only for females), smoking status, alcohol consumption, BMI, physical activity, energy intake from diet, consumption of red meat, processed meat, fruit, vegetable, whole grain, dairy, coffee, tea | (52) |
| Zhao et al. 2022 | Finland | 1986 | ATBC | 50–69 | Male | FFQ | Q5: 12.4 g/day (energy-adj.)  Q1: 0 g/day (ref.) | 27111 | 22064 | 31 | 0.88 (0.85-0.92) | 0.0001 | 8 | Age, BMI, energy intake, smoking, serum HDL and total cholesterol, intervention assignment, education, physical activity, alcohol consumption, Alternate MD score, systolic and diastolic blood pressure, history of cardiovascular disease and diabetes | (53) |
| Sun et al. 2023 | USA | 1993 | WHI | 50–79 | Female | FFQ | Q5: ≥1 servings/day  Q1: 0 servings/week (ref.) | 84709 | 25388 | 19 | 0.9 (0.84-0.97) | 0.02 | 8 | Αge, race, ethnicity, education, annual family income, neighborhood-level socioeconomic status, observational study/clinical trial, unopposed estrogen use, estrogen and progesterone use, smoking status, physical activity, ΒΜΙ, alcohol intake, coffee or tea intake, total energy intake, baseline diabetes status, baseline high blood cholesterol status, family history of heart attack or stroke, Healthy Eating Index-2015 score | (54) |
| **Choco&cocoa / CVD** | | | | | | | | | | | | | | | |
| Buijsse et al. 2006 | Netherlands | 1985 | Zutphen Elderly Study | 65–84 | Male | Interview questionnaire | Q3: 4.18 g/day Q1: 0.0 g/day (ref.)* | 470 | 152 | 15 | 0.5 (0.32-0.78) | 0.004 | 9 | Age, BMI, alcohol intake, physical activity, smoking, diet prescription, aspirin use, anticoagulant use, physician in blood pressure, diet prescription, dietary cholesterol, intake of trans fatty acids, saturated fat, folic acid, vit C, vit E, beta-carotene, K, sodium, Ca and Mg, total calories | (55) |
| Kwok et al. 2015 | UK | 1993 | EPIC-Norfolk | 40–79 | Male & female | FFQ | Q5: 15.6–98.8 g/day  Q1: 0 g/day (ref.)* | 20951 | 1107 | 11.3 | 0.75 (0.62-0.92) | 0.011 | 8 | Sex, age, smoking, physical activity, ΒΜΙ, energy intake, alcohol consumption, diabetes, systolic blood pressure, LDL cholesterol, HDL cholesterol | (56) |
| Ho et al. 2021 | USA | 2018 | Million Veteran Program | 64 ± 12 | Male & female | FFQ | Q5: ≥5 times/week (141.7 g) Q1: <1 times/month (28.3 g) (ref.) | 188447 | 6946 | 3.2 | 0.89 (0.84-0.96) | 0.0001 | 5 | Age, sex, race, BMI, smoking, physical activity, alcohol consumption | (57) |
| Zhong et al. 2021 | USA | 1993 | PLCO | 55–74 | Male & female | FFQ | Q4: >2 servings/week (energy-adj.)  Q1: 0 servings/week (ref.)** | 91891 | 5490 | 13.5 | 0.78 (0.7-0.88) | 0.02 | 8 | Age, sex, ethnicity, educational level, marital status, study center, history of hypertension, history of diabetes, aspirin use, hormone use status (only for females), smoking status, alcohol consumption, BMI, physical activity, energy intake from diet, consumption of red meat, processed meat, fruit, vegetable, whole grain, dairy, coffee, tea | (52) |
| Zhao et al. 2022 | Finland | 1986 | ATBC | 50–69 | Male | FFQ | Q5: 12.4 g/day (energy-adj.)  Q1: 0 g/day (ref.) | 27111 | 9121 | 31 | 0.87 (0.82-0.94) | 0.0002 | 8 | Age, BMI, energy intake, smoking, serum HDL and total cholesterol, intervention assignment, education, physical activity, alcohol consumption, Alternate MD score, systolic and diastolic blood pressure, history of cardiovascular disease and diabetes | (53) |
| Sun et al. 2023 | USA | 1993 | WHI | 50–79 | Female | FFQ | Q5: ≥1 servings/day  Q1: 0 servings/week (ref.) | 84709 | 7069 | 19 | 0.92 (0.8-1.05) |  | 8 | Αge, race, ethnicity, education, annual family income, neighborhood-level socioeconomic status, observational study/clinical trial, unopposed estrogen use, estrogen and progesterone use, smoking status, physical activity, ΒΜΙ, alcohol intake, coffee or tea intake, total energy intake, baseline diabetes status, baseline high blood cholesterol status, family history of heart attack or stroke, Healthy Eating Index-2015 score | (54) |

*Median **Mean

CA, cancer; CI, confidence interval; CVD, cardiovascular disease; FFQ, food frequency questionnaire; FU, follow-up; HR, hazard ratio; NOS, Newcastle-Ottawa Scale; Ref., reference; SFFQ, semi-quantitative food frequency questionnaire; W., wheat.

# References

1. Campbell M, McKenzie JE, Sowden A, Katikireddi SV, Brennan SE, Ellis S, et al. Synthesis without meta-analysis (SWiM) in systematic reviews: reporting guideline. BMJ. 2020 Jan 16;l6890.

2. Guyatt GH, Oxman AD, Kunz R, Brozek J, Alonso-Coello P, Rind D, et al. GRADE guidelines 6. Rating the quality of evidence—imprecision. Journal of Clinical Epidemiology. 2011 Dec;64(12):1283–93.

3. Guyatt GH, Oxman AD, Sultan S, Glasziou P, Akl EA, Alonso-Coello P, et al. GRADE guidelines: 9. Rating up the quality of evidence. Journal of Clinical Epidemiology. 2011 Dec;64(12):1311–6.

4. Murad MH, Mustafa RA, Schünemann HJ, Sultan S, Santesso N. Rating the certainty in evidence in the absence of a single estimate of effect. Evid Based Med. 2017 June;22(3):85–7.

5. Belbasis L, Bellou V, Evangelou E, Ioannidis JPA, Tzoulaki I. Environmental risk factors and multiple sclerosis: an umbrella review of systematic reviews and meta-analyses. The Lancet Neurology. 2015 Mar;14(3):263–73.

6. Praagman J, Dalmeijer GW, Van Der Schouw YT, Soedamah-Muthu SS, Monique Verschuren WM, Bas Bueno-de-Mesquita H, et al. The relationship between fermented food intake and mortality risk in the European Prospective Investigation into Cancer and Nutrition-Netherlands cohort. Br J Nutr. 2015 Feb 14;113(3):498–506.

7. Soedamah-Muthu SS, Masset G, Verberne L, Geleijnse JM, Brunner EJ. Consumption of dairy products and associations with incident diabetes, CHD and mortality in the Whitehall II study. Br J Nutr. 2013 Feb 28;109(4):718–26.

8. Van Aerde MA, Soedamah-Muthu SS, Geleijnse JM, Snijder MB, Nijpels G, Stehouwer CDA, et al. Dairy intake in relation to cardiovascular disease mortality and all-cause mortality: the Hoorn study. Eur J Nutr. 2013 Mar;52(2):609–16.

9. Virtanen HE, Voutilainen S, Koskinen TT, Mursu J, Kokko P, Ylilauri MP, et al. Dietary proteins and protein sources and risk of death: the Kuopio Ischaemic Heart Disease Risk Factor Study. Am J Clin Nutr. 2019 May;109(5):1462–71.

10. Guo J, Givens DI, Heitmann BL. Association between dairy consumption and cardiovascular disease events, bone fracture and all-cause mortality. Huang HK, editor. PLoS ONE. 2022 Sept 9;17(9):e0271168.

11. Praagman J, Franco OH, Ikram MA, Soedamah-Muthu SS, Engberink MF, Van Rooij FJA, et al. Dairy products and the risk of stroke and coronary heart disease: the Rotterdam study. Eur J Nutr. 2015 Sept;54(6):981–90.

12. Silva FM, Giatti L, Diniz MDFHS, Brant LCC, Barreto SM. Dairy product consumption reduces cardiovascular mortality: results after 8 year follow-up of ELSA-Brasil. Eur J Nutr. 2022 Mar;61(2):859–69.

13. Bonthuis M, Hughes MCB, Ibiebele TI, Green AC, Van Der Pols JC. Dairy consumption and patterns of mortality of Australian adults. Eur J Clin Nutr. 2010 June;64(6):569–77.

14. Goldbohm RA, Chorus AM, Galindo Garre F, Schouten LJ, Van Den Brandt PA. Dairy consumption and 10-y total and cardiovascular mortality: a prospective cohort study in the Netherlands. Am J Clin Nutr. 2011 Mar;93(3):615–27.

15. Sluik D, Boeing H, Li K, Kaaks R, Johnsen NF, Tjønneland A, et al. Lifestyle factors and mortality risk in individuals with diabetes mellitus: are the associations different from those in individuals without diabetes? Diabetologia. 2014 Jan;57(1):63–72.

16. Bongard V, Arveiler D, Dallongeville J, Ruidavets JB, Wagner A, Simon C, et al. Food groups associated with a reduced risk of 15-year all-cause death. Eur J Clin Nutr. 2016 June;70(6):715–22.

17. Tognon G, Nilsson LM, Shungin D, Lissner L, Jansson JH, Renström F, et al. Nonfermented milk and other dairy products: associations with all-cause mortality. Am J Clin Nutr. 2017 June;105(6):1502–11.

18. Farvid MS, Malekshah AF, Pourshams A, Poustchi H, Sepanlou SG, Sharafkhah M, et al. Dairy food intake and all-cause, cardiovascular disease, and cancer mortality. Am J Epidemiol. 2017 Apr 15;185(8):697–711.

19. Dehghan M, Mente A, Rangarajan S, Sheridan P, Mohan V, Iqbal R, et al. Association of dairy intake with cardiovascular disease and mortality in 21 countries from five continents (PURE): a prospective cohort study. The Lancet. 2018 Nov;392(10161):2288–97.

20. Pala V, Sieri S, Chiodini P, Masala G, Palli D, Mattiello A, et al. Associations of dairy product consumption with mortality in the European Prospective Investigation into Cancer and Nutrition (EPIC)–Italy cohort. Am J Clin Nutr. 2019 Nov;110(5):1220–30.

21. Mazidi M, Mikhailidis DP, Sattar N, Howard G, Graham I, Banach M. Consumption of dairy product and its association with total and cause specific mortality – a population-based cohort study and meta-analysis. Clin Nutr. 2019 Dec;38(6):2833–45.

22. Schmid D, Song M, Zhang X, Willett WC, Vaidya R, Giovannucci EL, et al. Yogurt consumption in relation to mortality from cardiovascular disease, cancer, and all causes: a prospective investigation in 2 cohorts of US women and men. Am J Clin Nutr. 2020 Mar;111(3):689–97.

23. Nakanishi A, Homma E, Osaki T, Sho R, Souri M, Sato H, et al. Association between milk and yogurt intake and mortality: a community-based cohort study (Yamagata study). BMC Nutr. 2021 Dec;7(1):33.

24. Sonestedt E, Borné Y, Wirfält E, Ericson U. Dairy consumption, lactase persistence, and mortality risk in a cohort from southern Sweden. Front Nutr. 2021 Nov 24;8:779034.

25. Lin P, Gui X, Liang Z, Wang T. Association of yogurt and dietary supplements containing probiotic consumption with all-cause and cause-specific mortality in US adults: a population-based cohort study. Front Nutr. 2022 Feb 7;9:803076.

26. Lu Y, Sugawara Y, Matsuyama S, Fukao A, Tsuji I. Association of dairy intake with all-cause, cancer, and cardiovascular disease mortality in Japanese adults: a 25-year population-based cohort. Eur J Nutr. 2022 Apr;61(3):1285–97.

27. Ge S, Zha L, Sobue T, Kitamura T, Iso H, Ishihara J, et al. Associations between dairy intake and mortality due to all-cause and cardiovascular disease: the Japan Public Health Center-based prospective study. Eur J Nutr. 2023 Aug;62(5):2087–104.

28. Miyagawa N, Takashima N, Harada A, Kadota A, Kondo K, Miura K, et al. Dairy intake and all-cause, cancer, and cardiovascular disease mortality risk in a large Japanese population: a 12-year follow-up of the J-MICC study. JAT. 2024;65049.

29. Zhang S, Li H, Engström G, Niu K, Qi L, Borné Y, et al. Milk intake, lactase persistence genotype, plasma proteins and risks of cardiovascular events in the Swedish general population. Eur J Epidemiol. 2023 Feb;38(2):211–24.

30. Khan M, Goto R, Kobayashi K, Suzumura S, Nagata Y, Sonoda T, et al. Dietary habits and cancer mortality among middle aged and older Japanese living in Hokkaido, Japan by cancer site and sex. APJCP. 2004;5:58–65.

31. Matsumoto M, Ishikawa S, Nakamura Y, Kayaba K, Kajii E. Consumption of dairy products and cancer risks. J Epidemiol. 2007;17(2):38–44.

32. Kojima M, Wakai K, Tamakoshi K, Tokudome S, Toyoshima H, Watanabe Y, et al. Diet and colorectal cancer mortality: results from the Japan collaborative cohort study. Nutr Cancer. 2004 Sept;50(1):23–32.

33. Tokui N, Yoshimura T, Fujino Y, Mizoue T, Hoshiyama Y, Yatsuya H, et al. Dietary habits and stomach cancer risk in the JACC study. J Epidemiol. 2005;15(Supplement_II):S98–108.

34. Ozasa K, Watanabe Y, Ito Y, Suzuki K, Tamakoshi A, Seki N, et al. Dietary habits and risk of lung cancer death in a large‐scale cohort study (JACC study) in Japan by sex and smoking habit. Japanese Journal of Cancer Research. 2001 Dec;92(12):1259–69.

35. Park Y, Mitrou PN, Kipnis V, Hollenbeck A, Schatzkin A, Leitzmann MF. Calcium, dairy foods, and risk of incident and fatal prostate cancer: the NIH-AARP diet and health study. Am J Epidemiol. 2007 Aug 28;166(11):1270–9.

36. Sakauchi F, Khan MMH, Mori M, Kubo T, Fujino Y, Suzuki S, et al. Dietary habits and risk of ovarian cancer death in a large-scale cohort study (JACCstudy) in Japan. Nutr Cancer. 2007 June 8;57(2):138–45.

37. Mann JI, Appleby PN, Key TJ, Thorogood M. Dietary determinants of ischaemic heart disease in health conscious individuals. Heart. 1997 Nov 1;78(5):450–5.

38. Fortes C, Forastiere F, Farchi S, Rapiti E, Pastori G, Perucci CA. Diet and overall survival in a cohort of very elderly people. Epidemiology. 2000;11(4):440–5.

39. Tognon G, Rothenberg E, Petrolo M, Sundh V, Lissner L. Dairy product intake and mortality in a cohort of 70-year-old Swedes: a contribution to the nordic diet discussion. Eur J Nutr. 2018 Dec;57(8):2869–76.

40. Ding M, Li J, Qi L, Ellervik C, Zhang X, Manson JE, et al. Associations of dairy intake with risk of mortality in women and men: three prospective cohort studies. BMJ. 2019 Nov 27;367:l6204.

41. Mills S. Drama, oratory and thucydides in fifth-century athens: Teaching imperial lessons [Internet]. Drama, Oratory and Thucydides in Fifth-Century Athens: Teach. Imp. Lessons. Taylor and Francis Inc.; 2020. 1 p. (Drama, Oratory and Thucydides in Fifth-Century Athens: Teaching Imperial Lessons). Available from: https://www.scopus.com/inward/record.uri?eid=2-s2.0-85104712736&doi=10.4324%2f9780351260322&partnerID=40&md5=a5ad64d44f03291e387a95dfb3e33077

42. Katagiri R, Sawada N, Goto A, Yamaji T, Iwasaki M, Noda M, et al. Association of soy and fermented soy product intake with total and cause specific mortality: prospective cohort study. BMJ. 2020 Jan 29;368:m34.

43. Nguyen HN, Miyagawa N, Miura K, Okuda N, Yoshita K, Arai Y, et al. Dietary tofu intake and long-term risk of death from stroke in a general population. Clinical Nutrition. 2018 Feb;37(1):182–8.

44. Hirayama T. Relationship of soybean paste soup intake to gastric cancer risk. Nutr Cancer. 1981 Jan;3(4):223–33.

45. Ngoan LT, Mizoue T, Fujino Y, Tokui N, Yoshimura T. Dietary factors and stomach cancer mortality. Br J Cancer. 2002 July;87(1):37–42.

46. Kurozawa Y, Ogimoto I, Shibata A, Nose T, Yoshimura T, Suzuki H, et al. Dietary habits and risk of death due to hepatocellular carcinoma in a large scale cohort study in Japan. Univariate analysis of JACC study data. Kurume Med J. 2004;51(2):141–9.

47. Nagata C, Wada K, Tamura T, Konishi K, Goto Y, Koda S, et al. Dietary soy and natto intake and cardiovascular disease mortality in Japanese adults: the Takayama study. The American Journal of Clinical Nutrition. 2017 Feb;105(2):426–31.

48. Rebello SA, Koh H, Chen C, Naidoo N, Odegaard AO, Koh WP, et al. Amount, type, and sources of carbohydrates in relation to ischemic heart disease mortality in a Chinese population: a prospective cohort study. Am J Clin Nutr. 2014 July;100(1):53–64.

49. Wada K, Oba S, Nagata C. Rice-based diet and cardiovascular disease mortality in Japan: from the Takayama study. Nutrients. 2022 May 30;14(11):2291.

50. Chow WH, Schuman LM, McLaughlin JK, Bjelke E, Gridley G, Wacholder S, et al. A cohort study of tobacco use, diet, occupation, and lung cancer mortality. Cancer Causes & Control. 1992;3(3):247–54.

51. Paganini-Hill A, Kawas CH, Corrada MM. Non-alcoholic beverage and caffeine consumption and mortality: the Leisure World cohort study. Preventive Medicine. 2007 Apr;44(4):305–10.

52. Zhong GC, Hu TY, Yang PF, Peng Y, Wu JJ, Sun WP, et al. Chocolate consumption and all-cause and cause-specific mortality in a US population: a post hoc analysis of the PLCO cancer screening trial. Aging. 2021 July 31;13(14):18564–85.

53. Zhao B, Gan L, Yu K, Männistö S, Huang J, Albanes D. Relationship between chocolate consumption and overall and cause-specific mortality, systematic review and updated meta-analysis. Eur J Epidemiol. 2022 Apr;37(4):321–33.

54. Sun Y, Liu B, Snetselaar LG, Wallace RB, Shadyab AH, Chen GC, et al. Chocolate consumption in relation to all-cause and cause-specific mortality in women: the women’s health initiative. JAND. 2023 June;123(6):902-911.e3.

55. Buijsse B, Feskens EJM, Kok FJ, Kromhout D. Cocoa intake, blood pressure, and cardiovascular mortality. Arch Intern Med. 2006;166:411–7.

56. Kwok CS, Boekholdt SM, Lentjes MAH, Loke YK, Luben RN, Yeong JK, et al. Habitual chocolate consumption and risk of cardiovascular disease among healthy men and women. Heart. 2015 Aug 15;101(16):1279–87.

57. Ho YL, Nguyen XMT, Yan JQ, Vassy JL, Gagnon DR, Gaziano JM, et al. Chocolate consumption and risk of coronary artery disease: the Million Veteran Program. Am J Clin Nutr. 2021 May;113(5):1137–44.
